# Hybrid performance in finger millet (*Eleusine coracana*) for grain yield and component traits under well-watered and drought-stress conditions

**DOI:** 10.3389/fpls.2026.1770566

**Published:** 2026-07-10

**Authors:** Adane Gebreyohannes, Hussein Shimelis, Jacob Mashilo, Fatma Sarsu

**Affiliations:** 1 African Centre for Crop Improvement, School of Agricultural, Earth and Environmental Sciences, University of KwaZulu-Natal, Scottsville, South Africa; 2 Ethiopian Institute of Agricultural Research, Melkassa Agricultural Research Center, Adama, Ethiopia; 3 Agricultural Research Council-Small Grain Institute, Bethlehem, South Africa; 4 Plant Breeding and Genetics Section, Department of Nuclear Sciences and Applications, International Atomic Energy Agency (IAEA), Vienna International Centre, Vienna, Austria

**Keywords:** combining ability analysis, drought tolerance, Ethiopia, finger millet, gene action, line x tester analysis

## Abstract

**Introduction:**

Finger millet (*Eleusine coracana*) is a high-potential, climate-resilient and nutrient-rich small cereal with growing potential for food, feed, and other value-added products across various market segments. Knowledge on the combining ability and heterosis of breeding populations under contrasting water regimes is essential for genetic improvement of the crop for economics traits. The aim of this study was to determine the combining ability, heterosis, and the nature of gene action of selected Ethiopian finger millet parents for economic traits in hybrid combinations to guide the selection of best families and new breeding populations.

**Methods:**

A 7 × 10 line × tester mating design was used, and the resultant 70 F_1_ hybrids and 17 parents were evaluated under non-stressed (NST) and drought-stress (ST) conditions across greenhouse and field environments. Genotypes were profiled for major agronomic traits: plant height (PTH), days to 50% flowering (DTF), days to maturity (DTM), number of productive tillers per plant (NT), primary finger length (FL), ear length (EL), number of fingers per ear (NF), grain yield (GY), harvest index (HI), and thousand seed weight (TSW).

**Results:**

Line (GCA_L_), tester (GCA_T_), and line × tester (SCA) mean squares were significant (p < 0.001) for all assessed traits, indicating contributions of both additive and non-additive gene actions in the inheritance of agronomic and physiological traits. Grain yield declined by 57% in parental genotypes and 62% in F_1_ hybrids under drought stress; however, several superior crosses exhibited relatively lower yield penalties (-51%), indicating enhanced drought resilience. Additive genetic effect predominantly conditioned the inheritance of DTF, EL, DTM under ST conditions, whereas non-additive effects were more important for NT and DTM under NST conditions. Broad-sense heritability (H^2^) for GY was higher under ST (0.51) than NST (0.13) conditions, necessitating multiple testing environments for drought tolerance evaluation and selection. Lines such as G3, G2, and G4 and testers such as G14, G8, G13, G17, and G10 exhibited higher general combining ability effects for GY in a desirable trend, in that order. Furthermore, crosses G85, G31, G79, G38, G48, G54, G64, and G65 exhibited higher specific combining ability effects and heterosis for GY under ST conditions.

**Discussion:**

The results demonstrated substantial genetic variability for grain yield and related traits with additive and non-additive gene actions. This guides effective selection based on additive and non-additive gene effects in finger millet improvement. The selected parents and the top crosses are recommended for breeding and selecting new-generation, drought-adapted finger millet genotypes.

## 1 Introduction

Finger millet (*Eleusine coracana* [L.] Gaertn.) is a high-potential, climate-smart, and nutrient-rich small cereal crop. Africa is the center of origin and genetic diversity of the crop, where it is used as food and livestock feed and for value-added household and industrial products (Tsehaye et al., 2006; Tesfaye and Mengistu, 2017). Finger millet is gluten-free and rich in calcium, iron, zinc, dietary fiber, essential amino acids, and bioactive compounds such as phenolics, tannins, flavonoids, and antioxidants (Gaikwad et al., 2024; Sirin, 2023; Gebreyohannes et al., 2024a, 2024b; Shimelis et al., 2026). The nutritional value of finger millet makes it a functional food with human health benefits against diabetes, anemia, osteoporosis, and obesity (Puranik et al., 2020; Maharajan et al., 2022; Backiyalakshmi et al., 2023). Finger millet grain is processed into specialty alcoholic and non-alcoholic beverages such as distilled spirits or domestic drinks (Lule et al., 2012; Mukisa et al., 2012). Hence, it enhances livelihoods and drives market diversification.

Finger millet is drought-tolerant and possesses a C4 photosynthesis system with efficient photo-assimilation and biomass production (Yogeesh et al., 2016; Brhane et al., 2017). The grain is resilient to storage pests, enabling long-term preservation under traditional storage conditions with minimal damage. Also, the genetic plasticity and adaptation to grow under harsh environmental conditions make it a crop of choice in marginal agroecologies in sub-Saharan Africa and South Asia (Mbinda and Mukami, 2021; Mbinda et al., 2021). Globally, finger millet accounts for approximately 20% of the total millets production with an estimated area of ~2.1 million ha and 26% of total production (~3.7 million tons) annually (FAOSTAT, 2022; Indiastat, 2022; Gebreyohannes et al., 2024b). India and Ethiopia are the major contributors to finger millet production in the world (FAOSTAT, 2022; Indiastat, 2022; Gebreyohannes et al., 2024b). In Ethiopia, finger millet is cultivated on approximately 480,511 ha of land with a total annual yield output of 1,218,582 tons (CSA, 2020). Ethiopia accounts for 22% (global) and 53% (African) of finger millet cultivation area and 33% (global) and 73% (African) of total production, indicating the value of finger millet in the agricultural sector (Gebreyohannes et al., 2024b).

Despite its economic importance, finger millet productivity remains considerably low (<2.5 t/ha) in the major producing regions such as Ethiopia, far below its attainable yield potential of 6 to 8 t/ha (CSA, 2017; Gebreyohannes et al., 2021, 2024b). The yield gap is attributed to an array of biotic, abiotic, and socioeconomic constraints and limited genetic improvement (Mulualem and Melak, 2013; Owere et al., 2014; Mbinda et al., 2021; Tarekegne et al., 2021). Among the abiotic challenges, recurrent drought associated with climate change is the leading finger millet production constraint, causing estimated yield loss ranging from 50% to 100% crop failure (Maqsood and Ali, 2007; Gebreyohannes et al., 2025a). In Ethiopia, there are a limited number of drought-tolerant finger millet varieties available to growers for sustained and profitable production. Consequently, developing high-yielding and drought-tolerant varieties remains a key objective of Ethiopian finger millet breeding programs. However, breeding progress has been constrained by a limited understanding of the genetic architecture of key agronomic and physiological traits.

In this context, the line × tester (L × T) mating design provides a robust framework for dissecting the genetic basis of traits and evaluating the breeding potential of parental lines and their cross combinations (Acquaah, 2012). This approach enables partitioning of genetic variance into general combining ability (GCA) and specific combining ability (SCA), thereby facilitating the dissection of additive and non-additive gene effects (Mapako and Maphosa, 2025). GCA represents the heritable component of genetic variance that can be accumulated through selection. Conversely, non-additive genetic effects are associated with SCA attributable to dominance and epistatic genes and their interactions. The SCA effects contribute to heterosis or hybrid vigor (Li et al., 2008; Liang et al., 2015; Viana, 2023).

Understanding the relative importance of additive and non-additive gene action is critical in drought-prone production systems, where the choice of breeding methods is either selection-based or hybrid-oriented. Integrating L × T analysis with multienvironment evaluation under both non-stressed and drought-stressed conditions enhances the accuracy of genotype performance assessment and facilitates the identification of stable, high-performing genotypes. This approach has been successfully applied in several crops, including finger millet, pearl millet, and foxtail millet, to elucidate the genetic architecture of traits related to yield, maturity, and nutrient composition (Priyadharshini et al., 2015; Thribhuvan et al., 2023; Mapako and Maphosa, 2025). Agronomic traits including flowering and maturation times, plant height, panicle length and width, seed weight, and physiological traits (i.e., stay-green trait and chlorophyl content) are associated with drought adaptation and yield potential. However, there remains limited information on combining ability, gene action, and heterosis in Ethiopian finger millet germplasm for these economic traits under contrasting non-stressed environments. Therefore, the objective of this study was to determine the combining ability, heterosis, and the nature of gene action of selected finger millet parents for economic traits in hybrid combinations to guide the selection of the best families and new breeding populations. We hypothesize that Ethiopian finger miller accessions possess significant combining ability and heterosis for agronomic and physiological traits under contrasting environmental conditions.

## 2 Materials and methods

### 2.1 Plant materials

A total of 17 finger millet genotypes, comprising 7 lines (female parents) and 10 testers (male parents), were used in the study. The 17 parents were selected after rigorous phenotyping and genotyping (Gebreyohannes et al., 2025a, 2025b). The parents have contrasting agronomic performance and unique nutritional profiles (**Table 1**). The lines have complementary traits, including early flowering and maturity, as well as high nutritional composition, like high iron, zinc, starch, and amylose contents. The four popular and widely cultivated late-maturing varieties (i.e., Addis-01, Wama, Necho, and Tessema) were used as lines due to their wide adaptability to various agroecologies of the country, whereas the three early-maturing lines were selected possessing short plant stature, longer retention of green foliage (i.e., stay-green), and lodging resistance. The 10 complementary testers were selected, possessing desirable attributes such as early maturity, seed quality, and disease resistance.

**Table 1:** Names and descriptions of the finger millet parental genotypes used in the cross. .

#	Name or designation	Codes	Role in the cross	Nutritional attributes	Agronomic traits
1	227974	G1	Line	High iron and zinc contents	Late-maturing and susceptible to *Striga*
2	234187	G2	Line	High amylose content	Early maturing, stay-green foliage, and stem lodge resistance
3	235700	G3	Line	High iron and zinc contents	Early maturing and short plant stature
4	Addis-01	G4	Line	High amylose and starch contents	Late maturing, high-yielding
5	Necho	G5	Line	High amylose, and starch contents	White seeded, lodging resistant, late maturing, susceptible to blast
6	Tessema	G6	Line	High amylose, and starch	Lodging resistance and late maturing
7	Wama	G7	Line	High starch content	*Striga* resistant and late maturing
8	24394	G8	Tester	High starch content	Stay green trait, disease resistance, lodging resistance, and anthocyanin pigmentation on stem
9	213835	G9	Tester	High starch content	Stay green trait, disease resistance, lodging resistance, and anthocyanin pigmentation on stem
10	215982	G10	Tester	High iron and zinc contents	High number of fingers
11	238335	G11	Tester	High starch content	Early maturing
12	AxumColl21	G12	Tester	High amylose and starch contents	*Striga* resistant
13	Bako-09	G13	Tester	High amylose	Stay-green trait, anthocyanin pigmentation
14	Gute	G14	Tester	High amylose, and starch contents	Stay-green trait, and lodging resistance
15	Kako-1	G15	Tester	High amylose and starch contents	Stay green trait, short plant stature, lodging resistance, and anthocyanin pigmentation
16	Meba	G16	Tester	High amylose contents	Stay-green trait, disease resistance, anthocyanin pigmentation
17	Mereb-1	G17	Tester	High amylose and starch contents	Stay-green trait, early maturing, anthocyanin pigmentation

### 2.2 Hybrid development

**Figure 1:**
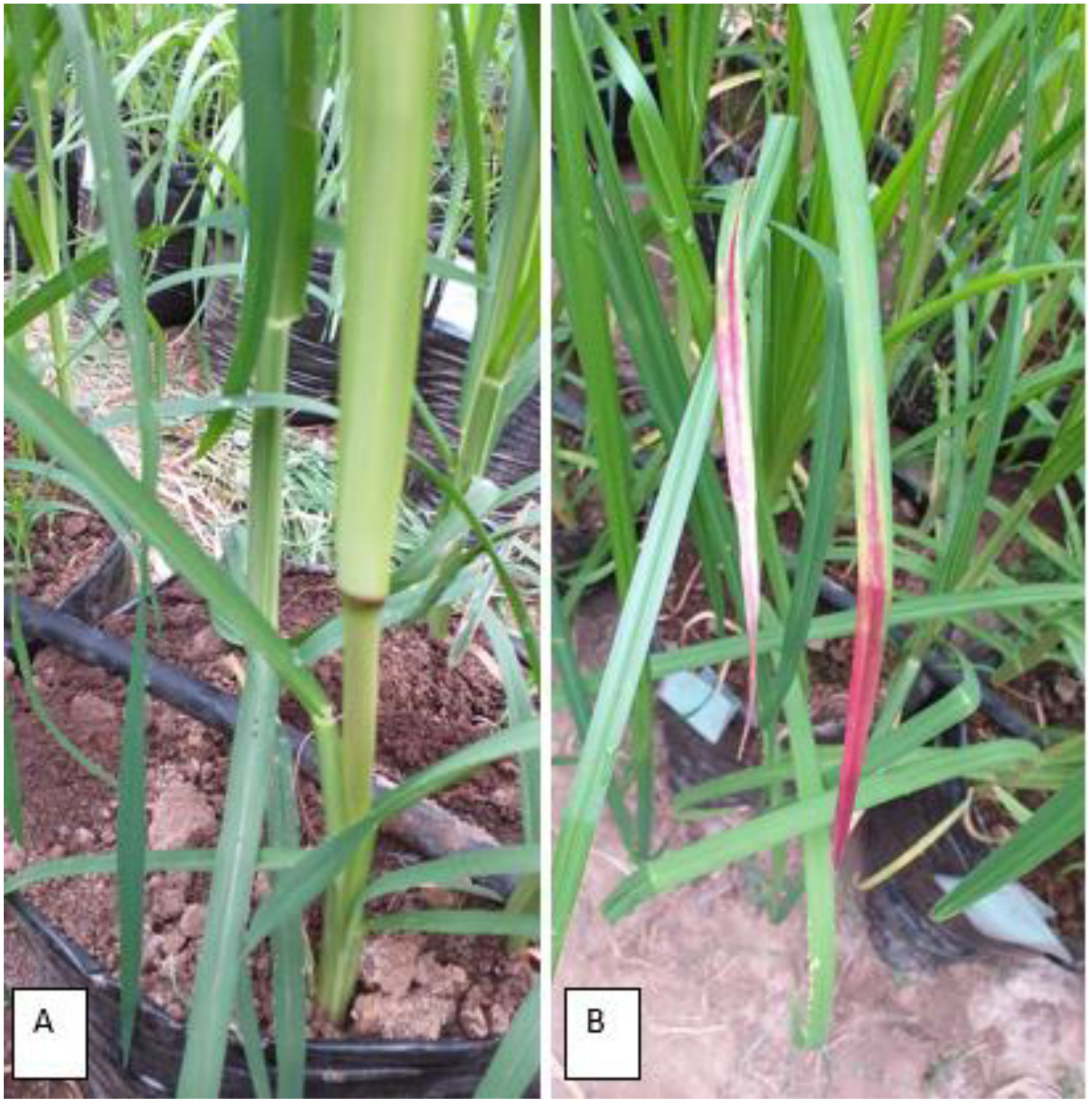
Distinct dominant morphological markers used for male parents (testers) used for F_1_ hybridity confirmation: stem node pigmentation **(A)** and leaf pigmentation **(B)**.

The 7 lines and 10 testers were stagger-planted three times in a 2-week interval at Melkassa Agricultural Research Centre in Ethiopia in 2021. Stagger-planting allowed the synchronization of panicle development and flowering time for hot water emasculation and controlled pollination. At anthesis, panicles were covered with glassine paper bags. Sampled panicles were prepared by treating them in 52 °C hot water in a plastic jug for 2 min to sterilize the pollen grains in the florets. Treated panicles were bagged with glassine paper to prevent from foreign pollens. After 3–4 days, when the stigma appeared fluffy, controlled pollination was done using plants with freshly dehisced pollen from the designated tester. Controlled pollination was performed by carefully intertwining the fingers of the tester panicle with the fingers of the emasculated florets of the line’s panicle. After pollination, the panicle of the female or the line was secured by covering with a labeled glassine paper bag to prevent uncontrolled cross-pollination. Crosses were made using 7 lines × 10 testers mating design, whereby each line was crossed with all testers. Anthocyanin pigmentation is a dominant trait in millets (Renganathan et al., 2021), and thus, true hybridity in the present study was confirmed using male parental testers carrying this distinct morphological marker, enabling clear identification of F_1_ progenies. Selfed or off-type genotypes were rigorously rouged out at the seedling stage, and only verified true F1 plants were tagged and used for subsequent evaluation (**Figure 1**). This procedure resulted in the development of 70 F_1_ hybrids (**Supplementary Table 1**), which, together with 17 parental genotypes, constituted the 87 total genotypes used for subsequent analyses.

### 2.3 Experimental setup and trial management

The F_1_ hybrids and the parents were evaluated at the Melkassa Agricultural Research Centre (39°21′E, 8°24′N, 1,550 m asl) in 2021 under non-stressed (NST) and drought-stressed (ST) conditions in greenhouse and field environments. An experimental design with 29 rows × 3 columns and 87 genotypes in two replications was used at both sites.

#### 2.3.1 Field

The field experiment was conducted during the off-season under rain-free conditions, enabling control of water supply. Consequently, no rainfall was recorded during the experimental period, and drought stress was imposed exclusively through furrow irrigation using a locally made and calibrated Parshall flume. Each genotype was planted in 3-m rows with 40-cm interrow spacing, maintaining 20 plants per plot. Stressed and non-stressed plots were separated by 10 m to prevent lateral flow. Non-stressed plots were irrigated every 6 days to maintain ~80% FC, whereas stressed plots received no water after flowering to impose controlled drought. Soil water status was monitored using tensiometers and installed at various depths across the field, and crop water requirement (CWR) was calculated. The CWR in the field experiment was estimated using the formula CWR = KC × ETo, where KC is the crop coefficient determined at different growth and developmental stages whereas ETo is the reference crop evapotranspiration. ETo was computed using CROPWAT 8.0 (FAO, 1998), and climate data were acquired from a local meteorological station collected by the National Meteorological Agency of Ethiopia (**Table 2**).

**Table 2:** Description of environmental conditions for field and greenhouse experiments.

Parameter	Test sites	
	Field	Greenhouse
Soil texture	Loam	Loam
pH	7.3	7.0
Annual rainfall (mm/year)	582.1	–
Maximum temperature (°C)	29.5	52.0
Minimum temperature (°C)	13.8	13.5
Relative humidity (%)	53.1	65.0
Wind speed (km/day)	2.2	–
Sunshine (h)	8.4	-

#### 2.3.2 Greenhouse

Under the greenhouse environment, polyethylene plastic pots of 20-L capacity (i.e., 300-mm diameter and 280-mm height) were used. In each experimental unit, >30 seeds were sown and later thinned to 20 vigorous plants per pot after 2 weeks of emergence. The pot plants represent an equivalent number to a single plot of a field experiment. A drip irrigation system with 0.42 L h^−1^ emitters supplied water from a reservoir through regulated main and sub-lines. Soil moisture was monitored gravimetrically to maintain ~80% field capacity (FC) in non-stressed pots, whereas irrigation was withheld at flowering in stressed pots until moisture declined to ~30% FC, simulating post-flowering drought.

In both growing conditions, standard agronomic practices were also applied consistently across environments, including application of diammonium phosphate (DAP) at 100 kg/ha at planting, manual weeding, and insect pest control with recommended practices for the study areas.

### 2.4 Data collection

Data on agronomic traits, including agro-morphological and drought tolerance characteristics, were collected following modified International Board for Plant Genetic Resources (IBPGR) (1985) guidelines. Plant height (PTH, cm) was measured from the base of the plant to the tip of the head (ear) at the dough stage. Days to 50% flowering (DTF) and days to maturity (DTM) were recorded as days from sowing to when 50% of main tillers had emerged (flowering) and reached physiological maturity, respectively. The number of basal tillers with productive or mature ears (NT), length of the longest finger (FL, cm), and ear length (EL, cm) were measured at the dough stage on the main tiller. The number of fingers on the main ear (NF) was counted. Grain yield (GY) was computed from ~20 plants per pot per replication in the greenhouse (polyethylene pots, 300 mm diameter × 280 mm height; plot size = 0.071 m²) and from all plants per plot per replication ~ 20 plants (1 row × 3 m; 0.4 m row spacing). In both experiments, GY was adjusted to 12.5% moisture content and expressed in t/ha by scaling the measured yield to the corresponding pot or plot area. Harvest index in % (HI) was calculated as the ratio of grain yield to biological yield per pot (plot) multiplied by 100%. Thousand seed weight (TSW) was determined by drying 1,000 randomly selected seeds to a constant moisture content of 12.5% and recording their weight. Finally, chlorophyll content at maturity was measured at three points in mature flag leaves and averaged using a soil and plant analysis development [SPAD] meter (502 Minolta Co., Osaka, Japan) (Rad et al., 2012).

### 2.5 Data analysis

#### 2.5.1 Estimates of variance components

Variance components were partitioned, and combining ability effects were computed using a two-way analysis of variance (ANOVA) procedure in SAS PROC GLM (SAS, 2013). The general combining ability (GCA) and specific combining ability (SCA) variances were computed following the L × T genetic design (Kempthorne, 1957), as outlined by Singh and Chaudhary (1985). Genotypic variation among stressed and non-stressed conditions within each testing site was assessed, and homogeneity of error variances was assessed using Bartlett’s test (Glass, 1996). In the ANOVA model, lines, testers, and L × T were treated as random effects, whereas environment (site), water regime, and replication were treated as fixed effects The following linear mixed model was adopted for ANOVA:


$${\text{Y}}_{\text{ijlm}}=\text{μ}+{\text{W}}_{\text{m}}+{\text{R}}_{\text{l}(\text{m})}+{\text{L}}_{\text{i}}+{\text{T}}_{\text{j}}{\left(\text{LT}\right)}_{\text{ij}}+{\left(\text{LW}\right)}_{\text{im}}+{\left(\text{TW}\right)}_{\text{jm}}+{\left(\text{LTW}\right)}_{\text{ijm}}+{\text{ϵ}}_{\text{ijlm}}$$

Y_ijlm_​ is the average performance of the cross between the ith line and the jth tester, tested under the mth water regime, and in the lth replication; μ is the overall mean of all observations; W_m_​ is the fixed effect of water regime. R_l(m)_ represents the effect of replication, which is nested within the water regime. L_i_​ and ith are the random effects of the lines and testers, showing their general combining ability (GCA). The interaction (LT)_ij_​ represents the random effect of specific combining ability between lines and testers. Other interactions include lines × water regimes (LW), and testers × water regimes (TW). Higher-order interactions are also included: SCA × water regimes (LTW), and the three-way interaction of lines × testers × water regimes (LTW). Finally, ϵ_ijlm_ is the residual error term, which represents random variation not explained by the model. It accounts for unexplained variations in each observation defined by the ith line, jth tester, mth water regime, and lth replication. This error is assumed to be independent and normally distributed with variance σ².

The significance of GCA for lines and testers, and SCA for crosses, was assessed using a two-tailed t-test. Error mean squares were used to estimate the standard errors associated with each parameter, enabling statistical analysis of GCA and SCA effects within each treatment as follows:


$$\text{t statistic }=\frac{Effect}{SE}$$

where SE is the standard error of the difference.

The following formula was used for the calculation of standard errors (SE) for combining ability effects:

$\text{SE of GCA for lines }=\sqrt{MSE/rt}$, $\text{SE of GCA for testers }=\sqrt{MSE/rl}$, $\text{SE of SCA effects }=\sqrt{MSE/r}\;$

where *MSE* = mean square error from the analysis of variance table. r, t, and l are the number of replications, testers, and lines, respectively.

A standard ANOVA of the line × tester mating design was conducted to estimate overall genotypic variance. In cases where genotypic effects were significant, the line × tester interaction was analyzed to determine lines with superior SCA, indicated by differential performance across tester genotypes (Singh and Chaudhary, 1985). The significance of testers [GCA (T)] and lines [GCA (L)] mean squares within each site was assessed using the line × tester interaction as the error term, whereas the significance of the line × tester interaction (SCA) was evaluated using the error mean square. **Table 3** outlined the ANOVA model used to analyze the line by tester design when data from all testing sites and water regimes are pooled.

**Table 3:** Structure of combined analysis of variance and expected mean squares for line × tester mating design across water regimes.

Source of variations	Degree of freedom	Mean squares (MS)	Expected mean squares (EMS)
Replications	r-1		
Water regimes (W)	w-1		
Lines (L)	l-1	MS_L_	σ^2^ + rσ^2^_LTW_ + rwσ^2^_LT_ + rtσ^2^_LW_ + rtwσ^2^_L_
Testers (T)	t-1	MS_T_	σ^2^ + rσ^2^_LTW +_ rwσ^2^_LT +_ rlσ^2^_TW +_ rlwσ^2^_T_
L × T	(l-1) (t-1)	MS_LT_	σ^2^ + rσ^2^_LTW_ + rwσ^2^_LT_
L × W	(l-1) (w-1)	MS_LW_	σ^2^ + rσ^2^_LTW_ + rtσ^2^_LW_
T × W	(t-1) (w-1)	MS_TW_	σ^2^ + rσ^2^_LTW_ + rlσ^2^_TW_
L × T × W	(l-1) (t-1) (w-1)	MS_LTW_	σ^2^ + rσ^2^_LTW_
Error	w(rlt-l-t-1) + l + t - lt	MS_E_	σ^2^

r, w, l, and t are the number of replications, water regimes, lines, and testers, respectively.

The proportional contribution of the genetic effects of lines, testers, and interactions with lines, testers, and sites was computed as follows:


Contribution of lines(%)=sum of square of(Lines/Crosses)x100

Contribution of testers(%)=sum of square of(testers/crosses)x100

#### 2.5.2 Estimates of genetic variance

Estimates of genetic variance components for various parameters were derived by equating the observed mean squares with the expected mean squares associated with each variable in the ANOVA model across testing sites and water regimes, as defined below:


$${\text{σ}}^{2}L_{=}\frac{{\text{MS}}_{L}-\;{\text{MS}}_{LT}\;-\;{\text{MS}}_{LW\;}+\;{\text{MS}}_{LTW\;}}{rtw}={\text{σ}}^{2}G_{C}A_{L}$$

$${\text{σ}}^{2}T_{=}\frac{{\text{MS}}_{T}\;-\;{\text{MS}}_{LT\;}-\;{\text{MS}}_{TW\;}+\;{\text{MS}}_{LTW\;}}{rlw}={\text{σ}}^{2}G_{C}A_{T}$$

$${\text{σ}}^{2}L_{T}=\frac{{\text{MS}}_{LT\;}-\;{\text{MS}}_{LTW}\;}{rw}={\text{σ}}^{2}{SC}_{A}$$

$$\frac{({\text{σ}}^{2}L_{+}{\text{ σ}}^{2}T_{)}}{2}={\text{CovHS}}_{ave}={\text{σ}}^{2}{GC}_{A}$$

$${\text{σ}}^{2}{GC}_{A}=Cov\;HS_{ave}=(\frac{1+F}{4}){\text{σ}}^{2}A_{;}\;{\text{σ}}^{2}{SC}_{A}=(\frac{1+F}{2}){\text{σ}}_{2}^{D}$$

where ${\text{σ}}^{2}L_{,}\;{\text{σ}}^{2}T_{,}{\text{σ}}^{2}A_{,}\;\;{\text{σ}}^{2}D_{,}{\text{σ}}^{2}{GCA}_{L},\;{\text{σ}}^{2}{GCA}_{T},{\text{σ}}^{2}{SC}_{A},\;{\text{and σ}}^{2}L_{T},\;$ are variances due to lines, testers, additive genetic effects, dominance genetic effects, GCA of lines, GCA of testers, specific combining ability effects, and lines × testers interaction, respectively. $\;{\text{MS}}_{L},{\text{ MS}}_{T},{\text{ MS}}_{E},{\text{ MS}}_{LT},{\text{MS}}_{LW},{\text{MS}}_{TW},{\text{and MS}}_{LTW}$ are the mean square due to lines, testes, error, interactions of lines × testers, lines ×water regimes, testers × water regimes, and lines × testers × water regimes, and r, t, l, w are the number of replications, testers, lines, and water regimes, respectively. Assuming complete homozygosity (F = 1) for both lines and testers, additive genetic variance ( ${\text{σ}}_{2}^{A}$) and dominance genetic variance ( ${\text{σ}}^{2}D_{)}$ were estimated using the full observed phenotypic variation.

The phenotypic distribution and variability of morpho-physiological traits across contrasting moisture regimes were evaluated using multipanel raincloud and box-violin plots generated via the *ggplot2* (Wickham, 2016) and *ggbeeswarm* packages, integrating mean, range, and standard error to capture the extent of transgressive segregation and environmental plasticity as previously detailed in crop stress-response studies (Matthews, and Marshall-Colón, 2021).

#### 2.5.3 Genetic parameters

Baker’s ratio measures the comparative importance of additive (general combining ability, GCA) against non-additive (specific combining ability, SCA) genetic effects in a breeding population. It was computed from variance components of GCA and SCA, as defined by Baker (1978). Broad-sense heritability (H²) of traits was computed based on the combined analysis of variance according to Gowda et al. (2012).

#### 2.5.4 Mid-parent and better-parent heterosis

Heterosis was quantified as the percentage deviation from both mid-parent (MP) and better-parent (BP) values, following Falconer and MacKay (1996). The significance of heterosis of crosses was assessed using a two-tailed t-test with degrees of freedom reflecting the number of crosses. Error mean squares were utilized to estimate the standard errors associated with each parameter, enabling statistical analysis of the effects for heterosis as follows: $\text{t statistic }=\frac{Effect}{SE}$, where SE is the standard error. The SE for heterosis effects was calculated as follows: $\text{SE of heterosis of crosses }=\sqrt{MSE/r}$, where *MSE* = mean square error of crosses from the analysis of variance table and r is the number of replications. The heterosis for 70 F1 finger millet crosses was also visualized across moisture regimes using a faceted heatmapping approach in R with *ggplot2* and *tidyverse* packages (Wickham et al., 2019), allowing for a high-resolution pictorial comparison of genotypic stability and trait-specific heterotic expression as advocated in complex phenotypic studies (Fiévet et al., 2018).

## 3 Results

### 3.1 Analysis of variance and mean trait performance of finger millet genotypes under contrasting moisture regimes

Under drought-stressed (ST) conditions, the analysis of variance showed highly significant (P <0.001) differences among progenies (F_1_) for all assessed traits (**Table 4**). Conversely, the water regimes (W) effect was highly significant for plant height (PTH), days to 50% flowering (DTF), days to maturity (DTM), grain yield (GY), harvest index (HI), and chlorophyl content at maturity (SPADM), whereas number of tillers (NT) and ear-related traits were largely non-significant. The F_1_ × W interactions were highly significant (P <0.001) for the majority of traits. Parents also displayed significant differences for all assessed traits except number of fingers (NF) (**Table 4**). Similarly, under non-stressed (NST) growing conditions, F_1_ progenies showed highly significant variation (P < 0.001) across all assessed traits. The W effect was significant for PTH, DTF, NT, DTM, GY, HI, thousand seed weight (TSW), and SPADM, whereas ear length (EL) and finger length (FL) remained non-significant. The F_1_ × W interaction was significant for most traits, notably PTH, DTF, EL, FL, NF, HI, TSW, and SPADM, whereas NT and GY revealed non-significant interaction effects. Parents had significant differences across all evaluated traits under NST conditions (**Table 4**).

**Table 4:** Analysis of variance showing mean square and significant tests for agronomic and physiological traits in finger millet genotypes evaluated under drought-stressed and non-stressed conditions.

Sources of variation	Growing condition	df	Traits										
			PTH	DTF	EL	FL	NT	NF	DTM	GY	HI	TSW	SPADM
			Mean squares										
Progenies (F_1_)	ST	69	414.2**	191.7**	17.0**	13.3**	2.2**	5.9**	180.7**	2.1**	45.5**	0.4**	66.5**
	NST	69	501.3**	173.6**	31.8**	22.5**	7.3**	8.7**	403.0*	6.5**	43.6**	0.3**	66.8**
Water regimes (W)	ST	1	5,263.6**	88,253.0**	14.7^NS^	2.9^NS^	2.1^NS^	0.8^NS^	60,182.2**	207.6**	3,235.8**	0.2^NS^	2,562.8**
	NST	1	1,479.4**	70,774.1**	2.1^NS^	1.7^NS^	10.3*	10.8^NS^	30,565.8**	1276.6**	9,419.8**	1.0**	4,982.9**
F_1_ × W	ST	69	212.7**	160.1**	6.6^NS^	5.5^NS^	1.0*	4.5**	127.8**	1.6**	32.3**	0.1**	49.9**
	NST	69	357.9**	147.0**	14.0**	9.4**	1.8^NS^	6.4**	311.7^NS^	6.6**	37.8**	0.4**	77.4**
Columns (Rep)	ST	2	133.8^NS^	5.2^NS^	2.4^NS^	4.3^NS^	1.0^NS^	1.3^NS^	58.7^NS^	0.3^NS^	12.9^NS^	0.0^NS^	17.0^NS^
	NST	2	91.5^NS^	78.6*	8.3^NS^	10.9*	2.4^NS^	6.3^NS^	273.0^NS^	6.8*	29.4*	0.1^NS^	16.3^NS^
Rows	ST	28	116.6^NS^	23.3^NS^	5.7^NS^	4.2^NS^	0.8^NS^	2.2^NS^	70.9^NS^	0.2^NS^	5.7^NS^	0.1^NS^	10.6^NS^
	NST	28	62.3^NS^	31.9^NS^	5.8^NS^	4.1^NS^	2.4^NS^	3.6^NS^	200.9^NS^	1.7^NS^	6.8^NS^	0.1^NS^	27.9^NS^
Replications (W)	ST	1	237.7^NS^	10.0^NS^	56.2*	39.0*	0.7^NS^	13.3*	0.6^NS^	0.0^NS^	9.5^NS^	0.0^NS^	36.0^NS^
	NST	1	542.0**	163.8*	31.4*	10.8^NS^	0.6^NS^	1.1^NS^	834.4^NS^	53.2**	72.9**	0.6**	50.0^NS^
Parents	ST	16	518.0**	289.7**	23.5**	17.1**	1.5*	5.3^NS^	202.4**	2.9**	129.0**	0.5**	67.9**
	NST	16	805.7**	141.5**	49.4**	25.3**	5.4**	10.2**	741.1**	7.0*	37.2**	1.0**	98.4**
Error	ST	139	112.2	23.8	8.4	6.5	0.7	2.5	62.2	0.2	4.9	0.1	14.6
	NST	139	71.4	26.7	5.9	4.5	2.1	3.8	267	2.7	10.8	0.1	23.6

* and ** = significant at p<0.05 and 0.01, respectively. df, degree of freedom; PTH, plant height, cm; DTF, days to flowering; EL, ear length, cm; FL, length of the longest finger, cm; NT, number of productive basal tillers per main plant; NF, the number of fingers on the main ear; DTM, days to maturity; GY, grain yield, t/ha; HI, harvest index (%); TSW, thousand seed weight (g); SPADM, chlorophyll content, chlorophyll content at maturity.

**Figure 2:**
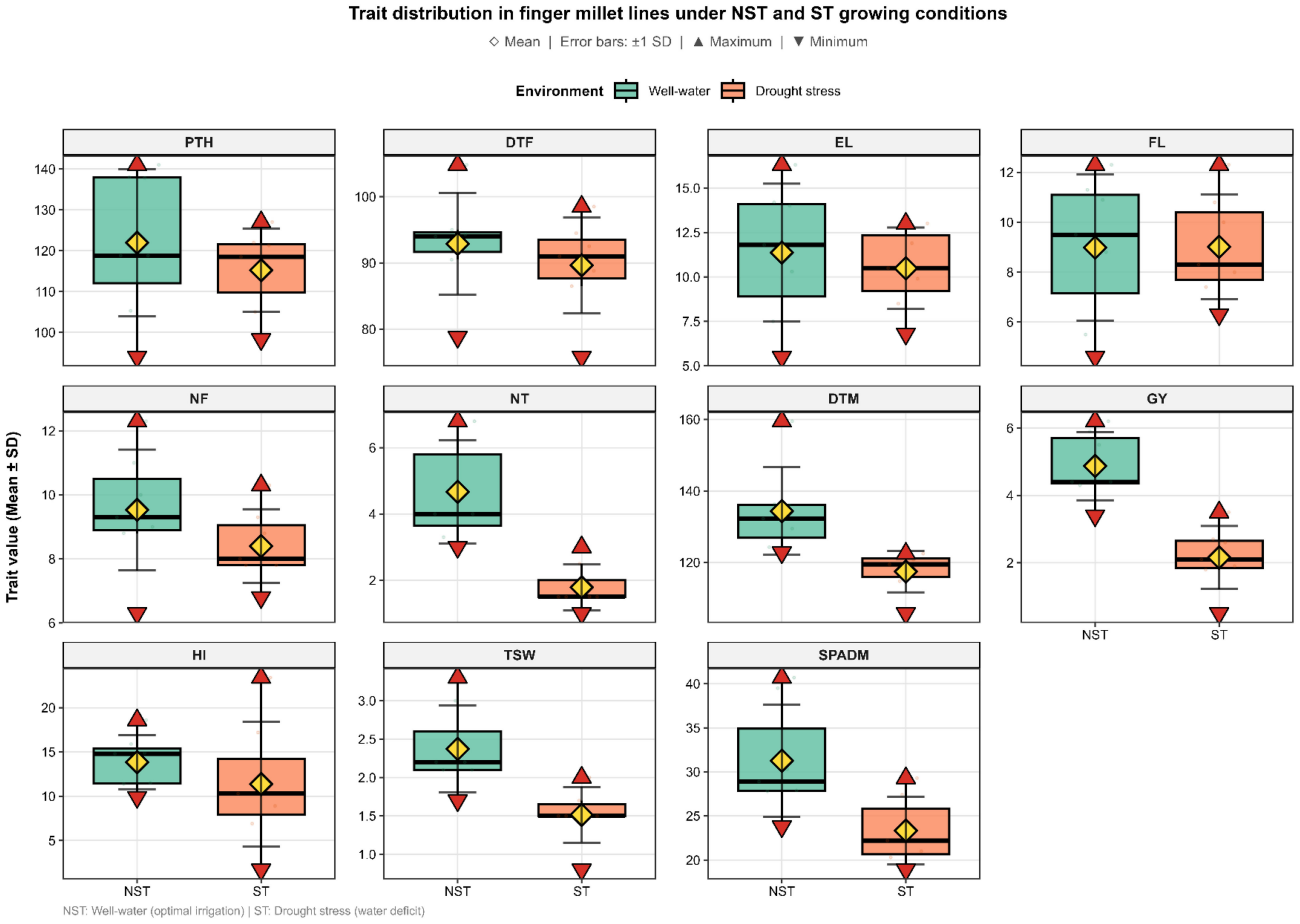
Distribution of agronomic and physiological traits in seven finger millet parental lines under stress (ST) and non-stress (NST) growing conditions across glasshouse and field environments. Notes: PTH, plant height (cm); DTF, days to flowering; EL, ear length (cm); FL, length of the primary finger (cm); NT, number of productive tillers per plant; NF, the number of fingers on the primary ear; DTM, days to maturity; Gy, grain yield (t/ha); HI, harvest index (%); TSW, thousand seed weight (g); SPADM, chlorophyll content at maturity.

**Figure 3:**
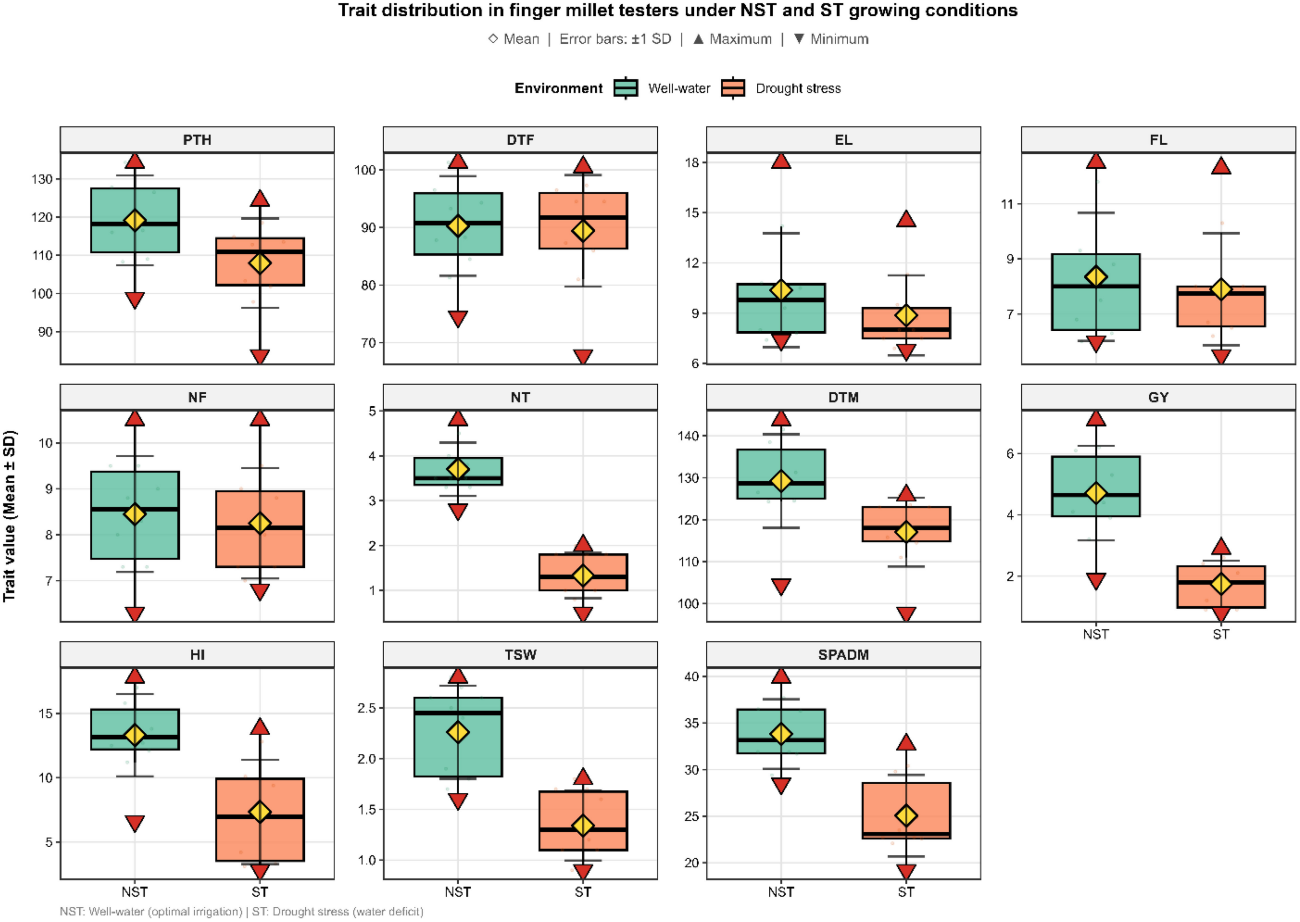
Distribution of agronomic and physiological traits in 10 finger millet testers under stress (ST) and non-stress (NST) growing conditions across glasshouse and field environments. Notes: PTH, plant height (cm); DTF, days to flowering; EL, ear length (cm); FL, length of the primary finger (cm); NT, number of productive tillers per plant; NF, the number of fingers on the primary ear; DTM, days to maturity; GY, grain yield (t/ha); HI, harvest index (%); TSW, thousand seed weight (g); SPADM, chlorophyll content at maturity.

**Figure 4:**
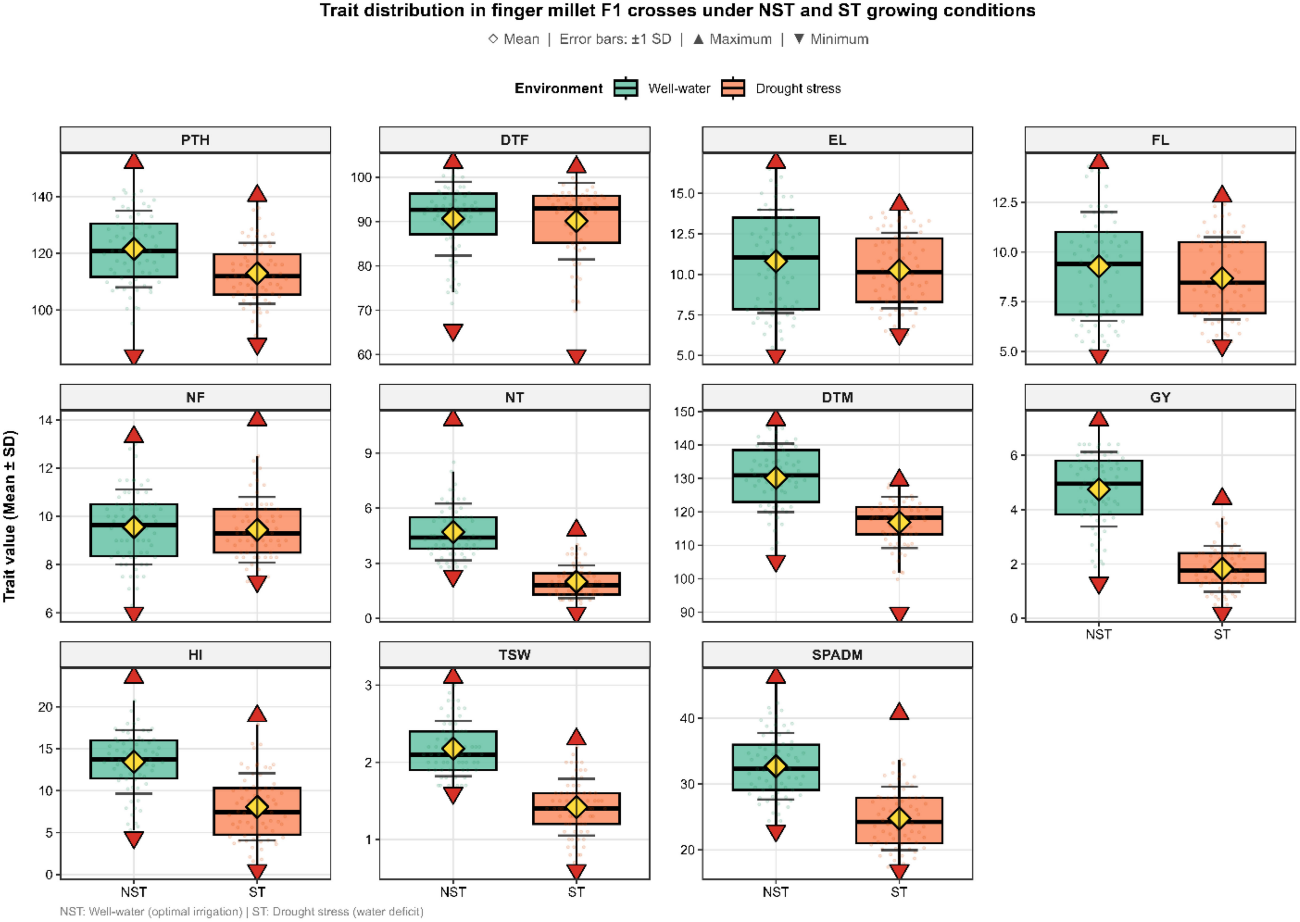
Distribution of agronomic and physiological traits in 70 finger millet F1 crosses under stress (ST) and non-stress (NST) growing conditions across glasshouse and field environments. Notes: PTH, plant height (cm); DTF, days to flowering; EL, ear length (cm); FL, length of the primary finger (cm); NT, number of productive tillers per plant; NF, the number of fingers on the primary ear; DTM, days to maturity; GY, grain yield (t/ha); HI, harvest index (%); TSW, thousand seed weight (g); SPADM, chlorophyll content at maturity.

The performance of finger millet parental lines, testers, and crosses revealed considerable variation for the assessed agronomic and physiological traits under ST and NST conditions across glasshouse and field environments (**Figures 2**-**4**; **Supplementary Tables 2–5**). Under ST conditions, PTH ranged from 98.3 cm for line G3 to 127.0 cm for line G1. Similarly, EL ranged from 6.8 cm (G4) to 13.0 cm (G5), whereas FL reached 12.3 cm (G5). Yield components also showed wide variation, with NT ranging from 1.0 (G7) to 3.0 (G2), and NF from 6.8 (G7) to 10.3 (G3). Phenological traits followed a similar trend, with DTF ranging from 75.8 (G3) to 98.5 days (G7) and with same lines, DTM ranging from 105.8 to 122.5 days. GY of lines was markedly reduced, averaging 2.1 t/ha under ST conditions compared with 4.9 t/ha under NST (~57% loss), although lines such as G3 (3.5 t/ha), G2 (2.6 t/ha), and G6 (2.6 t/ha) maintained relatively higher yields under ST conditions. Likewise, HI, TSW, and SPADM showed considerable variations among lines (**Figure 2**; **Supplementary Table 2**).

Under NST conditions, trait expression among lines was enhanced with broader ranges and higher mean values (**Figure 2**; **Supplementary Table 2**). PTH increased to a maximum of 141.0 for the line G6, whereas EL and FL reached 12.3 and 16.3 cm, respectively (G5). Yield components improved substantially, with NT peaking at 6.8 for lines G2 and G3 and NF at 12.3 (G5). DTF varied from 78.8 (G3) to 104.8 days (G5), whereas DTM extended up to 159.5 days (G5). Consequently, GY increased markedly, reaching up to 6.2 t/ha (G4). In parallel, HI (9.8%-18.6%), TSW (1.6-3.2 g), and SPADM (23.8–40.6) were at a significantly higher range than they were under ST conditions.

Under ST conditions, testers also showed marked differences across all traits (**Figure 3**; **Supplementary Table 2**). PTH varied from 83.8 for tester G13 to 124.3 cm (G10), whereas El and FL ranged between 6.8 (G11) to 14.5 cm (G12), and 5.5 (G11) to 12.3 cm (G12), respectively. NT and NF revealed reduced values under ST, with means of 1.8 and 8.8, respectively. DTF ranged from 67.8 (G17) to 100.5 days (G9), whereas DTM varied from 97.8 (G17) to 125.8 days (G10). GY remained low under ST, averaging 2.4 t/ha, although testers such as G8, G13, and G17 performed better and ranging from 2.4 to 2.9 t/ha. Furthermore, under ST conditions, the strong depressive effect of drought on productivity and physiological traits was observed across a wide range of variation in HI (2.7%–13.8%), TSW (0.9–1.7), and SPADM (19.2-32.6).

In contrast, under NST conditions, testers had improved performance and greater variability for assessed traits (**Figure 3**, **Supplementary Table 2**). PTH reached up to 134.3 cm G10 and G12, whereas EL and FL increased to 18.0 cm (G12) and 12.5 cm (G12), respectively. NT and NF also reached up to 4.8 (G15) and 10.5 (G10), respectively. GY increased substantially up to 7.0 t/ha (G8 & G11), accompanied by higher HI up to 17.8% (G11), TSW up to 2.7 g (G15), and SPADM up to 39.9 (G13).

Under ST conditions, F_1_ crosses revealed wide phenotypic variation and superiority over the parents for several traits (**Figure 4**; **Supplementary Tables 3** and **4**). PTH extended from 88.0 cm (G53) to 140.3 cm (G26), whereas EL varied from 6.3 cm (G47, G56, and G57) to 14.3 cm G18 and FL from 5.3 cm (G47) to 12.8 cm (G18). NT attained a minimum value of 0.3 (G76) and a maximum of 4.8 (G47), and NF spanning from 7.3 (G49, G51, G78, and G87) to 14.0 (G25). DTF ranged from 59.8 (G41) to 102.3 days (G78), and DTM varied from 89.8 (G41) to 129.5 days (G82). Despite an overall mean GY reduction to 1.8 t/ha (~62% loss), several crosses performed relatively well yielding under ST condition including 4.4 t/ha (G40), 3.7 (G38), 3.5 (G37, and G39), 3.4 (G83), 3.2 (G72), 2.8 (G41), 2.7 (G69), and 2.6 t/ha (G43, G46, and G70). Moreover, the top 15 performing crosses revealed reduced yield penalties (~51%) under ST conditions compared with NST conditions (**Supplementary Tables 3** and **4**). Wide variation among crosses was also noticed under ST conditions for traits, including HI, which ranged from 0.5 (G62) to 18.9% (G39), TSW from 0.6 (G63) to 2.3 g (G69), and SPADM from 16.9 (G49) to 40.7 (G47).

Under NST conditions, F_1_ crosses expressed high potential with substantial improvements across all traits (**Supplementary Tables 3**, **5**, and **Figure 4**). Notably, superior plant vigor was recorded in crosses such as G80 (PTH = 152.0 cm) followed by 151.0 (G87), 142.3 (G26), 142.0 (G27), 141.3 (G64), 140.8 (G75), whereas relatively short stature F_1_ crosses were noticed such as 84.0 cm (G39), 95.3 (G53), 100.5 (G38), 100.5 (G47), and 100.8 (G43). Similarly, EL displayed marked variation among crosses, with 16.9 cm (G24) excelling all others, closely followed by 16.8 (G32), and 16.5 (G18), whereas the lowest values were recorded in 5.0 cm (G47), followed by 5.5 cm (G38). Consistently, FL reflected similar trend, with the same superior cross G24 producing the longest fingers (14.5) followed by G32, G20 and G18, all at 14.3, whereas 5.0 cm (G47), and 5.5 cm (G38) exhibited the shortest FL. Yield components were markedly enhanced with G47 attaining the highest NT at 10.8 for cross G47 and NF at 13.3 for G44, whereas the lower values were observed in G69 (NT = 2.3) and G85 (NF = 6.0). Phenological variation was also evident, with early flowering and maturity observed in 65.5 days (G41) and 105.3 days (G39), respectively, whereas G81 remained relatively late (DTF = 103.3 days) and G54 (DTM = 147.5 days). Most importantly, GY peaked in 7.3 t/ha for G64, followed by other crosses exceeding 6.0 t/ha including 7.2 (G85), 6.4 (G80), 6.4 (G50), 6.4 (G83), 6.4 (G77), 6.4 (G79), 6.4 (G74), 6.3 (G41), 6.1 (G30), 6.1 (G56), 6.1 (G38), and 6.1 (G44). On the contrary, the lowest value of GY was recorded in cross 1.3 t/ha (G58), followed by G82 (1.9), and 2.0 (G65). The highest HI (%) was noted in 23.5% (G38) closely followed by 20.7 (G31), 19.5 (G44), and 18.5 (G46), whereas superior TSW (G77 = 3.1 g, G75 = 3.0 g, and G72 = 2.9 g), and SPADM (G23 = 46.2, G31 = 42.3, G29 = 41.6, and G79 = 41.2). Conversely, the lowest HI% was recorded by cross G58 at 4.4%, TSW (G51 and G60 both at 1.6 g), and SPADM (G49 = 22.9).

### 3.2 Combining ability variance for lines, testers, and lines × testers interactions across water regimes

Under ST conditions, variance component analysis revealed extensive and highly significant genetic variation among lines, testers, and their interactions for most agronomic and physiological traits (**Table 5**). General combining ability due to lines (GCA _L_) displayed highly significant variation across all evaluated traits, with particularly large mean squares observed for PTH, DTF, DTM, and SPADM. Equally, testers effect (GCA _T_) presented significant variation for most attributes, although EL and FL remained non-significant. In addition, interaction components demonstrated substantial responsiveness to water regimes (W). In this regard, GCA _L_ × W and GCA _T_ × W effects were significant for majority of traits, especially for PTH, DTF, GY, HI, TSW, and SPADM, whereas EL and FL consistently showed non-significant responses. Likewise, line × tester effects (SCA _L × T_) exhibited highly significant variation for most traits. Moreover, (SCA _L × T_) × W interactions remained significant across nearly all measured traits (**Table 5**).

**Table 5:** Partitioning of variance into general combining ability (lines and testers), specific combining ability, and their interaction effects across water regimes for agronomic and physiological traits in finger millet.

Sources of variation	Growing conditions	df	Traits										
			PTH	DTF	EL	FL	NT	NF	DTM	GY	HI	TSW	SPADM
			Mean squares										
GCA_L_	ST	6	1,657.1**	1,938.5**	135.1**	105.2**	9.5**	22.1**	1,341.3**	10.4**	298.8**	2.2**	450.9**
	NST	6	3,898.6**	1,439.1**	243.1**	192.6**	27.4**	36.8**	1,196.6**	16.9**	237.0**	2.1**	356.7**
GCA_T_	ST	9	548.0**	173.3**	11.25^NS^	9.2^NS^	2.7**	6.6**	235.9**	1.5**	35.2**	0.6**	54.3**
	NST	9	1,015.6**	173.4**	30.4**	22.3**	10.7**	4.1^NS^	329.4^NS^	6.8*	41.0**	0.4**	69.1**
GCA_L_ × W	ST	6	333.1**	434.6**	11.7^NS^	13.9^NS^	1.2^NS^	3.8^NS^	176.3*	5.0**	58.0**	0.1**	137.6**
	NST	6	335.4**	634.5**	4.0^NS^	3.3^NS^	1.2^NS^	3.1^NS^	923.9^NS^	6.3*	55.0**	0.7**	150.7**
GCA_T_ × W	ST	9	229.0*	94.4**	8.0^NS^	9.7^NS^	0.5^NS^	3.5^NS^	45.7^NS^	2.3**	52.7**	0.1*	47.9**
	NST	9	226.9**	55.0*	19.0**	13.1**	1.2^NS^	6.5^NS^	295.8^NS^	6.9**	31.2**	0.5**	37.3^NS^
SCA_L × T_	ST	54	314.8**	137.5**	10.8^NS^	8.7^NS^	2.6**	6.0**	112.6**	2.3**	42.5**	0.3**	59.8**
	NST	54	330.0**	163.7**	19.8**	13.2**	7.5**	7.5**	349.3^NS^	6.6**	40.2**	0.4**	79.0**
SCA_L × T_ × W	ST	54	196.6**	140.6**	5.9^NS^	3.9^NS^	1.1*	4.7**	136.0**	1.1**	26.1**	0.1**	40.5**
	NST	54	379.3**	129.4**	14.6**	8.9**	2.1^NS^	6.7**	266.8^NS^	6.3**	35.2**	0.4**	77.1**
Error	ST	139	112.2	23.8	8.4	6.5	0.7	2.5	62.2	0.2	4.9	0.1	14.6
	NST	139	71.4	26.7	5.9	4.5	2.1	3.8	267	2.7	10.8	0.1	23.6

ST and NST=drought-stressed and non-stressed growing conditions, respectively. ^NS^, *, and **= non-significant and significant at p<0.05 and 0.01, respectively; GCA_L_ and GCA_T_ are general combining abilities due to lines and testers, respectively; SCA_L × T_ is the specific combining ability due to lines × testers interaction; GCA_L_ × W and GCA_T_ × W denote lines and testers GCA interaction with water regimes, respectively; and SCA_L × T_ × W is the SCA × water regimes interaction. df, degree of freedom; PTH, plant height, cm; DTF, days to flowering; EL, ear length, cm; FL, length of the longest finger, cm; NT, number of productive basal tillers per main plant; NF, the number of fingers on the main ear; DTM, days to maturity; GY, grain yield, t/ha; HI, harvest index (%); TSW, thousand seed weight (g); SPADM, chlorophyll content, chlorophyll content at maturity.

Under NST conditions, the magnitude and significance of combining ability components remains prominent across the evaluated traits (**Table 5**). GCA_L_ effects continued to have highly significant difference for all traits, with markedly greater mean squares recorded for PTH, EL, and NT relative to the ST conditions. In parallel, GCA _T_ showed significant variation for most traits, except NF and DTM. Interaction analysis further revealed that GCA _L_ × W effects were significant for key traits including PTH, DTF, GY, HI, TSW, and SPADM. Parallelly, GCA _T_ × W effects remained significant for most traits but showed limited response for NT, NF, DTM, and SPADM. In addition, SCA _L × T_ effects were highly significant almost for all traits, except DTM. Correspondingly, (SCA _L × T_) × W interaction also displayed significant variation for the majority of traits, particularly for PTH, DTF, EL, FL, GY, HI, TSW, and SPADM (**Table 5**).

### 3.3 Combining ability effects of finger millet genotypes

#### 3.3.1 General combining ability effects

Under ST conditions, substantial variation in GCA effects was measured among the lines for all assessed traits (**Table 6**). For PTH, G1 (7.19), G6 (6.10), and G7 (5.20) displayed positive effects, whereas G4 recorded the largest negative value (−10.79*). Advantageous negative GCA effects for earliness were exhibited by G3 for both DTF (−11.91**) and DTM (−7.99**). In contrast, G5 (3.39) and G6 (3.43) displayed relatively high positive values for DTF, whereas G7 recorded the highest positive effect for DTM (4.24*). Positive and significant effects for EL and FL were mainly contributed by G1 (2.17**, 1.28*) and G2 (1.72*, 1.71*), respectively. For NT and NF, G3 displayed positive effects of 0.29 and 0.53, whereas G1 exhibited the highest NF value (0.9*). Regarding productivity-related traits, G3 consistently recorded the highest positive GCA effects for GY (1.33**), HI (5.74**), and TSW (0.31*). Similarly, positive SPADM values were observed for G3 (6.30*), G7 (2.54), G2 (1.30), and G1 (0.91), whereas G5 (−4.70*) and G6 (−3.34) showed negative effects.

**Table 6:** General combining ability effects for agronomic and physiological traits of 7 lines and 10 testers of finger millets under stressed (ST) and non-stressed (NST) conditions across glasshouse and field environments.

Parental code	Traits																					
	PTH		DTF		EL		FL		NT		NF		DTM		GY		HI		TSW		SPADM	
	Growing conditions																					
	ST	NST	ST	NST	ST	NST	ST	NST	ST	NST	ST	NST	ST	NST	ST	NST	ST	NST	ST	NST	ST	NST
Lines																						
G1	7.19	7.59	0.57	0.36	2.17**	3.01**	1.28*	2.84**	0.36	−0.06	0.90*	0.83	0.37	0.41	−0.27	−0.12	−1.32	−0.59	−0.21	−0.29	0.91	−1.62
G2	−2.59	−3.15	0.41	−0.78	1.72*	1.77*	1.71*	1.81*	0.29	0.69	−0.1	−0.24	−2.7	0.01	0.21	0.17	1.8	1.55	0	−0.04	1.3	2.61*
G3	−5.19	−11.12*	−11.91**	−17.23**	−1.34	−2.12*	−1.23	−1.88*	0.29	1.00*	0.53	0.97*	−7.99**	−16.34*	1.33**	0.22	5.74**	2.04	0.31*	0.09	6.30*	0.91
G4	−10.79*	−11.16*	2.17	3.97	−2.17**	−2.99**	−2.05**	−2.77**	0.01	−0.16	−0.67	−0.86	0.9	1.87	−0.01	0.03	0.72	1.31	−0.14	−0.21	−3.01	−1.33
G5	0.09	−0.22	3.39	8.99*	0.78	1.89*	1.76*	1.25	−0.2	−0.52	−0.03	0.55	2.86	8.57*	−0.96*	−0.38	−4.64*	−3.38*	−0.24*	−0.19	−4.70*	−1.66
G6	6.1	7.18	3.43	5.86	−1.11	−0.69	−0.96	−0.38	−0.63*	−0.73	−0.23	−0.42	2.32	4.49	−0.12	0.11	−1.36	−0.69	0.17	0.52**	−3.34	−0.4
G7	5.2	10.88*	1.94	−1.18	−0.06	−0.87	−0.51	−0.88	−0.13	−0.22	−0.4	−0.82	4.24*	0.99	−0.19	−0.03	−0.94	−0.23	0.11	0.12	2.54	1.49
SE_L_	2.93	3.11	2.92	3.13	0.58	0.71	0.6	0.58	0.22	0.38	0.33	0.38	2.08	2.79	0.25	0.32	1.18	1.06	0.09	0.14	1.64	1.08
Testers																						
G8	2.1	3.01	-0.29	0.04	0.36	0.25	0.18	0.23	−0.24	0.04	−0.06	−0.26	−1.15	0.18	−0.02	0.14	−0.07	0.3	−0.02	−0.01	0.05	0.46
G9	−2.71	−2.12	0.51	0.05	0.05	−0.12	0.09	−0.03	−0.16	−0.25	0.09	−0.07	1.32	−0.18	−0.03	0.02	0.1	0.11	0.06	0	−0.44	−0.13
G10	−0.05	−4.9	0.16	0	−0.31	0	-0.16	−0.08	−0.08	−0.29	−0.04	0.19	0.73	−0.22	0.04	−0.06	0.32	0.11	0.05	0.02	−0.78	−0.1
G11	1.51	1.42	−0.75	−0.08	0.29	0	0.15	0.05	−0.33	−0.06	−0.05	−0.05	−0.86	−0.17	0.01	−0.01	−0.19	−0.16	−0.08	−0.02	−0.46	0.2
G12	1.08	3.28	−0.01	−0.03	0.32	0.33	0.26	0.29	0.08	0.17	0.03	0.06	1.3	0.39	−0.13	−0.19	−1.23	−0.56	0.06	0.01	−0.56	0.59
G13	−7.36	−8.16*	−0.77	−0.12	−0.23	−0.17	−0.16	−0.25	−0.16	0.03	0.05	0.13	−2.32	−0.1	0.12	−0.11	1.6	−0.05	−0.08	−0.03	0.97	0.87
G14	6.01	4.28	−0.03	−0.05	0.16	0	−0.02	0.08	−0.08	0.13	−0.05	−0.36	−0.03	−0.12	−0.03	0.22	−0.58	0.47	0.08	−0.01	0.15	−0.51
G15	−0.29	2.13	0.25	0.04	−0.18	−0.05	−0.2	0	0.16	−0.18	0.03	0.04	−0.67	0.46	−0.01	−0.07	−0.36	−0.43	0.06	0.08	−0.09	−0.4
G16	−1.15	0.03	0.06	0.02	−0.24	−0.05	−0.03	−0.07	0.08	0.13	0	−0.22	−0.81	−0.18	0.02	0	0.12	0.03	−0.09	−0.05	−0.21	−0.11
G17	0.85	1.05	0.88	0.14	−0.22	−0.2	−0.1	−0.22	0.08	0.29	0	−0.11	2.48	−0.06	0.04	0.05	0.3	0.18	−0.04	0	1.37	0.42
SE_T_	2.18	3.06	1.02	0	0.58	0.39	0.6	0.36	0.06	0.29	0.15	0	1.6	1.18	0.16	0	1.07	0	0.08	0.07	1.17	0.01

Growing conditions, ST: stress, and NST: non-stress growing conditions. * and ** denote significant at 5% and 1% levels of significance, respectively. SE_L_ and SE_T_ are standard error of lines and testers, respectively. PTH, plant height (cm); DTF, days to flowering; EL, ear length (cm); FL, length of the primary finger (cm); NT, number of productive tillers per plant; NF, the number of fingers on the primary ear; DTM, days to maturity; GY, grain yield (t/ha); HI, harvest index (%); TSW, thousand seed weight (g); SPADM, chlorophyll content at maturity.

Under NST conditions, positive effects for PTH were highest in G7 (10.88*) followed by G1 (7.59) and G6 (7.18), whereas G4 (−11.16*) and G3 (−11.12*) showed strong negative values. Valuable negative GCA effects for DTF and DTM were again pronounced in G3, with values of −17.23** and −16.34*, respectively. Conversely, G5 recorded the largest positive effect for DTF (8.99*) and DTM (8.57*). Significant positive GCA effects for EL and FL were detected in G1 (3.01**, 2.84**) and G2 (1.77*, 1.81*), whereas G4 exhibited strong negative values for both traits. For NT and NF, G3 maintained the highest positive effects of 1.00* and 0.97*, respectively. Moreover, positive GCA effects for GY and HI were mainly associated with G3 (0.22 and 2.04), G2 (0.17 and 1.55), and G4 (HI = 1.31). For TSW, G6 recorded the highest positive effects (0.52**), followed by G7 (0.12) and G3 (0.09). In addition, SPADM values were highest in G2 (2.61*), followed by G7 (1.49) and G3 (0.91) (**Table 6**).

Among the testers evaluated under ST conditions, positive GCA effects for PTH were highest in G14 (6.01), followed by G8 (2.10) and G11 (1.51), whereas G13 recorded the largest negative effect (−7.36). Desirable negative GCA effects for both DTF and DTM were observed in testers G11 (−0.75 and −0.86), G13 (−0.77 and −2.33), and G8 (−0.29 and −1.15), respectively. Conversely, positive GCA effects for both DTF and DTM were recorded in G17 (0.88 and 2.48) and G9 (0.51 and 1.32), respectively. For EL and FL, positive effects were generally low but were relatively higher in G8 (0.36 and 0.18), G12 (0.32 and 0.26), and G11 (0.29 and 0.15). Similarly, positive effects for NT and NF were recorded in G15 (0.16 and 0.03) and G12 (0.08 and 0.03) in the same order. In terms of yield-related traits, positive GCA effects for GY were observed in G13 (0.12), G10, and G17 (0.04 each), whereas HI values were highest in G13 (1.60) followed by G10 (0.32) and G17 (0.30). Similarly, TSW showed positive effects in G14 (0.08), G9, G12, and G15 (0.06, each). For SPADM, positive GCA values were mainly recorded in G17 (1.37), G13 (0.97), G14 (0.15), and G8 (0.05) (**Table 6**).

Under NST conditions, positive PTH effects were prominent in G14 (4.28), G12 (3.28), G8 (3.01), and G15 (2.13), whereas G13 showed the strongest negative value (−8.16*). Desirable negative GCA effects for both DTF and DTM were observed in testers G13 (DTF: −0.12 and DTM: −0.10) and G14 (DTF: −0.05 and DTM: −0.12). In contrast, positive GCA effects for both DTF and DTM were relatively higher in G15 (DTF: 0.04 and DTM: 0.46) and G8 (DTF: 0.04 and DTM: 0.18). For EL and FL, positive values were observed in G8 (0.25 and 0.23), G12 (0.33 and 0.29), and G14 (0.00 and 0.08). Equally, positive NT and NF effects were observed in G12 (0.17 and 0.06) and G13 (0.03 and 0.13). Yield-related traits displayed positive GCA effects in several testers, particularly for GY in G14 (0.22), G8 (0.14), and G17 (0.05). Likewise, HI values were relatively higher in G14 (0.47), G8 (0.30), and G17 (0.18), whereas positive TSW effects were recorded in G15 (0.08). G10 (0.02), and G12 (0.01). For SPADM, positive effects were highest in G13 (0.87) followed by G12 (0.59), G8 (0.46), G17 (0.42), and G11 (0.20) (**Table 6**).

#### 3.3.2 Specific combining ability effects of the crosses

Under ST conditions, several crosses exhibited strong SCA effects across agronomic and physiological traits in both desirable and undesirable direction (**Table 7**). Desirable SCA effects for PTH were pronounced in G29, G31, G32, G37, G59, G61, G70, and G75 varying from −0.5 in G29 to 0.5 in G31. Desirable negative SCA effects for both DTF and DTM were G41, G84, G72, G19, G53, G23, and G58. Among these, G41 recorded the highest negative SCA effects for DTF (−29.1**) and DTM (−16.8**), succeeded by G84 at −23.3** and −10.3, respectively, whereas G72 showed −22.1** for DTF and −8.9 for DTM. Conversely, strong positive SCA effects for DTF and DTM were noticed in G81 (15.5** and 7.6), G26 (14.8** and 10.7*), G78 (14.5** and 6.7). Ear traits also varied markedly, with positive SCA effects for EL and FL recorded in G41 (2.3* and 2.3**) and G18 at 0.9 for EL and G62 at 0.9 for FL, whereas negative effects were evident in G23 (−1.7 and −1.8*) and G40 (−1.0 and −1.0). Moreover, positive SCA effects for NT were recorded in G40 (2.3**), G81 (1.0), and G61 (0.9), whereas negative SCA effects were noticed in G22 (−1.8), G39 (−1.0), G41 (−0.9), and G64 and G65 (−0.8 each). NF was highest in G25 and G82 (2.5** each), followed by G18 (1.9**) and G66 (1.7**), whereas the high negative SCA values were recorded in G26 (−1.8**), G51 (−1.6), and G40, G85, and G49 (−1.5 each). GY SCA effects were markedly high in G79 (1.4**), G70 (1.3**), G38 (1.1**), and G37, G32, G63, G41, G39, G54, G83, and G64 (0.7-0.9**), whereas significant negative effects were recorded in G86, G76, and G81 (−0.9**; all) and G58, G42, G67, G61, G25, and G62 (−0.7** all). Likewise, the HI ranged from 9.5** in G38 and 8.1** in G70 to −4.4** in G76 and −4.4** in G42. Positive TSW effects were observed in G72, G46, and G58 all at 0.5**, and G37 (0.4**), whereas negative effects occurred in G35, G68, and G63 all at −0.5**. SPADM also displayed wide variation, ranging from 15.7** in G47 and 12.9** in G23 to −8.2** in G19 and −7.9* in G41 (**Table 7**).

**Table 7:** Specific combining ability effects for agronomic and physiological traits of 70 finger millet crosses under stressed and non-stressed conditions in greenhouse and field environments.

Codes of crosses	Traits																					
	PTH		DTF		EL		FL		NT		NF		DTM		GY		HI		TSW		SPADM	
	Growing conditions																					
	ST	NST	ST	NST	ST	NST	ST	NST	ST	NST	ST	NST	ST	NST	ST	NST	ST	NST	ST	NST	ST	NST
G18	2.5	−3.2	3.6	3.5	0.9	1.9	0.8	0.8	−0.2	−0.3	1.9*	0.7	3.1	3.3	−0.2	0.0	−1.0	−0.1	0.2	0.0	1.5	0.2
G19	0.7	1.4	−20.1**	−16.5**	0.0	−1.5	−0.2	−0.3	0.5	0.8	−0.5	0.3	−9.1*	−15.5**	−0.3	−0.1	−1.9	−0.2	−0.1	−0.3	−8.2**	0.1
G20	−11.3	−8.5	−10.9**	−1.1	0.3	2.7	0.5	0.8	0.3	0.4	−0.9	0.3	−4.6	−3.1	0.0	0.0	−0.3	−0.3	−0.2	0.0	3.7	0.1
G21	3.5	9.2	4.6	0.3	0.1	−1.4	0.3	0.4	0.2	−0.9	−0.5	−0.6	4.2	5.9	−0.2	0.0	−1.2	0.0	0.2	−0.5**	−1.2	−0.2
G22	−1.9	−3.9	7.0*	0.3	0.4	−0.8	0.2	−0.9	−1.8*	−2.4*	1.6	0.1	3.7	−2.0	0.1	−0.1	2.0	−0.3	−0.3*	−0.1	−2.2	−0.5
G23	−4.0	−7.3	−18.6**	−21.1**	−1.7	−5.4**	−1.8*	−2.4*	0.5	0.8	1.0	−0.1	−8.2	−8.4	−0.3	0.0	−0.2	3.3**	0.0	−0.2	12.9**	1.0**
G24	11.3	15.2*	−1.7	−3.0	0.3	3.9*	0.5	0.8	0.1	0.7	−1.2	0.5	0.0	−4.6	−0.2	0.0	0.1	0.0	0.1	0.1	−2.2	0.1
G25	−11.7	−24.5**	10.9**	10.1*	0.6	2.9	0.1	0.7	−0.6	0.5	2.5**	0.7	−6.8	5.9	−0.7**	−1.2**	−2.5*	−2.8*	0.2	0.3	0.0	−0.2
G26	14.5*	18.1*	14.8**	15.6**	−0.7	1.6	−0.6	0.5	0.6	0.2	−1.8	0.3	10.7*	11.1*	0.5*	0.0	2.6*	3.5**	−0.3*	−0.2	−1.5	−0.2
G27	5.9	15.0*	11.4**	12.4**	0.4	−0.1	0.6	0.2	−0.1	0.4	0.1	−0.6	11.3*	7.8	−0.4*	0.0	−1.9	−0.2	−0.1	−0.2	−1.4	−0.3
G28	−10.2	−5.2	3.4	−0.1	−0.3	0.6	−0.1	0.4	0.0	0.0	−0.2	1.4	−4.0	2.8	−0.3	−1.1**	−1.1	−3.8**	0.1	−0.2	−1.5	0.0
G29	−0.5	−1.1	−3.3	−3.3	−0.2	1.0	0.0	0.0	0.2	0.4	−0.5	0.3	1.4	8.8	−0.1	0.0	0.0	0.3	0.2	−0.1	2.8	0.6
G30	7.2	−0.2	−1.9	−3.3	0.3	−0.3	0.2	0.4	−0.1	−0.2	1.0	−0.1	2.2	−5.4	−0.3	0.1	−1.4	0.1	0.1	0.2	−2.2	−0.3
G31	0.5	0.0	−5.3	−4.7	0.1	−0.3	−0.1	−0.2	−0.2	1.0	0.1	−0.6	0.3	2.8	0.0	2.3**	1.0	6.7**	−0.1	−0.1	−1.3	0.6
G32	0.2	−0.5	−8.7*	−3.8	0.1	3.1	−0.2	1.0	0.4	−0.3	−0.5	0.3	−1.7	−5.8	0.7**	1.7**	4.0**	5.4**	−0.1	−0.3	5.2	−0.2
G33	−1.8	−4.4	9.7**	10.7**	0.8	−0.2	0.4	−0.3	0.0	−0.4	1.0	0.3	−5.7	5.8	−0.3	−0.1	−1.4	−0.4	−0.1	-0.2	−0.1	0.1
G34	5.7	2.7	2.5	−0.1	−0.1	−0.7	0.0	−0.4	0.2	−0.2	−0.5	−0.8	4.8	−3.6	−0.5*	0.0	−2.2	−0.2	0.0	0.5**	0.8	0.4
G35	−1.8	3.3	2.5	3.2	0.0	−1.4	0.2	−0.2	0.2	−0.2	−0.8	0.5	4.8	−0.2	−0.6**	0.0	−3.0*	−0.3	−0.5**	−0.1	−5.0	−0.2
G36	-3.1	−1.0	4.9	3.2	0.0	−0.1	0.2	−0.2	0.1	0.0	0.7	−0.3	−3.4	−0.2	0.1	0.0	2.9*	0.4	−0.2	−0.1	0.1	−0.3
G37	0.4	−7.8	0.5	−2.8	0.0	0.4	0.1	0.0	−0.3	−0.8	0.1	−1.7	3.7	−5.1	0.7**	0.0	1.8	−0.1	0.4**	−0.4*	3.1	−0.2
G38	−1.5	−1.7	−6.8	−4.7	−0.3	−2.7	−0.3	−0.8	−0.7	−0.3	1.5	0.9	−3.8	−6.7	1.1**	1.5**	9.5**	7.2**	0.2	−0.2	6.2*	0.5
G39	−11.1	−28.8**	−5.8	−3.8	−0.8	−1.6	−0.7	−0.3	−1.0	−0.7	0.6	−0.4	−3.2	−5.9	0.8**	0.0	7.9**	0.0	−0.1	−0.1	1.6	0.3
G40	−2.5	0.0	3.4	−5.7	−1.0	−0.9	−1.0	−0.7	2.3**	2.3*	−1.5	−0.2	2.1	2.7	0.1	0.0	−0.6	−0.1	−0.1	0.0	−0.8	0.3
G41	11.2	19.4**	−29.1**	−32.2**	2.3*	2.0	2.3**	2.3*	−0.9	0.7	0.9	−0.9	−16.8**	−19.4**	0.7**	0.1	0.0	−0.3	−0.3*	0.0	−7.9*	−0.7
G42	−3.7	9.3	4.3	8.3*	−0.7	2.9	−0.9	0.7	−0.4	−0.3	0.6	1.6	2.7	3.8	−0.7**	−1.4	−4.4**	−5.2	0.2	−0.2	−6.7*	−0.4
G43	1.7	−4.2	−4.4	−9.4*	−0.5	−0.4	−0.4	−0.3	0.0	−0.5	−0.3	0.3	−2.4	0.5	−0.2	0.0	−1.1	0.3	0.1	−0.2	4.2	0.3
G44	−2.5	−7.1	11.6**	8.3*	−0.1	−0.4	0.0	−0.5	0.5	0.0	0.9	0.5	5.2	3.8	−0.2	0.0	0.0	0.5	0.2	0.6**	1.0	0.4
G45	−0.9	−4.0	−3.4	−6.6	0.5	1.2	0.5	0.0	0.5	−0.1	−0.9	−0.2	−1.8	−8.2	−0.1	0.0	−1.0	−0.3	0.1	0.0	−1.5	0.0
G46	3.3	10.4	−4.9	−5.2	0.7	−0.5	0.5	−0.1	−0.5	−0.8	0.3	−0.6	−2.7	−7.0	0.0	0.0	0.8	0.4	0.5**	0.6**	−2.2	−0.2
G47	−0.7	−8.0	12.1**	29.2**	−0.5	−2.2	−0.5	−0.8	0.4	0.1	0.6	1.1	4.6	20.7**	−0.2	0.0	−2.2	−0.2	−0.3	−0.5**	15.7**	0.1
G48	4.5	8.6	3.4	3.0	0.4	0.7	0.4	0.1	−0.4	−0.2	0.5	0.1	5.9	1.8	0.9**	0.0	5.7**	0.3	0.3	−0.1	−0.1	−0.2
G49	3.6	−6.0	4.8	2.5	−0.3	−0.7	−0.4	−0.2	0.6	−0.3	−1.3	−0.3	−0.3	1.4	0.6**	0.9*	0.7	0.1	0.1	−0.4*	−6.5*	−0.6
G50	−2.8	−5.5	−3.4	−0.3	0.8	−0.4	0.6	−0.3	−0.3	−0.4	1.1	−0.6	2.2	−0.8	0.1	0.1	0.5	2.9*	−0.1	−0.5**	−1.2	0.3
G51	−11.1	0.6	5.8	10.0*	−0.4	−0.3	−0.3	−0.4	0.1	0.6	−1.6	−0.1	−1.7	11.2*	−0.3	−1.6**	−1.7	−4.4**	−0.1	−0.3	7.4*	0.4
G52	−2.2	−4.1	2.9	4.4	0.3	2.1	0.1	0.6	−0.1	0.1	−0.1	−0.1	−0.3	2.9	0.9**	0.0	3.6**	−0.1	0.1	0.3	−3.1	−0.1
G53	−7.4	−11.3	−19.9**	−19.4**	0.0	−0.7	−0.1	0.1	−0.3	0.0	0.2	1.0	−5.7	−16.2**	−0.5*	−0.1	−2.0	−0.7	−0.3	0.2	−4.8	−0.2
G54	−2.2	−2.5	4.8	1.6	−0.3	−2.0	−0.3	0.0	−0.3	−0.3	−0.7	−1.5	−2.6	0.7	0.8**	0.0	4.0**	4.8**	−0.2	−0.5**	−4.0	0.0
G55	−1.7	−2.8	5.8	2.0	−0.4	−2.0	−0.3	−0.3	−0.5	−0.3	−1.0	−1.0	−5.7	1.0	−0.4	1.0*	−2.8*	0.0	0.3	0.4*	−1.3	−0.3
G56	1.1	−0.4	−4.4	−4.0	−0.5	−1.3	−0.5	−0.3	0.0	−0.2	−0.1	−0.6	1.7	−3.8	−0.5*	2.0**	−2.8*	3.4**	−0.3	−0.2	3.4	0.3
G57	3.9	0.2	5.3	5.3	−0.5	0.2	0.0	−0.2	−0.1	−0.3	−0.1	0.3	5.4	3.7	−0.5*	0.0	−2.2	0.2	−0.1	0.3	5.6	−0.1
G58	−8.0	0.2	−15.1**	5.3	−0.2	−3.0	−0.1	−0.3	−0.7	−0.3	0.4	−1.6	−9.5*	7.7	−0.7**	−2.6**	−2.6*	−5.8**	0.5*	0.5**	−4.3	−0.4
G59	0.2	−7.6	11.5**	8.5*	−0.7	0.7	−0.7	−0.3	0.7	0.3	−0.8	1.3	6.3	5.8	−0.3	−0.1	−1.1	−0.6	0.3	−0.1	−2.9	−0.4
G60	2.8	4.9	3.3	−0.8	0.9	0.2	0.7	0.3	0.5	0.4	0.1	−0.2	1.2	−2.5	−0.4*	−0.1	−1.7	−0.3	−0.4*	−0.5**	0.0	−0.1
G61	−0.4	−9.0	2.8	7.6	0.4	2.2	0.5	0.4	0.9	0.3	−0.8	3.1**	1.5	2.4	−0.7**	−1.1*	−2.6*	−2.9*	0.2	0.5**	1.5	0.7
G62	7.0	3.0	−13.2**	−4.0	0.7	0.8	0.9	0.3	0.7	0.1	−0.5	−0.2	7.4	7.7	−0.7**	−1.1*	−2.6	−3.0*	−0.2	0.2	−4.3	−0.3
G63	−3.0	−1.0	1.3	−3.6	0.6	−0.7	0.7	0.1	0.2	−0.6	−0.2	−0.2	2.9	−4.7	0.7**	1.4**	3.8**	4.3**	−0.5**	0.6**	3.1	0.0
G64	5.0	14.0*	2.8	−3.6	0.5	−2.9	0.2	−0.6	−0.8	0.6	−0.5	−0.9	3.8	−4.7	0.8**	1.3**	1.9	3.2*	0.0	−0.3	−0.8	0.0
G65	1.8	11.0	−0.1	−8.2*	−0.8	4.7*	−0.8	0.6	−0.8	0.1	−0.2	0.6	−3.6	−8.5	0.4*	−2.7**	0.4	−6.0**	0.1	−0.4*	−1.2	−0.2
G66	−7.8	−11.6	6.2	8.5*	−0.7	1.7	−0.8	0.1	0.1	−0.3	1.7*	1.1	−8.4	5.8	−0.6**	0.0	−2.0	0.2	−0.3	0.4*	1.7	0.0
G67	2.6	−3.9	6.2	1.6	−0.1	−0.9	0.1	−0.3	−0.2	0.1	0.1	−0.5	2.9	−0.6	−0.7**	−1.0*	−2.1	0.1	−0.1	−0.1	0.0	0.2
G68	6.0	0.6	1.6	2.6	0.1	−0.8	−0.2	0.1	0.3	−0.5	1.0	−0.5	7.0	1.0	−0.3	0.0	−1.4	0.0	−0.5**	0.2	−5.4	−0.6
G69	8.2	11.4	−0.4	7.3	0.4	−2.7	0.3	−0.5	0.4	0.0	−0.9	−1.2	2.1	4.7	0.0	0.1	−1.1	0.2	0.1	−0.2	−4.4	−0.5
G70	−0.1	−3.5	2.5	−0.2	0.3	−1.1	0.4	0.0	−0.7	0.2	−0.6	−0.1	3.8	−1.3	1.3**	1.4**	8.1**	4.3**	−0.1	−0.1	−1.2	−0.2
G71	1.8	6.2	3.0	−1.6	−1.0	5.5**	−0.7	0.2	0.1	0.4	−1.2	1.0	−4.1	−2.4	−0.1	0.0	−0.7	0.3	0.2	0.2	−0.2	−0.3
G72	5.3	13.2	−22.1**	−21.1**	0.0	0.4	0.1	0.4	−0.1	0.9	0.1	2.4*	−8.9	−8.4	0.9**	0.0	2.5*	0.1	0.5**	0.4*	2.8	0.4
G73	1.8	12.2	−0.4	1.7	−0.2	1.8	−0.1	0.9	−0.1	0.1	−0.6	−0.1	2.1	0.2	−0.6**	−0.1	−3.6**	−0.7	0.0	−0.1	−6.1	−0.1
G74	−1.0	−4.0	4.0	4.0	0.2	−1.6	−0.1	0.1	−0.3	−0.6	1.0	−0.5	0.4	2.1	0.4*	0.1	2.8*	6.1**	0.2	0.0	3.6	0.5
G75	0.3	12.1	4.5	4.9	−0.4	−2.8	−0.3	-0.6	0.2	−0.1	1.0	−0.5	3.3	2.8	0.6**	−0.1	2.6*	−6.0**	−0.2	0.0	6.3*	0.6
G76	−3.7	−14.2*	8.8*	7.3	0.3	1.3	0.2	−0.1	0.2	−0.5	0.7	−1.2	−0.8	4.7	−0.9**	−1.4**	−4.4**	−3.7**	0.1	0.2	−0.6	−0.1
G77	−10.6	−12.8	5.0	2.6	0.0	−0.6	0.2	−0.5	−0.4	−0.6	−0.3	−0.5	0.0	1.0	−0.4	0.1	−2.5*	0.3	−0.1	0.4*	0.1	−0.4
G78	−2.3	−4.6	14.5**	2.6	−0.4	−1.7	−0.4	−0.6	0.3	0.9	−0.6	−0.2	6.7	3.2	−0.6**	0.0	−2.3	0.2	−0.1	−0.2	−3.1	−0.6
G79	−1.3	−0.1	3.4	0.8	0.1	4.1*	0.3	0.9	−0.1	−0.1	0.3	0.7	2.7	−3.2	1.4**	2.1**	4.4	3.4**	−0.2	0.4*	5.6	0.5
G80	11.9	32.8**	−13.1**	−10.4*	−0.1	0.1	−0.1	−0.1	0.2	−1.0	−0.9	0.0	−4.3	−2.0	0.2	2.3**	−0.7	0.1	0.0	0.0	−5.4	−0.4
G81	1.5	4.3	15.5**	18.0**	0.4	−4.2*	0.2	−1.0	1.0	0.3	−0.3	0.0	7.6	13.0**	−0.9**	−2.3**	−3.6	−6.0**	0.0	−0.2	2.6	0.0
G82	−1.1	−6.5	7.7*	6.4	0.7	1.7	1.0	0.3	−0.4	−0.1	2.5**	0.2	5.0	1.3	0.1	−3.4**	0.3	−9.8**	0.2	0.2	−0.3	0.4
G83	−11.7	−27.5**	6.8	3.6	−0.3	1.2	−0.4	−0.1	0.5	1.2	−0.6	0.2	−5.7	−0.9	0.8**	0.1	2.8*	0.3	0.0	0.3	5.6	0.3
G84	3.5	1.6	−23.3**	−19.2**	0.4	4.0*	0.5	1.2	−0.3	−0.2	1.0	1.5	−10.3	−9.5*	0.9**	2.0**	1.4	5.0**	0.3	0.0	3.8	0.1
G85	13.2*	14.3*	3.8	5.0	−0.5	−2.4	−0.3	−0.2	−0.3	−0.5	−1.5	−1.8	3.9	0.2	0.2	2.4**	−0.5	0.3	0.2	0.3	1.3	0.4
G86	−8.1	−20.7**	−9.7**	−5.3	−0.3	−3.0	−0.3	−0.5	−0.6	−0.2	−0.9	−0.2	−3.2	−8.0	−0.9**	−1.4**	−3.6**	−3.8**	−0.4*	0.4*	−4.2	−0.1
G87	1.3	25.9**	−5.4	−2.9	−0.4	−0.7	−0.6	−0.2	0.8	0.9	−0.9	−2.0	−1.5	7.0	−0.1	0.0	−0.4	0.0	0.2	0.0	−2.1	0.0
Standard error of crosses	6.1	6.9	3.5	2.9	0.9	1.8	0.8	0.9	0.0	0.0	0.8	1.1	5.1	4.6	0.1	0.3	1.2	1.2	0.1	0.2	3.0	1.2

Growing conditions, ST: stress and NST: non-stress growing conditions. * and ** denote significant at 5% and 1% level of significance, respectively. PTH, plant height (cm); DTF, days to flowering; EL, ear length (cm); FL, length of the primary finger (cm); NT, number of productive tillers per plant; NF, the number of fingers on the primary ear; DTM, days to maturity; GY, grain yield (t/ha); HI, harvest index (%); TSW, thousand seed weight (g); SPADM, chlorophyll content at maturity.

Under NST conditions, substantial SCA effects were similarly observed in both directions across all traits (**Table 7**). Desirable SCA effects for PTH were recorded in G68, G51, G57, G58, G40, G31, G79, G30, G56, and G32 varying from −0.5 in G32 to 0.6 in G68. Furthermore, beneficial negative effects for both DTF and DTM were most evident in G41, G23, G72, G53, G84, and G19. In this regard, G41 recorded the strongest negative effects for DTF (−32.2**) and DTM (−19.4**), whereas G23 and G72 each expressed identical SCA effects of −21.1** for DTF and −8.4 for DTM. In contrast, strong positive SCA effects for DTF and DTM were detected in G47 (29.2** and 20.7**), G81 (18.0** and 13.0**), G26 (15.6** and 11.1**), and G51 (10.0** and 11.2**). Positive SCA effects for EL and FL were notable in G71 (5.5** and 0.2), G65 (4.7* and 0.6), G79 (4.1* and 0.9), G84 (4.0* and 1.2), and G41 (2.0* and 2.3*), in the same order, whereas negative effects were observed in several crosses including G23 (−5.4** and −2.4*), G81 (−4.2* and −1.0), G86 (−3.0* and −0.5), and G58 (−3.0 and −0.3), in that order. NT and NF also varied considerably, with G40 showing the highest positive NT effect (2.3*) and G61 the highest NF effect (3.1**), whereas negative values were recorded in G22 (−2.4*) and G87 (−2.0), respectively. For GY, the highest positive SCA effects were observed in G85 (2.4**), G80 and G31 (2.3** each), G79 (2.1**), and G84 and G56 (2.0** both), whereas negative effects occurred in G82 (−3.4**), G65 (−2.7**), G58 (−2.6**), and G81 (−2.3*). Similarly, HI ranged from 7.2** in G38, 6.7** in G31, 6.1** in G74 to −9.8** in G82, and −6.0** in G65, G81, and G75. TSW also displayed contrasting effects, varying from 0.6** in G63, G44, and G46 to −0.5** in G60, G47, G21, G50, and G54. SPADM further ranged from 1.0** in G23 and 0.7 in G61 to −0.7 in G41 (**Table 7**).

### 3.4 Gene action, degree of dominance, heritability and contribution to total variance

Estimates of genetic variance components revealed substantial differences in the magnitude of additive and dominance effects across water regimes and traits (**Table 8**). Under ST conditions, additive variance (δ^2A^) ranged from 0.06 for TSW to 40.60 for DTF, whereas dominance variance (δ^2D^) varied from 0.05 for TSW to 29.55 for PTH. Relatively larger additive components were recorded for DTF, PTH, EL, FL, NF, DTM, HI, TSW, and SPADM, whereas NT and GY exhibited comparatively greater dominance effects. In contrast, under NST conditions, δ^2A^ increased markedly for several traits, extending from 0.03 for TSW to 120.24 for PTH, whereas δ^2D^ ranged from nearly zero for TSW to 20.62 for DTM. Moreover, additive effects predominated for PTH, DTF, EL, FL, NF, GY, HI, TSW, and SPADM, whereas NT and DTM retained relatively stronger dominance components.

**Table 8:** Estimates of additive (**δ^2^_A_**) and dominance (**δ^2^_D_**) genetic variance components, Baker’s ratio, and broad-sense heritability for agronomic and physiological traits of finger millet under stress (ST) and non-stress (NST) conditions.

Genetic parameters	Growing conditions	Traits										
		PTH	DTF	EL	FL	NT	NF	DTM	GY	HI	TSW	SPADM
δ^2A^	ST	37.32	40.60	3.02	2.35	0.20	0.49	37.34	0.18	6.82	0.06	7.81
	NST	120.24	22.26	6.07	4.80	0.67	0.94	6.50	0.27	4.60	0.03	6.17
δ^2D^	ST	29.55	0.77	1.23	1.20	0.38	0.33	5.85	0.30	4.10	0.05	4.83
	NST	12.33	8.58	1.30	1.08	1.35	0.20	20.62	0.08	1.25	0	0.48
Baker’s ratio	ST	0.50	0.98	0.72	0.67	0.32	0.61	0.82	0.41	0.65	0.44	0.65
	NST	0.85	0.70	0.82	0.82	0.25	0.85	0.42	0.86	0.81	0.99	0.93
Broad sense heritability	ST	0.45	0.47	0.46	0.45	0.55	0.30	0.45	0.51	0.53	0.74	0.43
	NST	0.53	0.36	0.57	0.62	0.65	0.29	0.15	0.13	0.33	0.17	0.19

PTH, plant height, cm; DTF, days to flowering; EL, ear length, cm; FL, length of the longest finger, cm; NT, number of productive basal tillers per main plant; NF, the number of fingers on the main ear; DTM, days to maturity; GY, grain yield, t/ha; HI, harvest index (%); TSW, thousand seed weight (g); SPADM, chlorophyll content, chlorophyll content at maturity. δ^2A^= additive variance, and δ^2D^ = dominance variance.

The degree of dominance, estimated through Baker’s ratio, further highlighted marked differences among traits and growing conditions (**Table 8**). Under ST conditions, Baker’s ratio varied from 0.32 for NT to 0.98 for DTF. High values were also recorded for DTM (0.82), EL (0.72), and FL (0.67), whereas comparatively lower estimates were observed for GY (0.41) and TSW (0.44). Under NST conditions, the ratio ranged from 0.25 for NT to 0.99 for TSW. Particularly high estimates were also observed for SPADM (0.93), GY (0.86), NF (0.85), EL and FL (0.82 each), and HI (0.81), whereas relatively lower values were also recorded for DTM (0.42). The NST conditions generally exhibited higher Baker’s ratio estimates for most evaluated traits compared with the ST conditions (**Table 8**).

Broad-sense heritability (H²) estimates also differed considerably across traits and water regimes (**Table 8**). Under ST conditions, H² values ranged from 0.30 for NF to 0.74 for TSW. The highest estimate was recorded for TSW, followed by NT (0.55), HI (0.53), and GY (0.51), whereas moderate estimates characterized most remaining traits. Under NST conditions, H² estimates varied from 0.13 for GY to 0.65 for NT. In these growing conditions, NT, FL, EL, and PTH displayed relatively higher H² values, whereas DTM, TSW, and SPADM exhibited comparatively lower estimates. In addition, several traits showed contrasting H² patterns between ST and NST conditions (**Table 8**).

**Figure 5:**
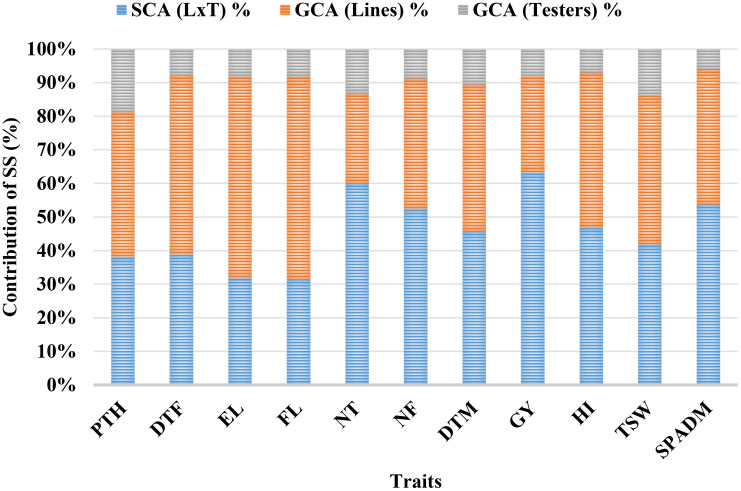
Relative contribution (percentage) of the sum squares (SS) for general combining ability (GCA) (lines or testers) and specific combining ability (SCA) (lines × testers) to the total sum of squares for plant height (PTH, cm), days to flowering (DTF), ear length (EL, cm), length of the primary finger (FL, cm), number of productive tillers per plant (NT), the number of fingers on the primary ear (NF), days to maturity (DTM), grain yield (GY, t/ha), harvest index (HI, %), thousand seed weight (TSW, g), and SPADM, chlorophyll content at maturity.

The relative contribution of combining ability components to total variation revealed distinct patterns among traits (**Figure 5**). GCA due to lines contributed substantially to total sum of squares, ranging from 26.8% for NT to 60.4% for FL. High line contributions were particularly evident for FL, EL, DTF, and PTH. In comparison, the contribution of testers remained relatively smaller, varying from 5.9% for SPADM to 18.6% for PTH. Meanwhile, SCA arising from line × tester interactions accounted for a major proportion of the total variation in several traits. The highest SCA contributions were recorded for GY (63.5%) followed by NT (60.1%), SPADM (53.7%), and NF (52.4%). Furthermore, SCA effects exceeded GCA effects for NT, NF, GY, HI, TSW, and SPADM, whereas GCA components predominated for PTH, DTF, EL, and FL ((**Figure 5**).

### 3.5 Heterosis in finger millet hybrids for agronomic and physiological traits

**Figure 6:**
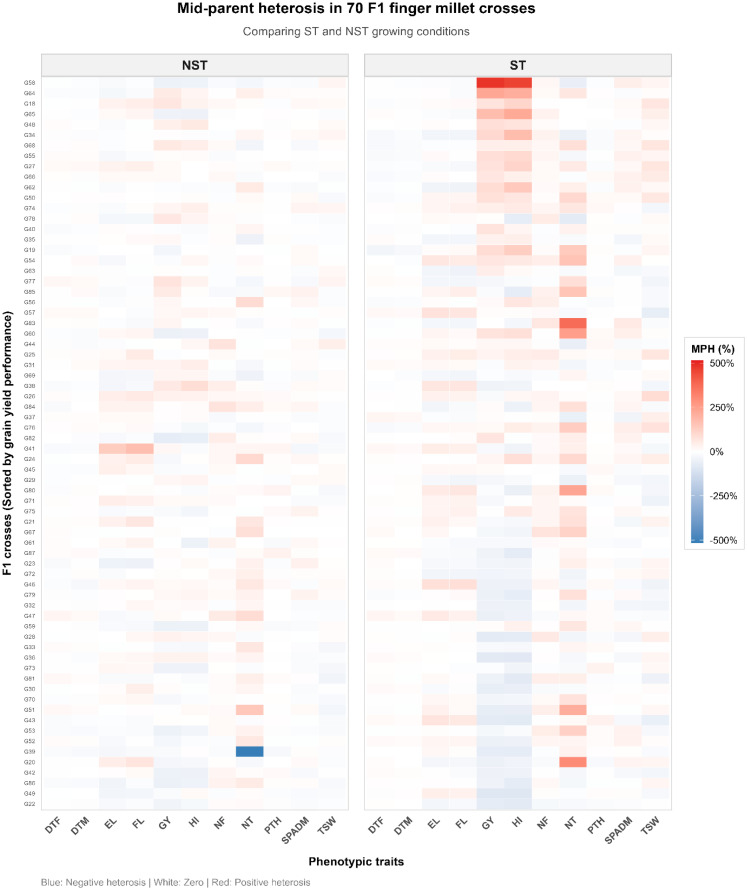
Heatmap visualization of mid-parent heterosis (MPH) for grain yield, and agronomic traits in 70 F1 finger millet crosses under drought-stressed (ST) and well-watered (NST) growing conditions. Note: color scale represents the magnitude and direction of heterosis (%), where red, blue, and white indicates positive, negative, and near zero values, respectively. Crosses are ordered based on grain yield performance.

The magnitude and direction of mid-parent heterosis (MPH) under ST and NST environments are illustrated in **Figure 6** and detailed in **Supplementary Table 6**. Under ST conditions, desirable MPH for PTH was particularly obvious in G36, G83, G82, G65, G51, G75, and G37 with values varied from −1.5% in G36 to 2.1% G37, whereas several other crosses expressed undesirable heterosis. Strong positive MPH for EL and FL was recorded in G46 (78.9% and 83.3%), G57 (75.6% and 64.9%), G43 (68.2% and 58.3%), G54 (63.1% and 50.9%), G38 (62.5% and 64.3%), and G80 (54.9% and 63.3%), respectively, whereas negative heterotic effects for these traits were also observed in crosses such as G63, G69, G34, and G29. Similarly, NT exhibited exceptionally high MPH in G83 (375.0%), G20 (300.0%), G60 (250.0%), G80 (233.3%), and G51 (211.1%), whereas NF MPH was highest in G67 (69.7%) together with G83 (60.0%), G28 (56.3%), and G78 (50.0%). In contrast, desirable reduction in both DTF and DTM was most notable in G19 (−35.8% and −26.1%), with G76 (−23.0% and −16.6%), G68 (−22.0% and −14.1%), G34 (−21.3% and −12.5%), and G55 (−19.5% and −13.5%) ranking next in the same order, although delayed flowering and maturity were also evident in several crosses including G37, G47, G57, and G41. Positive MPH for GY was extraordinarily high in G58 (483.8%), G64 (228.0%), G65 (172.5%), G34 (101.9%), G19 (88.3%), G62 (87.7%), G55 (82.7%), and G48 (82.3%). The same crosses also displayed remarkable HI MPH ranging from 72.4% in G48 to 460.6% in G58, whereas many crosses exhibited negative heterosis for both GY and HI particularly G49, G36, G43, G28, G22, and G39. Similarly, TSW heterosis varied from 18.8% in G62 to 43.0% in G65, whereas SPADM improvement ranged from 7.0% in G49 to 54.8% in G83, despite reductions in SPADM being observed in several crosses under ST conditions (**Figure 6** and **Supplementary Table 6**).

Under NST conditions, required MPH for PTH was recorded in G54, G56, G30, G73, G33, and G79 with MPH ranging from −0.4% in G79 to 0.3% in G54 and G56, whereas multiple other crosses exhibited unfavorable BPH. Positive heterosis for EL and FL was particularly notable in G41 (134.0% and 167.1%), G20 (46.5% and 68.9%), G26 (44.6% and 54.7%), G71 (44.4% and 35.6%), G21 (37.1% and 63.0%), G24 (36.8% and 49.7%), and G18 (32.7% and 45.2%), respectively, although strong negative heterosis for these traits was also expressed in crosses such as G23, G86, G49, G69, and G38. Likewise, NT recorded substantial MPH in G51 (143.5%), G24 (100.0%), G56 (92.0%), and G47 (91.1%), whereas NF heterosis was highest in G44 (73.8%), G84 (72.0%), G47 (52.9%), G82 (46.0%), and G38 (44.9%). For earliness traits, G46 expressed the most desirable combined reduction in DTF and DTM (−11.9% and −24.4%), followed by G39 (−16.3% and −23.3%), G86 (−13.8% and −17.3%), G53 (−14.1% and −17.1%), and G41 (−18.1% and −16.3%), in the same order; however, delayed DTF and DTM were observed in most crosses such as G47, G77, G51, and G81. Remarkably positive MPH for GY recorded in G77 (69.3%), G18 (64.7%), G78 (58.9%), G68 (58.7%), G38 (57.0%), G64 (52.2%), and G85 (51.4%). Correspondingly, HI heterosis varied from 22.2% in G69 and G79 (22.2%) to 86.8% in G38. Similarly, TSW MPH ranged from 13.5% in G29 to 40.9% in G44, whereas SPADM MPH was highest in G23 (36.5%) succeeded by G85 (33.1%), G79 (32.5%), and G75 (30.9%) (**Figure 6** and **Supplementary Table 6**).

Better-parent heterosis (BPH) under ST conditions followed trends comparable with MPH, with several crosses revealing strong superiority over their better parent. For example, for PTH, desirable BPH was measured in G82, G85, G87, G33, G49, G57, G78, G29, G53, G24, G38, G21, and G37 in the range of −1.5% to 1.3%, whereas a number of other crosses showed unwanted BPH. Substantial positive BPH for EL was observed in G43 (63.2%), G46 (61.8%), G57 (56.5%), G47 (53.6), G38 (52.9%), and G54 (39.5%), whereas FL BPH reached 55.7%, 67.8%, 46.9%, 31.3%, 55.9%, and 34.4% in the same crosses, respectively. Nevertheless, many crosses also exhibited negative heterosis for EL and FL. Likewise, NT exhibited exceptionally high BPH values in G83 (375.0%), G20 (166.7%), G80 (150%), G54 (133.3%), and G51(133.3%), whereas reduction was evident in several other crosses. Positive BPH for NF was also noted in G83 (45.5%), G54 and G51 (35.5% each), and G85 (31.0%), although negative BPH was frequently recorded. Conversely, beneficial negative BPH for DTF and DTM was evident in G19 with −38.6% and −28.6%, G76 at −23.8 and −17.1%, and in G68 with −25.1 and −14.8%, respectively. Additional reductions in DTF and DTM were observed in G34, G27, G40, G55, and G66. BPH for GY was highest in G65 (99.2%), followed by G50 (67.0%), G27 (54.8%), G55 (34.1%), G18 (20.3%), and G62 (12.5%). The same crosses also exhibited substantial positive BPH for HI, with values ranging from 34.5% in G55 to 143.1% in G65. A comparable trend was observed for TSW in which BPH ranged from 7.1% in G55 to 54.1% in G18, whereas SPADM heterosis varied from 3.3% in G27 to 24.1% in G62.

**Figure 7:**
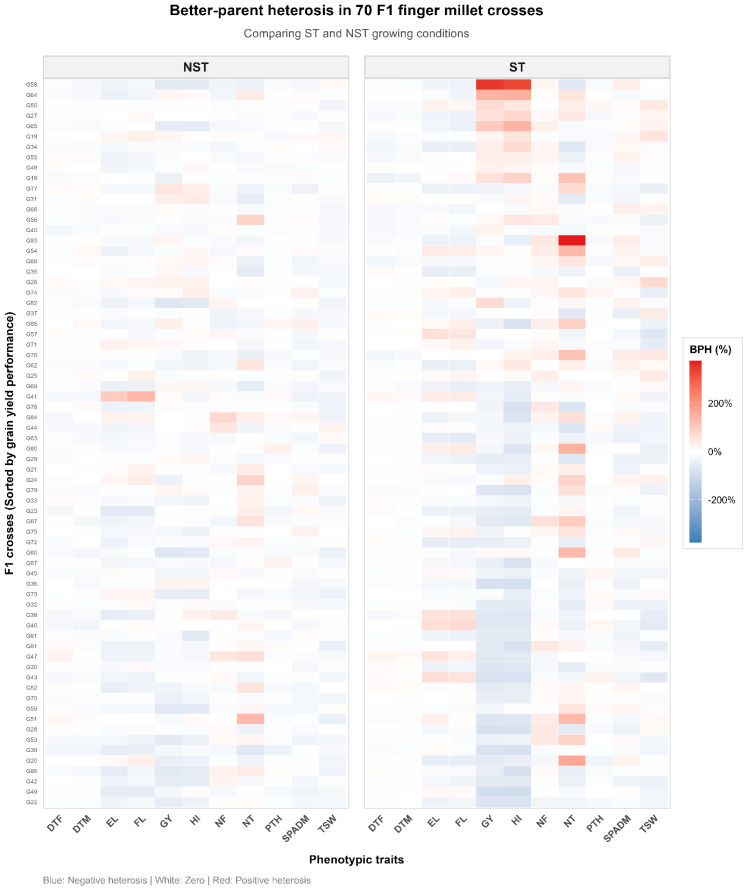
Heatmap visualization of mid-parent heterosis (MPH) for grain yield and agronomic traits in 70 F1 finger millet crosses under drought-stressed (ST) and well-watered (NST) growing conditions. Note: color scale represents the magnitude and direction of heterosis (%), where red, blue, and white indicates positive, negative, and near zero values, respectively. Crosses are ordered based on grain yield performance.

Under NST conditions, desirable BPH for PTH was observed in G82 (−0.9%), G21 (−0.4%), G75 (−0.2%), G24 (0.9%), G46, and G57 (G1.1%, each). Positive BPH for EL was highest in G41 (100%), followed by G71 (26.8%), G84 (25.5%), G73 (21.9%), G24 (19.3%), and G18 (16.8%), whereas the corresponding FL BPH varied from G71 (14.3%) to G41 (135.4%). Likewise, NT exhibited strong positive BPH in G51 (133.3%), G56 (84.6%), G24 (81.2%), G47 (59.3%), G67 (55.6%), and G52 and G62 (50.0%, each). Positive heterosis for NF was particularly high in G84 (72.0%), followed by G44 (47.2%), G47 (44.4%), G38 (38.9%), and G86 (24.1%). For earliness traits, beneficial negative BPH for both DTF and DTM was most pronounced in G39 with −25.5% and −24.0%, respectively, followed by G41 (−19.4% and −19.8%), G46 (−18.8% and −26.4%), G40 (−22.8% and −11.6%), G38 (−21.5% and −13.0%), and G86 (−14.3% and −23.0%). In contrast, several crosses displayed positive heterosis for DTF and DTM. Positive BPH for GY was highest in G77 (48.2%), succeeded by G31 (28.6%), G26 (26.9%), G36 (23.5%), and G64 (18.6%). The same crosses also showed improved BPH for HI, ranging from 9.2% in G64 to 39.3% in G31. Similarly, positive BPH for TSW ranged from 2.2% in G77 to 14.4% in G18, with additional improvement noted in G44, G58, G34, G63, and G46, whereas substantial reduction in TSW were detected in several other crosses. SPADM BPH was highest in G85 (31.2%), closely followed by G79 (29.1%), G75 (27.2%), G74 (27.0%), G18 (11.7%), and G64 (7.0%), although negative SPADM BPH predominated across much of the population under NST conditions (**Figure 7** and **Supplementary Table 7**).

## 4 Discussion

The present study conducted a detailed genetic analysis of yield and yield-influencing traits in finger millet using a line × tester mating design with 7 lines and 10 testers. The findings provide critical insights for hybrid and population improvement breeding programs in this under-researched crop.

### 4.1 Analysis of variance and mean trait performance of finger millet genotypes under contrasting moisture regimes

The combined analysis of variance (ANOVA) revealed significant (P<0.001) variability among the parents, F_1_ hybrids, and their interactions for all assessed traits under stressed (ST) and non-stressed (NST) conditions (**Table 4**). This substantial variability underscores the presence of a rich reservoir of heritable genetic diversity within the studied Ethiopian finger millet germplasms, a prerequisite for successful selection and hybrid development.

The mean performance data revealed that drought stress caused a severe reduction in grain yield, averaging approximately 57% in lines and 62% in crosses (**Figures 2**-**4**, and **Supplementary Table 2**). This substantial yield reduction is consistent with earlier findings in finger millet and other millets (Presterl and Weltzien, 2003; Maqsood and Ali, 2007; Gebreyohannes et al., 2025a), although several crosses, including G40 (4.4 t/ha), G38 (3.7 t/ha), and G37 (3.5 t/ha), maintained relatively high yields under ST conditions. This differential response suggests that heterosis can act as a buffer against environmental stress, a phenomenon well documented in pearl millet (Presterl and Weltzien, 2003). The identification of early-maturing genotypes like G41 (90 days) is particularly significant because earliness is a key adaptive trait for escaping terminal drought. This finding is consistent with reports in finger millet (Assefa et al., 2013; Mude et al., 2020; Mukami et al., 2020), Pearl millet (Yadav et al., 2011) and wheat (Semahegn et al., 2021; Thungo et al., 2022). In the same way, crosses such as G47, G38, and G79, with higher chlorophyll content values were recorded under ST, suggesting that agro-physiological traits are linked with sustained photosynthetic capacity. Higher chlorophyll content values are reported as a critical adaptive trait associated with drought tolerance in cereals (Jordan et al., 2012; Borrell et al., 2014; Shin et al., 2020; Gebreyohannes et al., 2025a, 2025b).

### 4.2 Combining ability variance for lines, testers, and lines × testers interactions across water regimes

The ANOVA for combining ability displayed that the mean squares due to lines (GCA_L_), testers (GCA_T_), and their interaction (SCA) showed high significance (P<0.001) for majority of traits under ST and NST conditions (**Table 5**). The significant GCA_L_ and GCA_T_ mean squares indicate that additive gene effect contributed substantially to the genetic variation, whereas the significant SCA mean squares indicates that non-additive gene effects (dominance and epistasis) also played an important role. These findings are consistent with previous reports in various crops including pearl millet (Presterl and Weltzien, 2003), finger millet (Owere et al., 2016), and wheat (Thungo et al., 2022). The significant GCA × W and SCA × W interactions observed in the present study further demonstrate that the combining ability effects were environment-specific, suggesting that selection of best parents and crosses should be undertaken across multiple environments.

Under NST conditions, the larger magnitude of GCA mean square compared with SCA mean squares for traits such as plant height, days to 50% flowering, ear length, and harvest index suggests a predominance of additive gene action in favorable environments. This pattern is consistent with the findings in foxtail millet and tef (Siles et al., 2004; Abraha et al., 2018). Conversely, under ST conditions, the SCA mean squares for grain yield and number of tillers were comparable with or larger than GCA mean squares, indicating a heightened role of non-additive effects under drought stress. This shift aligns with the observations in pearl millet, sorghum, and maize, where stress environments amplify non-additive genetic effects (Presterl and Weltzien, 2003; Worku et al., 2008; El-Sherbeny et al., 2019). Consequently, breeding strategies that increase yield under optimal conditions may not adequately address the needs of resource-poor farmers under stress, thereby necessitating targeted stress breeding program.

### 4.3 Combining ability effects of finger millet genotypes

The general combining ability (GCA) and specific combining ability (SCA) effects identified superior parental lines with desirable combining ability and specific cross combinations exhibiting outstanding performance for grain yield and related traits under both ST and NST conditions (**Table 6**, **7**). In the present study, parents such as G3, G2, and G4 (lines) and G14, G8, G13, G17, and G10 (tester) showed a significant and desirable GCA effect across environments (**Table 6**). Notably, line G3 was the most promising parent with higher and positive GCA effects for grain yield, harvest index, and chlorophyll content. The line is the best candidate for future crosses to combine high yield and early maturity across drought-stressed and non-stressed conditions. A higher GCA effect is linked with additive gene effects, which have minor and cumulative genetic effects that govern the average performance of progenies. Furthermore, lines G1 and G2 were identified as desirable parents for panicle-related traits such as ear and finger length, whereas G2 had better grain yield and harvest index values. Parents such as G7 are superior with higher thousand seed weight and chlorophyll content, whereas G6 also excelled for thousand seed weight, making both lines promising donors for enhancing seed weight and physiological vigor in finger millet breeding under drought-prone environments. Tester G13 has higher GCA effects for early flowering and maturity (**Table 6**), suggesting its potential in drought tolerance breeding. Selection of parents with favorable GCA effects for early maturation and yield stability, and physiological attributes is critical for breeding under water-limited conditions in various cereal crops, such as finger millet (Owere et al., 2016; Mapako and Maphosa, 2025), pearl millet (Rouamba et al., 2024), tef (Abraha et al., 2018), wheat (Thungo et al., 2022; Shamuyarira et al., 2023), barley (Yadav et al., 2022), and sorghum (Mengistu et al., 2020).

The specific combining ability (SCA) analysis revealed unique hybrid combinations with significant non-additive genetic effects, highlighting the potential for exploiting heterosis in finger millet. The SCA in the present study identified unique crosses such as G38, G70, G63, G64, G79, and G32 (**Table 7**) under stressed and non-stressed conditions. Higher SCA genetic effects imply non-additive gene action attributable to the deviation of a hybrid’s performance from its expected value based on GCA of parents (Singh and Chaudhary, 1985; Acquaah, 2012). The aforementioned crosses showed highly significant positive SCA effects for grain yield and harvest index across water conditions. These hybrids are recommended for production after stability tests and recommended for further selection and genetic advancement through pure-line or recurrent selection to develop high-yielding stable cultivars. Additionally, crosses G41, G53, G84, and G19 had low SCA values in an acceptable trend for early flowering and maturity, suggesting their potential for moisture-limited agroecologies. Crosses G21, G75, G47, G79, and G23 had higher SCA effects for chlorophyll content, which could enhance efficient photosynthesis under stress conditions. Previous reports in finger millet (Owere et al., 2016; Mapako and Maphosa, 2025) and other cereals such as pearl millet (Kumawat et al., 2019), wheat (Mwadzingeni et al., 2018; Semahegn et al., 2021), and barley (Zhang et al., 2015) consistently demonstrate the predominance of non-additive gene action governing yield stability and drought-adaptive traits under stress-prone environments.

### 4.4 Gene action, degree of dominance, heritability, and contribution to total variance

The estimates of additive variance (**δ^2A^**), dominance variance (**δ^2D^**), Baker’s ratio, broad-sense heritability (H²), and contribution to total variance are presented in **Table 8** and **Figure 5**. Under ST conditions, the genetic architecture of yield and related traits in finger millet was predominantly shaped by non-additive gene action. Baker’s ratios were considerably lower for grain yield (0.41), number of tillers (0.32), and thousand seed weight (0.44), indicating that non-additive effects played a dominant role. This shift toward non-additive control is in agreement with reports across several millet species, namely, pearl millet, sorghum, and Maize (Presterl and Weltzien, 2003; Worku et al., 2008; El-Sherbeny et al., 2019). Consequently, direct evaluation of hybrid combinations is essential for capturing heterosis gains. Importantly, H² for grain yield was higher under ST (0.51) than under NST conditions (0.13), a phenomenon often referred to as stress-induced heritability, suggesting that a larger proportion of the phenotypic variance is genetic under ST. Finally, the proportional contribution to total variance (**Figure 5**) confirmed that SCA dominated the genetic variance for grain yield (63.5%), number of productive tillers (60.1%), and number of fingers per head (52.4%), indicating that heterosis breeding, coupled with strategic selection under stress, is the most promising approach for improving drought tolerance.

Under NST conditions, the genetic control shifted markedly toward additive gene action. The Baker’s ratios observed for plant height, days to flowering, ear length, finger length, grain yield, harvest index, thousand seed-weight, and chlorophyll content, all approaching unity (**Table 8**), confirming that additive genetic effects were the primary drivers of trait expression in favorable environments. This pattern aligns with numerous reports across millets and other cereals (Siles et al., 2004; Abraha et al., 2018; Semahegn et al., 2021). Because additive genetic variance is fixable and transmissible across generations, early-generation selection methods such as pedigree breeding, single seed descent, and recurrent selection can be effectively employed to accumulate favorable alleles (Falconer and MacKay, 1996; Ubi et al., 2001). The relatively lower H^2^ for grain yield under NST (0.13) compared with ST (0.51) indicates that environmental variance contributed more substantially to phenotypic variation, necessitating robust multienvironment testing to accurately identify superior genotypes. Furthermore, the contribution of GCA to total variance was substantially higher than that of SCA for most traits under NST (**Figure 5**), reinforcing that pure-line development and population improvement are the most efficient routes for genetic advancement in finger millet when grown under NST conditions.

### 4.5 Heterosis in finger millet hybrids for agronomic and physiological traits

Heterosis is a measure of the superiority of hybrid performance relative to its parental lines. Heterosis arises from gene interaction due to dominance, overdominance, and epistatic gene actions (Li et al., 2008; Acquaah, 2012; Liang et al., 2015; Viana, 2023). In the present study, crosses G18, G54, and G79 had higher BPH for grain yield, earliness, and chlorophyll content (**Figure 7**, and **Supplementary Table 7**). These values are consistent with the SCA effects (**Table 7**). Consequently, specific crosses displaying high heterosis for physiological traits were better equipped to buffer drought impacts, leading to improved agronomic performance. Crosses G19 and G18 had high heterosis for early flowering and maturity, and better grain yield, harvest index, and chlorophyll content. Therefore, there is a possibility of developing short-duration, drought-tolerant, and high-yielding finger millet genotypes. The crosses G49 and G79 showed high heterosis for days to flowering, maturity, and chlorophyll content (**Figures 6** and **7**, **Supplementary Tables 6** and **7**), indicating the contribution of hybrid vigor to improve both productivity and stress adaptation. Earlier reports found significant and better heterosis for grain yield and drought-related traits in finger millet (Parashuram et al., 2011; Krishna et al., 2020). Higher heterosis values for chlorophyll content in some crosses, such as G18, G24, G54, G75, G79, and G84, support the role of delayed senescence and sustained photosynthetic activity in buffering drought impacts, an important adaptation mechanism in millets, including sorghum and pearl millet (Abebe et al., 2021; Sehgal et al., 2015). The heterotic gains for early maturity and enhanced harvest index highlight a crucial breeding pathway for climate-smart finger millet hybrids. This aligns with other small millets such as foxtail millet and proso millet, where hybridization improved earliness and resource-use efficiency (Siles et al., 2004; Shi et al., 2021). Some unique crosses (i.e., G18, G19, G25, and G37) were selected with high heterotic performance for earliness, high chlorophyll content, and better grain yield. These crosses are promising genetic resources for selection and the advancement of the derived progenies to fix desirable alleles as elite pure lines.

## 5 Conclusions

The current study evaluated the potential of hybrid breeding and new population development in finger millet to enhance yield gains and improve drought resilience. The line × tester mating design revealed variation in genetic effects, including general combining ability due to lines, and testers and specific combining ability of line × tester in finger millet for agronomic and physiological traits. Additive genetic effect predominantly conditioned the inheritance of days to flowering, ear length, and days to maturity under drought stressed conditions, whereas non-additive effects affected the number of productive basal tillers per main plant and days to maturity under non-stressed conditions. Broad-sense heritability for grain yield was higher under stressed (H^2^ = 0.51) than non-stressed (0.13) conditions, necessitating multiple testing environments for drought tolerance evaluation and genotype selection. The study identified lines such as G3, G2, and G4 and testers such as G14, G8, G13, G17, and G10 which exhibited higher general combining ability effects for grain yield in a desirable trend. Crosses G85, G31, G79, G38, G48, G54, G64, and G65 were identified and exhibited higher specific combining ability effects and heterosis for grain yield under drought-stressed conditions. The selected parents and the top crosses are recommended for breeding and selection of new generation and drought-adapted finger millet genotypes.

## Data Availability

The original contributions presented in the study are included in the article/**Supplementary Material**. Further inquiries can be directed to the corresponding author.
