# Supplementary material for: Hybrid performance in finger millet (Eleusine coracana) for grain yield and component traits under well-watered and drought-stress conditions
Source: Front Plant Sci. 2026 Jul 10;17:1770566. doi: 10.3389/fpls.2026.1770566 (PMC13396158; doi:10.3389/fpls.2026.1770566)
Supplement: Supplementary file 1 [file Table1.docx]

Supplementary Table S1. Lists of F1 families of finger millet used in the study

| **Cross/family** | **Code for family** |  | **Cross/family** | **Code for family** |
| --- | --- | --- | --- | --- |
| 227974/213835 | G18 |  | Addis-01/Bako-09 | G53 |
| 227974/215982 | G19 |  | Addis-01/Gute | G54 |
| 227974/238335 | G20 |  | Addis-01/Kako-1 | G55 |
| 227974/24394 | G21 |  | Addis-01/Meba | G56 |
| 227974/AxumColl21 | G22 |  | Addis-01/Mereb-1 | G57 |
| 227974/Bako-09 | G23 |  | Necho/213835 | G58 |
| 227974/Gute | G24 |  | Necho/215982 | G59 |
| 227974/Kako-1 | G25 |  | Necho/238335 | G60 |
| 227974/Meba | G26 |  | Necho/24394 | G61 |
| 227974/Mereb-1 | G27 |  | Necho/AxumColl21 | G62 |
| 234187/213835 | G28 |  | Necho/Bako-09 | G63 |
| 234187/215982 | G29 |  | Necho/Gute | G64 |
| 234187/238335 | G30 |  | Necho/Kako-1 | G65 |
| 234187/24394 | G31 |  | Necho/Meba | G66 |
| 234187/AxumColl21 | G32 |  | Necho/Mereb-1 | G67 |
| 234187/Bako-09 | G33 |  | Tessema/213835 | G68 |
| 234187/Gute | G34 |  | Tessema/215982 | G69 |
| 234187/Kako-1 | G35 |  | Tessema/238335 | G70 |
| 234187/Meba | G36 |  | Tessema/24394 | G71 |
| 234187/Mereb-1 | G37 |  | Tessema/AxumColl21 | G72 |
| 235700/213835 | G38 |  | Tessema/Bako-09 | G73 |
| 235700/215982 | G39 |  | Tessema/Gute | G74 |
| 235700/238335 | G40 |  | Tessema/Kako-1 | G75 |
| 235700/24394 | G41 |  | Tessema/Meba | G76 |
| 235700/AxumColl21 | G42 |  | Tessema/Mereb-1 | G77 |
| 235700/Bako-09 | G43 |  | Wama/213835 | G78 |
| 235700/Gute | G44 |  | Wama/215982 | G79 |
| 235700/Kako-1 | G45 |  | Wama/238335 | G80 |
| 235700/Meba | G46 |  | Wama/24394 | G81 |
| 235700/Mereb-1 | G47 |  | Wama/AxumColl21 | G82 |
| Addis-01/213835 | G48 |  | Wama Bako-09 | G83 |
| Addis-01/215982 | G49 |  | Wama/Gute | G84 |
| Addis-01/238335 | G50 |  | Wama/Kako-1 | G85 |
| Addis-01/24394 | G51 |  | Wama/Meba | G86 |
| Addis-01/AxumColl21 | G52 |  | Wama/Mereb-1 | G87 |

Supplementary Table S2. Mean performance of 7 lines and 10 testers for agronomic and physiological traits under stress and non-stress growing conditions across glasshouse and field environments.

| Drought  condition | Traits | Lines | | | | | | | | Testers | | | | | | | | | | |
| --- | --- | --- | --- | --- | --- | --- | --- | --- | --- | --- | --- | --- | --- | --- | --- | --- | --- | --- | --- | --- |
|  |  | G1 | G2 | G3 | G4 | G5 | G6 | G7 | Mean | G8 | G9 | G10 | G11 | G12 | G13 | G14 | G15 | G16 | G17 | Mean |
| ST | PTH | 127.0 | 114.5 | 98.3 | 105.0 | 121.3 | 122.0 | 118.5 | 115.2 | 118.5 | 109.0 | 124.3 | 103.3 | 114.8 | 83.8 | 113.5 | 101.8 | 97.8 | 112.8 | 112.8 |
| NST |  | 138.0 | 118.8 | 94.0 | 105.3 | 137.8 | 141.0 | 118.8 | 122.0 | 126.5 | 127.8 | 134.3 | 116.0 | 134.3 | 98.8 | 120.0 | 109.0 | 116.5 | 108.3 | 108.3 |
| ST | DTF | 88.8 | 86.5 | 75.8 | 91.0 | 94.5 | 92.5 | 98.5 | 89.7 | 81.0 | 100.5 | 97.3 | 94.5 | 89.0 | 86.0 | 96.5 | 87.3 | 94.5 | 67.8 | 67.8 |
| NST |  | 94.0 | 95.0 | 78.8 | 90.5 | 104.8 | 92.8 | 94.3 | 92.9 | 81.3 | 94.3 | 101.0 | 96.5 | 84.5 | 88.3 | 101.3 | 87.8 | 93.3 | 74.5 | 74.5 |
| ST | EL | 12.8 | 11.9 | 8.5 | 6.8 | 13.0 | 10.5 | 9.9 | 10.5 | 7.5 | 7.5 | 11.3 | 6.8 | 14.5 | 8.0 | 9.5 | 8.0 | 6.9 | 8.6 | 8.6 |
| NST |  | 14.1 | 14.0 | 5.5 | 7.5 | 16.3 | 10.3 | 11.8 | 11.4 | 7.8 | 10.8 | 14.1 | 7.4 | 18.0 | 10.3 | 10.5 | 9.3 | 8.0 | 7.4 | 7.4 |
| ST | FL | 10.8 | 10.0 | 7.4 | 6.3 | 12.3 | 8.3 | 8.0 | 9.0 | 7.8 | 6.6 | 10.3 | 5.5 | 12.3 | 7.6 | 8.0 | 6.5 | 6.1 | 8.0 | 8 |
| NST |  | 10.9 | 11.3 | 4.6 | 5.5 | 12.3 | 8.8 | 9.5 | 9.0 | 6.0 | 8.8 | 11.8 | 6.0 | 12.5 | 9.3 | 8.5 | 7.5 | 6.3 | 6.8 | 6.8 |
| ST | NT | 1.5 | 3.0 | 2.5 | 1.5 | 1.5 | 1.5 | 1.0 | 1.8 | 0.8 | 1.0 | 1.3 | 0.5 | 1.8 | 1.0 | 1.3 | 2.0 | 1.8 | 1.8 | 1.8 |
| NST |  | 4.0 | 6.8 | 6.8 | 3.0 | 3.3 | 4.8 | 4.0 | 4.7 | 2.8 | 4.0 | 3.5 | 3.5 | 3.5 | 3.8 | 3.3 | 4.8 | 3.3 | 4.5 | 4.5 |
| ST | NF | 9.3 | 8.8 | 10.3 | 7.8 | 7.8 | 8.0 | 6.8 | 8.4 | 7.0 | 7.3 | 9.5 | 9.0 | 10.5 | 8.3 | 6.8 | 7.3 | 8.0 | 8.8 | 8.8 |
| NST |  | 11.0 | 10.0 | 9.0 | 8.8 | 12.3 | 9.3 | 6.3 | 9.5 | 7.3 | 8.3 | 10.5 | 8.8 | 9.5 | 9.5 | 6.3 | 9.0 | 7.3 | 8.0 | 8 |
| ST | DTM | 117.3 | 114.8 | 105.8 | 119.5 | 120.8 | 121.5 | 122.5 | 117.5 | 111.0 | 123.5 | 125.8 | 123.0 | 117.5 | 114.5 | 118.5 | 115.8 | 123.0 | 97.8 | 97.8 |
| NST |  | 132.3 | 136.3 | 136.0 | 129.5 | 159.5 | 122.8 | 124.3 | 134.4 | 124.5 | 124.3 | 138.5 | 126.5 | 141.5 | 128.0 | 131.3 | 129.5 | 143.8 | 104.5 | 104.5 |
| ST | GY | 1.8 | 2.6 | 3.5 | 1.9 | 0.4 | 2.6 | 2.1 | 2.1 | 2.9 | 0.8 | 1.2 | 1.7 | 2.1 | 2.6 | 0.8 | 0.9 | 1.9 | 2.4 | 2.4 |
| NST |  | 4.3 | 4.4 | 5.9 | 6.2 | 3.4 | 4.3 | 5.5 | 4.9 | 4.4 | 1.8 | 5.3 | 7.0 | 6.1 | 4.9 | 6.1 | 4.0 | 3.8 | 3.2 | 3.2 |
| ST | HI | 6.8 | 11.1 | 17.2 | 8.9 | 1.6 | 10.3 | 23.4 | 11.3 | 12.8 | 3.1 | 4.2 | 6.8 | 9.4 | 13.8 | 2.7 | 3.3 | 7.0 | 10.1 | 10.1 |
| NST |  | 11.5 | 14.8 | 18.6 | 14.8 | 9.8 | 11.4 | 15.9 | 13.8 | 13.8 | 6.6 | 12.1 | 17.8 | 15.8 | 16.9 | 13.6 | 12.5 | 12.7 | 11.2 | 11.2 |
| ST | TSW | 0.8 | 1.5 | 1.9 | 1.5 | 1.5 | 1.4 | 1.7 | 1.5 | 1.7 | 0.9 | 1.4 | 1.2 | 1.7 | 1.5 | 1.1 | 1.1 | 0.9 | 1.7 | 1.7 |
| NST |  | 1.6 | 2.1 | 2.2 | 2.1 | 2.1 | 3.0 | 3.2 | 2.3 | 2.7 | 1.7 | 1.8 | 2.6 | 2.4 | 2.5 | 1.6 | 2.7 | 2.5 | 1.8 | 1.8 |
| ST | SPADM | 24.2 | 27.3 | 29.3 | 18.9 | 21.0 | 20.3 | 22.2 | 23.3 | 29.7 | 23.5 | 19.2 | 22.1 | 24.8 | 30.4 | 22.7 | 22.6 | 22.7 | 32.6 | 32.6 |
| NST |  | 27.8 | 40.6 | 39.4 | 23.8 | 28.8 | 27.8 | 30.3 | 31.2 | 36.5 | 31.6 | 31.9 | 34.4 | 37.6 | 39.9 | 28.5 | 29.4 | 31.8 | 36.2 | 36.2 |

Note: ST: stress, and NST: non stress growing conditions, LSD (0.05): least significance difference at 5%; NS: non-significant, G1: 227974, G2: 234187, G3: 235700, G4: Addis-01, G5: Necho, G6: Tessema, G7: Wama, G8: 24394, G9: 213835, G10: 215982, G11: 238335, G12: AxumColl21 , G13: Bako-09, G14: Gute, G15: Kako-1, G16: Meba, and G17: Mereb-1; plant height (cm, PTH), days to flowering (DTF), ear length, (cm, (EL), length of the primary finger, (cm, FL), number of productive tillers per plant (NT), the number of fingers on the primary ear (NF), days to maturity (DTM), grain yield (t/ha, GY), harvest index (%, HI), thousand seed weight (g, TSW), and SPADM, chlorophyll content at maturity).

Supplementary Table S3. Agronomic and physiological trait performance for the top 15 and bottom five finger millet crosses ranked according to their grain yield (t/ha) under stress growing conditions across glasshouse and field environments.

| Drought condition | Traits | Top fifteen | | | | | | | | | | | | | | | Bottom five | | | | | Mean* | LSD* (0.05) |
| --- | --- | --- | --- | --- | --- | --- | --- | --- | --- | --- | --- | --- | --- | --- | --- | --- | --- | --- | --- | --- | --- | --- | --- |
|  |  | **G40** | **G38** | **G23** | **G48** | **G43** | **G44** | **G83** | **G27** | **G56** | **G39** | **G57** | **G72** | **G35** | **G34** | **G47** | **G68** | **G75** | **G76** | **G78** | **G59** |  |  |
| ST | **PTH** | 106.5 | 116.8 | 108.8 | 110.8 | 123.3 | 111.5 | 111.5 | 106.0 | 102.5 | 107.3 | 105.3 | 107.3 | 122.5 | 124.5 | 101.8 | 114.5 | 116.8 | 107.0 | 106.0 | 107.5 | 112.5 | 18.29 |
| NST |  | 95.8 | 123.5 | 111.8 | 105.0 | 124.3 | 129.0 | 129.0 | 121.3 | 116.5 | 124.5 | 99.8 | 120.3 | 122.0 | 132.0 | 108.5 | 121.0 | 135.8 | 112.3 | 116.0 | 114.5 | 121.3 | 19.1 |
| ST | **DTF** | 84.8 | 80.3 | 79.8 | 70.5 | 80.5 | 75.3 | 92.0 | 82.8 | 86.8 | 83.0 | 79.5 | 83.5 | 83.8 | 92.0 | 81.3 | 95.5 | 84.3 | 94.5 | 92.8 | 92.8 | 90.0 | 30.81 |
| NST |  | 77.3 | 82.3 | 80.0 | 75.5 | 79.3 | 76.0 | 93.3 | 83.3 | 92.0 | 81.3 | 82.3 | 86.5 | 82.8 | 80.3 | 80.5 | 97.0 | 71.3 | 91.3 | 88.5 | 94.0 | 90.8 | 33.5 |
| ST | **EL** | 10.3 | 10.1 | 8.8 | 13.0 | 14.9 | 7.6 | 10.8 | 11.8 | 11.0 | 8.4 | 8.1 | 7.8 | 9.3 | 11.4 | 7.6 | 8.9 | 11.5 | 9.8 | 7.9 | 12.3 | 10.1 | 3.86 |
| NST |  | 6.8 | 9.4 | 7.5 | 11.0 | 13.3 | 9.8 | 11.8 | 12.8 | 10.0 | 10.8 | 7.0 | 9.5 | 9.8 | 12.0 | 7.8 | 11.3 | 12.1 | 10.8 | 10.5 | 12.8 | 10.8 | 4.0 |
| ST | **FL** | 9.1 | 8.5 | 7.1 | 12.0 | 13.0 | 6.6 | 9.1 | 10.8 | 9.4 | 7.0 | 6.5 | 6.8 | 10.0 | 9.0 | 4.8 | 8.3 | 10.9 | 7.5 | 7.5 | 10.3 | 8.6 | 3.5 |
| NST |  | 5.3 | 8.6 | 6.3 | 8.5 | 10.5 | 8.0 | 8.5 | 10.3 | 9.0 | 8.8 | 5.5 | 9.3 | 8.8 | 10.5 | 6.5 | 10.0 | 11.1 | 10.3 | 9.5 | 10.5 | 9.1 | 3.3 |
| ST | **NT** | 2.0 | 1.5 | 1.8 | 1.8 | 2.3 | 1.0 | 1.5 | 1.8 | 2.3 | 1.3 | 1.5 | 2.8 | 2.0 | 1.5 | 1.5 | 1.8 | 1.8 | 2.0 | 2.3 | 2.3 | 1.9 | 1.23 |
| NST |  | 4.0 | 5.5 | 3.8 | 3.5 | 4.8 | 3.8 | 4.8 | 4.0 | 5.0 | 5.5 | 6.5 | 5.5 | 5.0 | 4.0 | 5.0 | 5.0 | 5.3 | 4.8 | 4.5 | 3.8 | 4.6 | 1.9 |
| ST | **NF** | 11.5 | 9.3 | 9.5 | 8.5 | 10.0 | 7.8 | 9.3 | 8.5 | 10.3 | 9.0 | 9.8 | 10.8 | 9.0 | 7.5 | 8.3 | 7.8 | 11.5 | 11.3 | 8.0 | 10.5 | 9.2 | 2.64 |
| NST |  | 7.8 | 10.3 | 8.8 | 8.8 | 11.0 | 8.3 | 10.3 | 9.8 | 11.5 | 9.3 | 11.3 | 11.3 | 9.5 | 7.0 | 10.0 | 7.8 | 9.5 | 9.3 | 12.3 | 13.8 | 9.4 | 3.0 |
| ST | **DTM** | 110.0 | 105.8 | 109.0 | 100.5 | 109.0 | 105.3 | 119.0 | 112.0 | 108.5 | 109.8 | 109.5 | 113.3 | 110.3 | 121.8 | 110.5 | 121.3 | 112.8 | 117.8 | 123.5 | 114.8 | 116.9 | 26.89 |
| NST |  | 129.0 | 134.8 | 148.0 | 123.8 | 130.5 | 118.3 | 131.8 | 123.0 | 131.8 | 118.3 | 121.5 | 133.5 | 112.8 | 110.3 | 144.3 | 128.8 | 101.3 | 131.8 | 128.5 | 143.8 | 130.4 | 30.89 |
| ST | **GY** | 4.0 | 3.4 | 3.4 | 3.2 | 3.1 | 3.1 | 3.0 | 3.0 | 2.9 | 2.8 | 2.7 | 2.7 | 2.7 | 2.7 | 2.7 | 1.1 | 1.0 | 0.8 | 0.7 | 0.7 | 1.8 | 1.79 |
| NST |  | 6.5 | 6.2 | 6.8 | 5.5 | 5.6 | 7.0 | 6.9 | 6.4 | 4.9 | 5.2 | 5.3 | 5.5 | 5.3 | 6.6 | 7.2 | 5.9 | 2.6 | 3.0 | 4.6 | 1.7 | 4.7 | 5.01 |
| ST | **HI** | 13.6 | 13.8 | 15.1 | 14.4 | 13.1 | 14.2 | 12.3 | 14.3 | 13.0 | 12.3 | 11.5 | 12.5 | 12.7 | 17.8 | 11.8 | 4.6 | 5.2 | 2.5 | 3.8 | 3.5 | 8.2 | 8.22 |
| NST |  | 19.2 | 18.8 | 17.8 | 18.0 | 23.5 | 20.7 | 16.3 | 17.7 | 11.0 | 17.2 | 15.5 | 16.3 | 13.8 | 18.1 | 19.0 | 16.2 | 7.1 | 7.8 | 12.4 | 5.6 | 13.5 | 13.15 |
| ST | **TSW** | 1.5 | 1.4 | 1.6 | 1.6 | 1.4 | 1.6 | 1.4 | 1.1 | 1.2 | 1.8 | 1.5 | 1.8 | 1.3 | 1.7 | 1.4 | 1.3 | 1.5 | 1.6 | 1.5 | 1.6 | 1.4 | 0.36 |
| NST |  | 2.2 | 2.3 | 2.1 | 1.9 | 2.4 | 2.1 | 2.2 | 2.2 | 1.8 | 2.4 | 2.3 | 2.8 | 2.1 | 2.5 | 2.1 | 2.4 | 2.0 | 2.2 | 2.3 | 1.7 | 2.2 | 0.6 |
| ST | **SPADM** | 24.9 | 20.1 | 19.8 | 27.0 | 25.0 | 22.9 | 27.0 | 23.4 | 23.6 | 25.2 | 26.6 | 24.3 | 24.0 | 23.5 | 26.4 | 25.1 | 21.0 | 24.6 | 27.6 | 21.2 | 24.7 | 8.69 |
| NST |  | 30.9 | 27.4 | 30.1 | 34.4 | 30.9 | 29.0 | 31.0 | 28.3 | 31.7 | 35.1 | 40.2 | 38.7 | 32.6 | 28.2 | 33.6 | 32.9 | 27.7 | 31.1 | 32.8 | 27.0 | 32.7 | 12.52 |

Note: *=the statistics for the whole crosses, ST: stress, and NST: non stress growing conditions; LSD(0.05): least significance difference at 5%; plant height (cm, PTH), days to flowering (DTF), ear length, (cm, (EL), length of the primary finger, (cm,FL), number of productive tillers per plant (NT), the number of fingers on the primary ear (NF), days to maturity (DTM), grain yield (t/ha, GY), harvest index (%, HI), thousand seed weight (g, TSW), and SPADM, chlorophyll content at maturity). G40: 235700/238335, G38: 235700/213835, G23: 227974/Bako-09, G48: Addis-01/213835, G43: 235700/Bako-09, G44: 235700/Gute, G83: Wama/Bako-09, G27: 227974/Mereb-1, G56: Addis-01/Meba, G39: 235700/215982, G57: Addis-01/Mereb-1, G72: Tessema/AxumColl21 , G35: 234187/Kako-1, G34: 234187/Gute, G47: 235700/Mereb-1, G68: Tessema/213835, G75: Tessema/Kako-1, G76: Tessema/Meba, G78: Wama/213835, and G59: Necho/215982

Supplementary Table S4. Mean performance of F_1_ families of finger millet under drought-stressed conditions for agronomic and physiological traits

| Cross/family | Code | PTH | DTF | EL | FL | NF | NT | DTM | GY | HI (%) | TSW | SPADM |
| --- | --- | --- | --- | --- | --- | --- | --- | --- | --- | --- | --- | --- |
| 227974/213835 | G18 | 123.0 | 91.8 | 14.3 | 12.8 | 12.3 | 2.0 | 120.3 | 1.7 | 7.3 | 1.5 | 27.0 |
| 227974/215982 | G19 | 126.0 | 85.8 | 13.5 | 11.0 | 9.8 | 1.5 | 115.0 | 1.2 | 5.2 | 1.0 | 17.4 |
| 227974/238335 | G20 | 109.3 | 81.3 | 13.0 | 11.5 | 8.8 | 1.8 | 110.5 | 1.3 | 6.0 | 0.9 | 27.4 |
| 227974/24394 | G21 | 128.0 | 92.0 | 13.8 | 11.0 | 10.3 | 2.0 | 121.0 | 1.9 | 7.4 | 1.7 | 19.7 |
| 227974/AxumColl21 | G22 | 114.0 | 94.0 | 13.8 | 12.4 | 11.3 | 1.5 | 121.3 | 1.4 | 6.4 | 0.8 | 21.6 |
| 227974/Bako-09 | G23 | 105.0 | 83.5 | 7.9 | 6.8 | 11.8 | 2.8 | 113.5 | 1.6 | 9.7 | 1.2 | 33.0 |
| 227974/Gute | G24 | 136.0 | 92.0 | 12.0 | 10.5 | 9.0 | 2.3 | 120.5 | 0.9 | 4.0 | 1.4 | 24.5 |
| 227974/Kako-1 | G25 | 109.0 | 94.0 | 13.0 | 10.0 | 14.0 | 3.5 | 110.0 | 1.0 | 3.6 | 1.4 | 26.1 |
| 227974/Meba | G26 | 140.3 | 98.3 | 12.6 | 11.0 | 9.3 | 2.3 | 127.8 | 2.4 | 10.0 | 0.8 | 22.4 |
| 227974/Mereb-1 | G27 | 126.0 | 95.5 | 12.9 | 11.0 | 10.3 | 2.5 | 127.3 | 1.7 | 6.8 | 1.0 | 21.1 |
| 234187/213835 | G28 | 108.0 | 95.3 | 13.3 | 11.0 | 11.0 | 1.8 | 115.5 | 1.5 | 8.4 | 1.6 | 28.0 |
| 234187/215982 | G29 | 112.8 | 89.8 | 10.5 | 9.0 | 8.3 | 2.5 | 118.3 | 2.0 | 9.3 | 1.6 | 27.9 |
| 234187/238335 | G30 | 116.0 | 87.3 | 12.0 | 9.4 | 9.0 | 2.5 | 115.8 | 0.8 | 3.2 | 1.4 | 25.1 |
| 234187/24394 | G31 | 111.0 | 85.0 | 11.3 | 9.8 | 8.5 | 1.3 | 113.5 | 2.2 | 10.3 | 1.4 | 27.9 |
| 234187/AxumColl21 | G32 | 116.0 | 89.0 | 13.3 | 10.5 | 8.3 | 2.5 | 117.5 | 1.3 | 7.5 | 1.5 | 27.0 |
| 234187/Bako-09 | G33 | 105.3 | 93.8 | 13.3 | 10.8 | 10.5 | 3.5 | 109.3 | 2.3 | 9.1 | 1.3 | 27.9 |
| 234187/Gute | G34 | 128.5 | 89.5 | 12.3 | 10.0 | 8.5 | 3.5 | 118.0 | 1.8 | 7.7 | 1.4 | 30.1 |
| 234187/Kako-1 | G35 | 111.3 | 93.0 | 11.5 | 10.5 | 9.5 | 1.3 | 120.8 | 2.1 | 11.2 | 0.8 | 26.2 |
| 234187/Meba | G36 | 112.3 | 93.5 | 12.0 | 10.1 | 9.5 | 3.0 | 113.5 | 1.8 | 10.5 | 1.2 | 25.4 |
| 234187/Mereb-1 | G37 | 116.0 | 90.0 | 12.3 | 11.0 | 9.8 | 2.0 | 118.5 | 3.5 | 15.5 | 1.6 | 31.5 |
| 235700/213835 | G38 | 106.3 | 69.8 | 8.6 | 7.0 | 10.5 | 2.5 | 99.8 | 3.7 | 17.9 | 2.0 | 33.7 |
| 235700/215982 | G39 | 96.0 | 72.0 | 6.8 | 5.8 | 10.3 | 1.0 | 102.0 | 3.5 | 18.9 | 1.4 | 31.7 |
| 235700/238335 | G40 | 108.0 | 77.0 | 7.5 | 5.9 | 8.8 | 2.0 | 107.0 | 4.4 | 15.1 | 1.7 | 27.3 |
| 235700/24394 | G41 | 132.3 | 59.8 | 13.0 | 12.3 | 10.3 | 3.3 | 89.8 | 2.8 | 12.7 | 1.3 | 20.1 |
| 235700/AxumColl21 | G42 | 112.0 | 80.3 | 9.8 | 8.1 | 10.5 | 3.5 | 110.3 | 0.8 | 4.0 | 2.0 | 23.1 |
| 235700/Bako-09 | G43 | 99.0 | 71.8 | 8.3 | 6.5 | 9.3 | 1.3 | 101.8 | 2.6 | 11.9 | 1.6 | 28.0 |
| 235700/Gute | G44 | 104.8 | 84.3 | 8.3 | 7.1 | 10.3 | 2.3 | 112.8 | 2.3 | 13.2 | 2.0 | 30.8 |
| 235700/Kako-1 | G45 | 99.3 | 75.3 | 8.3 | 6.9 | 9.3 | 2.3 | 105.3 | 2.4 | 9.4 | 1.9 | 26.6 |
| 235700/Meba | G46 | 114.0 | 72.0 | 10.5 | 8.5 | 10.5 | 3.8 | 102.0 | 2.6 | 12.7 | 2.1 | 31.1 |
| 235700/Mereb-1 | G47 | 100.5 | 84.8 | 6.3 | 5.3 | 12.0 | 4.8 | 113.3 | 2.4 | 12.8 | 1.4 | 40.7 |
| Addis-01/213835 | G48 | 103.3 | 95.8 | 11.5 | 10.3 | 9.8 | 1.3 | 124.0 | 2.5 | 15.6 | 1.6 | 22.8 |
| Addis-01/215982 | G49 | 111.0 | 95.8 | 6.5 | 5.5 | 7.3 | 2.0 | 117.8 | 2.4 | 10.2 | 1.5 | 16.9 |
| Addis-01/238335 | G50 | 108.8 | 90.3 | 11.3 | 8.5 | 10.0 | 1.5 | 118.8 | 1.4 | 7.1 | 1.2 | 20.2 |
| Addis-01/24394 | G51 | 91.0 | 98.8 | 6.6 | 5.8 | 7.3 | 4.0 | 119.0 | 0.8 | 3.9 | 1.2 | 29.7 |
| Addis-01/AxumColl21 | G52 | 100.5 | 95.5 | 7.8 | 6.5 | 8.3 | 1.3 | 118.5 | 2.4 | 10.3 | 1.4 | 19.3 |
| Addis-01/Bako-09 | G53 | 88.0 | 77.5 | 7.3 | 5.9 | 11.3 | 1.5 | 107.5 | 2.2 | 13.1 | 0.9 | 20.1 |
| Addis-01/Gute | G54 | 99.3 | 95.5 | 6.8 | 5.5 | 7.5 | 1.0 | 115.5 | 2.2 | 8.9 | 1.2 | 18.7 |
| Addis-01/Kako-1 | G55 | 107.5 | 93.5 | 7.5 | 5.9 | 7.8 | 1.8 | 110.3 | 1.7 | 5.6 | 1.6 | 19.1 |
| Addis-01/Meba | G56 | 105.8 | 89.3 | 6.3 | 5.4 | 7.8 | 3.0 | 117.8 | 1.8 | 8.5 | 1.0 | 25.4 |
| Addis-01/Mereb-1 | G57 | 109.3 | 97.0 | 6.3 | 6.4 | 8.3 | 2.0 | 123.8 | 1.6 | 6.3 | 1.1 | 28.1 |
| Necho/213835 | G58 | 94.5 | 96.5 | 9.3 | 9.0 | 12.5 | 1.3 | 121.0 | 0.4 | 2.1 | 1.7 | 17.3 |
| Necho/215982 | G59 | 104.5 | 102.0 | 9.5 | 8.0 | 8.8 | 1.8 | 128.3 | 0.5 | 1.6 | 1.5 | 18.4 |
| Necho/238335 | G60 | 122.5 | 92.5 | 13.9 | 11.9 | 9.0 | 2.0 | 118.5 | 0.7 | 3.0 | 0.7 | 21.9 |
| Necho/24394 | G61 | 103.5 | 91.8 | 9.0 | 9.0 | 7.5 | 1.3 | 118.3 | 0.5 | 1.8 | 1.3 | 22.2 |
| Necho/AxumColl21 | G62 | 123.0 | 88.8 | 12.8 | 11.3 | 9.3 | 1.3 | 128.8 | 0.2 | 0.5 | 1.1 | 20.3 |
| Necho/Bako-09 | G63 | 111.8 | 94.0 | 13.5 | 11.8 | 9.3 | 1.3 | 122.5 | 2.3 | 10.1 | 0.6 | 25.4 |
| Necho/Gute | G64 | 123.3 | 93.0 | 11.8 | 8.8 | 8.3 | 2.3 | 121.5 | 2.0 | 6.9 | 1.4 | 19.1 |
| Necho/Kako-1 | G65 | 117.3 | 95.0 | 10.5 | 8.0 | 9.8 | 1.8 | 118.5 | 1.4 | 5.6 | 1.3 | 22.8 |
| Necho/Meba | G66 | 107.0 | 95.8 | 9.9 | 8.3 | 10.0 | 1.3 | 111.8 | 1.2 | 4.0 | 0.9 | 23.6 |
| Necho/Mereb-1 | G67 | 116.0 | 95.3 | 11.0 | 10.5 | 9.5 | 3.8 | 121.3 | 1.3 | 4.5 | 1.1 | 22.7 |
| Tessema/213835 | G68 | 123.8 | 96.5 | 8.6 | 6.5 | 9.0 | 1.0 | 128.3 | 1.4 | 6.2 | 1.0 | 17.4 |
| Tessema/215982 | G69 | 116.0 | 96.0 | 9.5 | 7.8 | 9.3 | 1.0 | 124.5 | 2.7 | 10.8 | 2.3 | 17.9 |
| Tessema/238335 | G70 | 126.0 | 92.8 | 10.3 | 8.4 | 8.0 | 0.8 | 121.3 | 2.6 | 12.0 | 1.3 | 20.9 |
| Tessema/24394 | G71 | 116.3 | 95.8 | 7.8 | 7.0 | 8.3 | 1.8 | 117.0 | 1.4 | 6.1 | 1.5 | 21.9 |
| Tessema/AxumColl21 | G72 | 121.0 | 80.5 | 8.5 | 7.3 | 9.0 | 2.0 | 110.5 | 3.2 | 13.0 | 2.2 | 23.9 |
| Tessema/Bako-09 | G73 | 107.8 | 88.8 | 9.1 | 7.8 | 8.3 | 1.3 | 117.3 | 1.3 | 5.6 | 1.6 | 20.8 |
| Tessema/Gute | G74 | 111.3 | 97.8 | 9.4 | 7.5 | 10.0 | 1.8 | 121.8 | 1.8 | 7.9 | 1.9 | 23.8 |
| Tessema/Kako-1 | G75 | 115.5 | 96.0 | 7.9 | 6.6 | 10.0 | 1.0 | 123.0 | 1.4 | 6.3 | 2.0 | 25.9 |
| Tessema/Meba | G76 | 117.5 | 99.8 | 10.0 | 8.0 | 10.5 | 0.3 | 120.5 | 1.5 | 4.2 | 1.5 | 21.0 |
| Tessema/Mereb-1 | G77 | 102.5 | 101.3 | 8.8 | 6.9 | 8.8 | 1.0 | 124.8 | 1.1 | 4.1 | 1.6 | 21.2 |
| Wama/213835 | G78 | 102.0 | 102.3 | 7.6 | 7.3 | 7.3 | 1.5 | 127.8 | 0.8 | 3.7 | 1.5 | 24.0 |
| Wama/215982 | G79 | 112.3 | 94.8 | 11.8 | 10.5 | 9.8 | 2.3 | 122.5 | 2.5 | 8.8 | 1.3 | 33.2 |
| Wama/238335 | G80 | 118.5 | 84.0 | 9.8 | 8.1 | 9.0 | 1.8 | 114.0 | 2.4 | 10.1 | 1.4 | 21.7 |
| Wama/24394 | G81 | 127.3 | 97.5 | 11.0 | 9.0 | 10.0 | 2.8 | 123.3 | 1.6 | 8.8 | 1.4 | 29.4 |
| Wama/AxumColl21 | G82 | 120.0 | 102.0 | 13.5 | 11.8 | 11.5 | 2.0 | 129.5 | 0.9 | 4.6 | 1.8 | 29.2 |
| Wama/Bako-09 | G83 | 104.8 | 95.0 | 9.3 | 8.1 | 9.0 | 0.5 | 113.5 | 3.4 | 13.2 | 1.5 | 31.3 |
| Wama/Gute | G84 | 126.8 | 77.5 | 10.8 | 9.1 | 9.0 | 1.5 | 107.5 | 1.9 | 6.0 | 1.9 | 29.8 |
| Wama/Kako-1 | G85 | 135.3 | 92.3 | 9.1 | 7.3 | 7.5 | 1.0 | 120.8 | 2.3 | 7.2 | 1.8 | 25.8 |
| Wama/Meba | G86 | 111.5 | 84.0 | 11.0 | 10.3 | 8.5 | 2.0 | 113.3 | 0.7 | 4.4 | 1.1 | 23.5 |
| Wama/Mereb-1 | G87 | 116.3 | 96.5 | 7.8 | 6.4 | 7.3 | 1.8 | 124.3 | 1.3 | 4.6 | 1.5 | 27.9 |
| Mean |  | 112.9 | 90.1 | 10.2 | 8.7 | 9.4 | 2.0 | 116.8 | 1.8 | 8.1 | 1.4 | 24.8 |
| Minimum |  | 88.0 | 59.8 | 6.3 | 5.3 | 7.3 | 0.3 | 89.8 | 0.2 | 0.5 | 0.6 | 16.9 |
| Maximum |  | 140.3 | 102.3 | 14.3 | 12.8 | 14.0 | 4.8 | 129.5 | 4.4 | 18.9 | 2.3 | 40.7 |

Note: Plant height, cm (PTH), days to flowering (DTF), ear length, cm (EL), length of the longest finger, cm (FL), number of productive basal tillers per main plant (NT), the number of fingers on the main ear (NF), days to maturity (DTM), grain yield, t/ha (GY), harvest index (%) (HI), thousand seed weight (g) (TSW), and chlorophyll content at maturity (SPADM, chlorophyll content).

Supplementary Table S5. Mean performance of crossed under non- stressed conditions for agronomic and physiological traits

| Cross/family | Code ofof cross | PTH | DTF | EL | FL | NF | NT | DTM | GY | HI(%) | TSW | SPADM |
| --- | --- | --- | --- | --- | --- | --- | --- | --- | --- | --- | --- | --- |
| 227974/213835 | G18 | 120.8 | 92.8 | 16.5 | 14.3 | 11.5 | 4.3 | 131.3 | 5.1 | 11.9 | 1.9 | 35.3 |
| 227974/215982 | G19 | 128.5 | 84.5 | 14.0 | 11.3 | 10.0 | 3.8 | 125.8 | 3.2 | 11.7 | 1.7 | 34.5 |
| 227974/238335 | G20 | 125.3 | 92.8 | 15.8 | 14.3 | 10.3 | 2.8 | 132.0 | 3.8 | 10.8 | 1.9 | 34.7 |
| 227974/24394 | G21 | 137.5 | 93.8 | 15.0 | 13.8 | 9.0 | 5.5 | 138.5 | 4.2 | 12.8 | 2.1 | 31.0 |
| 227974/AxumColl21 | G22 | 127.3 | 91.0 | 12.5 | 10.3 | 11.3 | 4.3 | 128.0 | 3.1 | 10.3 | 1.9 | 27.9 |
| 227974/Bako-09 | G23 | 104.3 | 74.0 | 6.5 | 5.3 | 10.5 | 5.3 | 126.0 | 4.8 | 16.2 | 2.2 | 46.2 |
| 227974/Gute | G24 | 139.3 | 93.0 | 16.9 | 14.5 | 10.5 | 7.3 | 132.5 | 3.9 | 13.0 | 1.7 | 34.0 |
| 227974/Kako-1 | G25 | 130.5 | 96.8 | 13.5 | 14.0 | 11.5 | 4.5 | 145.0 | 3.8 | 12.0 | 2.0 | 31.2 |
| 227974/Meba | G26 | 142.3 | 102.0 | 16.0 | 13.3 | 11.0 | 4.3 | 141.3 | 5.5 | 16.1 | 2.0 | 31.6 |
| 227974/Mereb-1 | G27 | 142.0 | 96.3 | 14.3 | 12.3 | 10.3 | 4.0 | 138.5 | 4.3 | 11.4 | 1.8 | 30.0 |
| 234187/213835 | G28 | 120.0 | 93.0 | 12.5 | 12.0 | 11.0 | 4.5 | 127.5 | 4.2 | 13.4 | 2.0 | 35.6 |
| 234187/215982 | G29 | 116.8 | 89.5 | 13.5 | 11.0 | 9.5 | 5.3 | 139.5 | 5.8 | 17.3 | 2.2 | 41.6 |
| 234187/238335 | G30 | 117.5 | 90.8 | 11.6 | 12.3 | 10.3 | 6.5 | 130.5 | 6.1 | 15.5 | 2.4 | 32.1 |
| 234187/24394 | G31 | 116.5 | 86.3 | 13.5 | 11.0 | 8.3 | 3.5 | 145.5 | 5.7 | 20.7 | 2.3 | 42.3 |
| 234187/AxumColl21 | G32 | 130.3 | 92.5 | 16.8 | 14.3 | 9.0 | 6.5 | 129.5 | 5.8 | 17.2 | 1.9 | 33.1 |
| 234187/Bako-09 | G33 | 108.5 | 100.3 | 11.5 | 9.9 | 10.0 | 8.5 | 141.0 | 3.6 | 11.8 | 1.7 | 36.4 |
| 234187/Gute | G34 | 124.8 | 91.3 | 11.3 | 10.1 | 7.5 | 6.3 | 120.8 | 5.5 | 13.3 | 2.3 | 40.3 |
| 234187/Kako-1 | G35 | 121.3 | 93.8 | 12.0 | 10.5 | 10.3 | 3.0 | 135.3 | 5.0 | 12.4 | 2.2 | 32.3 |
| 234187/Meba | G36 | 113.3 | 91.8 | 12.8 | 10.5 | 9.8 | 6.5 | 121.8 | 5.4 | 18.0 | 2.0 | 31.1 |
| 234187/Mereb-1 | G37 | 110.8 | 89.5 | 12.3 | 10.5 | 7.0 | 5.5 | 129.3 | 4.0 | 14.4 | 1.7 | 32.3 |
| 235700/213835 | G38 | 100.5 | 74.0 | 5.5 | 5.1 | 12.5 | 5.8 | 118.3 | 6.1 | 23.5 | 2.1 | 40.8 |
| 235700/215982 | G39 | 84.0 | 75.3 | 7.0 | 6.5 | 8.3 | 2.5 | 105.3 | 4.5 | 16.0 | 1.8 | 37.9 |
| 235700/238335 | G40 | 107.0 | 74.5 | 7.0 | 5.5 | 9.5 | 6.5 | 120.3 | 5.9 | 15.3 | 2.2 | 37.5 |
| 235700/24394 | G41 | 138.8 | 65.5 | 15.5 | 14.1 | 9.8 | 6.0 | 109.0 | 6.3 | 13.8 | 2.0 | 26.5 |
| 235700/AxumColl21 | G42 | 129.0 | 83.8 | 12.3 | 9.8 | 11.5 | 5.5 | 137.0 | 2.9 | 8.8 | 2.2 | 29.5 |
| 235700/Bako-09 | G43 | 100.8 | 71.5 | 7.3 | 6.0 | 10.5 | 4.8 | 122.0 | 5.6 | 18.0 | 1.9 | 38.0 |
| 235700/Gute | G44 | 107.8 | 84.3 | 7.3 | 6.3 | 13.3 | 4.8 | 114.3 | 6.1 | 19.5 | 2.6 | 38.8 |
| 235700/Kako-1 | G45 | 106.8 | 77.0 | 10.3 | 7.4 | 10.3 | 5.5 | 116.3 | 4.3 | 13.5 | 2.8 | 34.0 |
| 235700/Meba | G46 | 117.8 | 75.8 | 8.0 | 7.0 | 9.0 | 8.0 | 105.8 | 5.5 | 18.5 | 2.7 | 31.9 |
| 235700/Mereb-1 | G47 | 100.5 | 95.8 | 5.0 | 4.8 | 13.0 | 10.8 | 139.5 | 5.3 | 14.6 | 1.7 | 35.0 |
| Addis-01/213835 | G48 | 109.8 | 99.5 | 7.8 | 6.8 | 8.5 | 3.5 | 129.5 | 5.6 | 16.9 | 2.1 | 28.0 |
| Addis-01/215982 | G49 | 107.3 | 94.3 | 7.0 | 5.8 | 8.5 | 3.5 | 135.0 | 4.8 | 15.7 | 1.7 | 22.9 |
| Addis-01/238335 | G50 | 113.8 | 93.5 | 7.8 | 5.8 | 8.0 | 3.8 | 134.0 | 6.4 | 17.4 | 1.8 | 33.7 |
| Addis-01/24394 | G51 | 113.0 | 102.3 | 7.5 | 6.0 | 9.0 | 7.0 | 140.0 | 5.2 | 16.3 | 1.6 | 34.4 |
| Addis-01/AxumColl21 | G52 | 114.0 | 96.3 | 9.8 | 8.8 | 8.0 | 5.3 | 144.8 | 4.3 | 13.6 | 2.4 | 29.1 |
| Addis-01/Bako-09 | G53 | 95.3 | 76.8 | 8.0 | 6.3 | 10.5 | 4.5 | 106.8 | 2.7 | 10.2 | 1.9 | 28.3 |
| Addis-01/Gute | G54 | 113.0 | 94.5 | 6.8 | 6.8 | 7.0 | 3.5 | 147.5 | 5.5 | 17.1 | 1.9 | 30.5 |
| Addis-01/Kako-1 | G55 | 106.3 | 97.8 | 6.3 | 5.8 | 7.5 | 3.8 | 139.8 | 5.5 | 14.9 | 2.4 | 27.4 |
| Addis-01/Meba | G56 | 111.3 | 89.5 | 6.8 | 5.5 | 7.8 | 6.0 | 129.3 | 6.1 | 13.4 | 2.0 | 33.8 |
| Addis-01/Mereb-1 | G57 | 109.5 | 96.3 | 7.5 | 5.5 | 10.0 | 4.0 | 135.3 | 5.6 | 15.9 | 2.1 | 28.7 |
| Necho/213835 | G58 | 119.0 | 95.3 | 10.8 | 9.8 | 8.5 | 2.5 | 128.8 | 1.3 | 4.4 | 2.3 | 25.6 |
| Necho/215982 | G59 | 109.8 | 102.0 | 12.5 | 9.5 | 11.0 | 4.0 | 139.8 | 2.1 | 5.3 | 2.1 | 25.4 |
| Necho/238335 | G60 | 117.3 | 91.5 | 14.0 | 11.5 | 10.5 | 3.3 | 121.5 | 2.5 | 7.1 | 1.6 | 29.6 |
| Necho/24394 | G61 | 107.5 | 100.0 | 14.0 | 12.0 | 12.8 | 3.0 | 140.3 | 3.8 | 6.0 | 2.5 | 39.0 |
| Necho/AxumColl21 | G62 | 122.5 | 92.8 | 14.8 | 12.0 | 9.8 | 5.3 | 142.5 | 4.4 | 11.2 | 2.1 | 27.0 |
| Necho/Bako-09 | G63 | 120.8 | 96.5 | 12.5 | 10.3 | 10.0 | 3.8 | 134.5 | 4.3 | 13.7 | 2.7 | 30.0 |
| Necho/Gute | G64 | 141.3 | 90.5 | 9.8 | 9.0 | 9.0 | 4.5 | 130.5 | 7.3 | 14.8 | 2.0 | 30.9 |
| Necho/Kako-1 | G65 | 129.0 | 93.5 | 14.8 | 12.3 | 10.0 | 4.0 | 131.8 | 2.0 | 5.7 | 2.2 | 28.5 |
| Necho/Meba | G66 | 125.8 | 102.3 | 14.0 | 10.5 | 11.5 | 2.8 | 141.8 | 4.2 | 11.3 | 2.0 | 30.1 |
| Necho/Mereb-1 | G67 | 120.8 | 97.8 | 11.3 | 9.3 | 9.3 | 7.0 | 127.8 | 3.6 | 10.4 | 1.9 | 32.6 |
| Tessema/213835 | G68 | 135.8 | 97.8 | 9.8 | 9.0 | 10.0 | 2.8 | 133.8 | 4.9 | 13.1 | 2.5 | 23.0 |
| Tessema/215982 | G69 | 126.3 | 96.8 | 8.3 | 7.3 | 8.5 | 2.3 | 126.8 | 6.0 | 14.3 | 2.6 | 24.4 |
| Tessema/238335 | G70 | 132.8 | 86.3 | 10.0 | 9.0 | 9.5 | 4.3 | 116.3 | 4.3 | 12.9 | 2.2 | 27.5 |
| Tessema/24394 | G71 | 133.0 | 92.0 | 13.0 | 10.0 | 9.3 | 3.8 | 128.8 | 5.1 | 14.6 | 2.4 | 26.9 |
| Tessema/AxumColl21 | G72 | 129.3 | 81.3 | 12.0 | 10.5 | 11.0 | 5.8 | 140.5 | 5.5 | 13.8 | 2.9 | 34.4 |
| Tessema/Bako-09 | G73 | 119.8 | 91.8 | 12.5 | 10.8 | 9.5 | 3.8 | 121.8 | 2.5 | 7.9 | 2.0 | 29.1 |
| Tessema/Gute | G74 | 133.8 | 89.5 | 8.8 | 9.3 | 8.3 | 4.3 | 119.5 | 6.4 | 17.4 | 2.8 | 36.2 |
| Tessema/Kako-1 | G75 | 140.8 | 93.0 | 8.0 | 7.3 | 8.8 | 4.5 | 132.3 | 3.3 | 7.9 | 3.0 | 37.4 |
| Tessema/Meba | G76 | 116.5 | 97.5 | 9.8 | 8.5 | 7.5 | 2.8 | 147.0 | 3.7 | 8.7 | 2.4 | 29.5 |
| Tessema/Mereb-1 | G77 | 127.0 | 100.3 | 8.3 | 7.0 | 8.0 | 3.3 | 130.3 | 6.4 | 14.8 | 3.1 | 24.4 |
| Wama/213835 | G78 | 122.0 | 92.3 | 7.8 | 6.6 | 8.0 | 3.8 | 135.5 | 5.8 | 14.5 | 2.1 | 28.1 |
| Wama/215982 | G79 | 126.0 | 91.3 | 13.5 | 10.8 | 9.8 | 5.0 | 121.3 | 6.4 | 17.1 | 2.8 | 41.2 |
| Wama/238335 | G80 | 152.0 | 86.0 | 9.5 | 8.3 | 8.3 | 4.3 | 134.0 | 6.4 | 14.3 | 2.2 | 30.9 |
| Wama/24394 | G81 | 136.5 | 103.3 | 7.8 | 6.3 | 7.5 | 4.8 | 134.8 | 4.8 | 12.6 | 1.8 | 34.7 |
| Wama/AxumColl21 | G82 | 133.0 | 90.0 | 11.8 | 9.8 | 11.5 | 4.0 | 120.0 | 1.9 | 6.1 | 2.2 | 39.2 |
| Wama/Bako-09 | G83 | 114.3 | 94.8 | 10.0 | 7.5 | 8.3 | 3.0 | 133.3 | 6.4 | 15.6 | 2.7 | 38.9 |
| Wama/Gute | G84 | 136.5 | 80.0 | 14.8 | 11.8 | 10.8 | 5.3 | 126.3 | 6.0 | 16.1 | 2.1 | 36.1 |
| Wama/Kako-1 | G85 | 138.8 | 93.5 | 8.5 | 7.8 | 6.0 | 3.5 | 143.8 | 7.2 | 15.1 | 2.7 | 39.7 |
| Wama/Meba | G86 | 124.3 | 80.8 | 6.0 | 7.0 | 9.0 | 5.5 | 110.8 | 2.1 | 7.6 | 2.2 | 33.5 |
| Wama/Mereb-1 | G87 | 151.0 | 90.8 | 8.5 | 7.5 | 6.3 | 4.8 | 129.5 | 4.8 | 13.1 | 2.5 | 35.4 |
| Mean |  | 121.5 | 90.6 | 10.8 | 9.3 | 9.5 | 4.7 | 130.2 | 4.7 | 13.4 | 2.2 | 32.7 |
| Minimum |  | 84.0 | 65.5 | 5.0 | 4.8 | 6.0 | 2.3 | 105.3 | 1.3 | 4.4 | 1.6 | 22.9 |
| Maximum |  | 152.0 | 103.3 | 16.9 | 14.5 | 13.3 | 10.8 | 147.5 | 7.3 | 23.5 | 3.1 | 46.2 |

Note: Plant height, cm (PTH), days to flowering (DTF), ear length, cm (EL), length of the longest finger, cm (FL), number of productive basal tillers per main plant (NT), the number of fingers on the main ear (NF), days to maturity (DTM), grain yield, t/ha (GY), harvest index (%) (HI), thousand seed weight (g) (TSW), and chlorophyll content at maturity (SPADM, chlorophyll content).

Supplementary Table S6. Mid-parent heterosis (MPH, %) for agronomic and physiological traits of 70 finger millet crosses under stressed and non-stressed growing conditions in greenhouse and field environments.

| Codes of crosses | Traits | | | | | | | | | | | | | | | | | | | | | | |
| --- | --- | --- | --- | --- | --- | --- | --- | --- | --- | --- | --- | --- | --- | --- | --- | --- | --- | --- | --- | --- | --- | --- | --- |
|  | **PTH** | | **DTF** | | **EL** | | **FL** | | **NT** | | **NF** | | **DTM** | | **GY** | | **HI** | | **TSW** | | **SPADM** | |  |
|  | **Growing conditions** | | | | | | | | | | | | | | | | | | | | | | |
|  | **ST** | **NST** | **ST** | **NST** | **ST** | **NST** | **ST** | **NST** | **ST** | **NST** | **ST** | **NST** | **ST** | **NST** | **ST** | **NST** | **ST** | **NST** | **ST** | **NST** | **ST** | **NST** |  |
| G18 | -5.9** | -9.1 | -10.2** | -1.5 | 11.1** | 32.7** | 12.2** | 45.2** | 0 | 6.2** | 3.0** | 19.5** | -5.7 | 2.3 | 69.7** | 64.7** | 107.5** | 31.4** | 65.6** | 14.8** | 16.8** | 18.8** |  |
| G19 | 5.3** | -5.6 | -35.8** | -13.3** | 8.3** | -0.9 | 16.7** | -0.5 | 136.4** | 0 | 9.3** | -7.0** | -26.1** | -7.1 | 88.3** | -32.9** | 129.2** | -0.4 | 24.6** | -1.8** | -7.6** | 15.5** |  |
| G20 | -21.0** | -1.4 | 7.8* | -2.6 | -32.1** | 46.5** | -29.2** | 68.9** | 300.0** | -26.7** | -20.5** | 3.8** | -0.9 | 2 | -54.3** | -32.4** | -43.3** | -25.8** | 24.3** | -12.4** | 28.3** | 11.8** |  |
| G21 | 4.3** | 4 | 8.4* | 7 | 35.8** | 37.1** | 18.9** | 63.0** | 77.8** | 63.0** | 26.2** | -1.4 | 6 | 7.9 | -18.1** | -3.4** | -24.4** | 1.5 | 32.3** | -2.6** | -27.1** | -3.7 |  |
| G22 | -14.4** | -6.5 | 3.2 | 2 | -33.9** | -22.2** | -21.7** | -12.3** | -23.1** | 13.3** | -24.1** | 9.7** | 0.7 | -6.5 | -72.2** | -40.8** | -78.1** | -24.1** | 1.7** | -8.4** | -9.5** | -14.6** |  |
| G23 | 10.3** | -11.9* | 9.6** | -18.8** | -25.3** | -46.7** | -23.8** | -47.8** | 40.0** | 35.5** | -5.7** | 2.4* | 1,0 | -3.2 | -38.0** | 4.5** | -40.8** | 14.1** | 26.5** | 3.6** | -19.7** | 36.5** |  |
| G24 | 5.8** | 7.9 | 5.3 | -4.7 | -1.1 | 36.8** | -4.0** | 49.7** | 100.0** | 100.0** | 25.0** | 21.7** | 4.6 | 0.6 | 23.9** | -25.2** | 84.2** | 3.5 | 48.2** | 3.5** | 25.2** | 20.8** |  |
| G25 | 7.5** | 5.7 | 4.3 | 6.5 | 37.3** | 15.5** | 47.8** | 52.4** | 14.3** | 2.9** | 48.5** | 15.0** | 3.2 | 10.8 | 28.0** | -8.8** | 45.4** | 0.3 | 63.9** | -10.3** | 15.5** | 9.0** |  |
| G26 | -3.9** | 11.8* | 4 | 9.0* | 35.0** | 44.6** | 30.4** | 54.7** | 7.7** | 17.2** | 27.5** | 20.5** | -3.9 | 2.4 | -19.8** | 34.3** | 20.6** | 33.2** | 88.8** | -3.3** | 19.5** | 6.1 |  |
| G27 | -11.4** | 15.3** | -10.9** | 14.2** | -19.3** | 32.6** | -25.3** | 39.0** | 53.8** | -5.9** | 16.7** | 7.9** | -7.2 | 17 | 75.2** | 14.0** | 111.7** | 0.3 | 63.8** | 6.9** | 18.7** | -6.4 |  |
| G28 | -15.4** | -2.6 | 3.2 | -1.7 | -4.5* | 1 | 8.3** | 20.0** | -37.5** | -16.3** | 56.3** | 20.5** | 1.6 | -2.1 | -76.4** | 33.6** | -70.2** | 25.0** | 41.0** | 8.7** | -32.1** | -1.6 |  |
| G29 | 3.7** | -7.7 | 5 | -8.7* | -25.4** | -4.0* | -35.8** | -4.3** | -52.9** | 2.4* | -1.4 | -7.3** | 6.7 | 1.5 | -28.8** | 21.34* | -18.6** | 28.7** | -33.1** | 13.5** | -25.3** | 14.8** |  |
| G30 | -6.3** | 0.1 | 13.0** | -5.2 | -18.1** | 8.3** | -6.5** | 42.0** | -14.3** | 26.8** | -18.3** | 9.3** | 7.5 | -0.7 | -64.6** | 7.5** | -58.8** | -4.9* | 9.8** | 1.9** | -2.9 | -14.3** |  |
| G31 | -11.4** | -5 | 14.3** | -2.1 | 18.7** | 24.1** | 15.5** | 27.5** | -33.3** | -26.3** | 23.8** | -4.3** | 9.9 | 11.6 | -10.1** | 28.9** | 30.2** | 44.4** | -1.4 | -3.1** | -20.2** | 9.7** |  |
| G32 | 9.9** | 3 | -2.3 | 3.1 | 2.4 | 4.7** | -1.1 | 20.0** | -36.8** | 26.8** | 1.3 | -7.7** | -1 | -6.7 | -48.2** | 10.7** | -49.6** | 12.1** | -36.3** | -16.2** | -33.2** | -15.5** |  |
| G33 | 13.7** | -0.2 | 4.1 | 9.4** | 5.7** | -5.1** | 2.1* | -3.7** | 25.0** | 61.9** | -2.9** | 2.6** | 3.2 | 6.7 | -22.1** | -22.0** | -25.6** | -25.4** | 2.4** | -28.1** | -3.3 | -9.5** |  |
| G34 | -15.8** | 4.5 | -21.3** | -7 | -36.8** | -8.2** | -36.1** | 2.5 | -52.9** | 25.0** | 32.3** | -7.7** | -12.5* | -9.7 | 101.9** | 4.4** | 172.3** | -6.3** | 7.8** | 24.5** | 26.6** | 16.5** |  |
| G35 | 2.7** | 6.5 | 10.2** | 2.6 | -34.6** | 3.2** | -33.3** | 12.0** | -20.0** | -47.8** | -9.4** | 7.9** | 2.2 | 1.8 | 37.6** | 19.2** | 41.6** | -8.9** | 14.0** | -8.8** | -32.5** | -7.8* |  |
| G36 | -1.5 | -3.7 | 12.7** | -2.5 | 1.3 | 15.9** | -0.8 | 20.0** | -26.3** | 30.0** | 4.5** | 13.0** | 7.9 | -13 | -78.7** | 31.5** | -82.6** | 30.7** | 22.3** | -10.7** | -26.4** | -14.2** |  |
| G37 | 2.1* | -2.4 | 24.5** | 5.6 | -7.3** | 14.6** | -13.9** | 16.7** | -57.9** | -2.2* | 5.7** | -22.2** | 17.2** | 7.4 | 7.8** | 4.7** | 1.6 | 10.8** | 42.5** | -12.5** | -40.3** | -16.1** |  |
| G38 | 5.4** | -9.4 | -7.8* | -14.4** | 62.5** | -32.3** | 64.3** | -23.1** | 0 | 7.0** | 0 | 44.9** | -3.6 | -9.1 | -41.2** | 57.0** | -40.9** | 86.8** | -33.7** | 9.9** | 4 | 14.8** |  |
| G39 | 4.3** | -26.4** | 0.9 | -16.3** | 21.5** | -28.7** | 6.4** | -20.4** | 33.3** | -514** | -8.9** | -15.4** | 0 | -23.3 | -67.8** | -18.6** | -70.2** | 4.4 | -17.1** | -9.6** | 3.5 | 6.3 |  |
| G40 | 7.2** | 1.9 | -9.5** | -15.0** | -1.6 | 8.7** | -8.7** | 4.0** | 33.3** | 26.8** | -9.1** | 7.0** | -6.4 | -8.4 | 69.4** | -8.6** | 26.2** | -15.5** | 6.8** | -8.9** | 6.3* | 1.8 |  |
| G41 | 3.6** | 25.8** | 20.9** | -18.1** | 46.9** | 134.0** | 38.8** | 167.1** | 38.5** | 26.3** | 13.0** | 20.0** | 13.0* | -16.3 | -20.7** | 21.4** | -41.1** | -14.4** | -26.9** | -19.6** | 12.5** | -30.2** |  |
| G42 | 2.1* | 13.03 | 9.6** | 2.6 | -2.2 | 4.3** | -13.4** | 14.2** | -29.4** | 7.3** | -3.6** | 24.3** | 6.4 | -1.3 | -48.4** | -51.4** | -46.3** | -48.6** | -34.7** | -2.2** | -25.4** | -23.6** |  |
| G43 | 34.6** | 4.5 | 14.4** | -14.4** | 68.2** | -7.9** | 58.3** | -13.9** | 14.3** | -9.5** | -2.7* | 13.5** | 7.6 | -7.6 | -77.1** | 3.8** | -80.6** | 1.1 | -57.9** | -18.6** | -26.6** | -4.3 |  |
| G44 | 19.0** | 0.7 | 7.7* | -6.4 | 13.9** | -9.4** | 8.9** | -4.4** | -60.0** | -5.0** | -5.9** | 73.8** | 8.1 | -14.5 | 20.2** | 1.1 | 21.0** | 21.1** | -12.5** | 40.9** | -19.7** | 14.3** |  |
| G45 | 18.5** | 5.2 | 3.1 | -7.5* | 18.2** | 40.0** | 17.1** | 22.1** | -22.2** | -4.3** | 2.9* | 13.9** | 2.9 | -12.4 | 11.4** | -13.8** | -1.4 | -12.7** | -6.1** | 15.7** | -16.2** | -1.2 |  |
| G46 | 16.3** | 11.9* | 10.4** | -11.9** | 78.9** | 18.5** | 83.3** | 29.3** | -29.4** | 60.0** | 23.3** | 10.8** | 6 | -24.4* | -48.7** | 13.6** | -47.2** | 18.3** | -41.2** | 14.6** | -17.0** | -10.5** |  |
| G47 | 10.0** | -0.6 | 24.0** | 25.0** | 54.7** | -22.3** | 36.6** | -16.1** | 17.6** | 91.1** | -13.2** | 52.9** | 15.5** | 16 | -56.4** | 16.9** | -45.1** | -2.3 | -15.5** | -11.2** | -12.8** | -7.6* |  |
| G48 | -6.1** | -5.8 | -0.3 | 7.7* | 8.8** | -15.1** | 1 | -5.3** | 0 | 0 | 10.0** | 0 | -2.5 | 2.1 | 82.3** | 40.3** | 72.4** | 58.0** | 20.0** | 14.0** | -8.9** | 1.1 |  |
| G49 | 7.3** | -10.4 | -5.7 | -1.6 | 41.7** | -35.3** | 36.4** | -33.3** | -9.1** | 7.7** | 7.2** | -11.7** | 5 | 0.7 | -89.3** | -16.2** | -92.2** | 16.6** | -25.9** | -13.6** | 7.0* | -17.9** |  |
| G50 | 16.2** | 2.8 | -13.2** | 0 | 25.9** | 4.2** | 23.4** | 0 | 100.0** | 15.4** | 7.5** | -8.6** | -8.9 | 4.7 | 78.8** | -3.3** | 65.4** | 6.7** | 60.0** | -21.6** | 16.9** | 15.9** |  |
| G51 | 0.2 | -2.5 | -6.7 | 19.1** | 36.8** | -1.6 | 16.1** | 4.3** | 211.1** | 143.5** | 42.4** | 12.5** | -4.3 | 10.2 | -64.6** | -1.9 | -63.4** | 14.0** | 21.2** | -34.6** | -4.9 | 14.1** |  |
| G52 | 9.2** | -4.8 | 13.3** | 10.0** | 27.1** | -23.5** | 27.0** | -2.8 | 23.1** | 61.5** | 26.0** | -12.3** | 9.3 | 6.8 | -53.6** | -30.3** | -49.1** | -11.0** | 7.5** | 6.7** | 33.6** | -5.2 |  |
| G53 | 11.3** | -6.6 | -5.6 | -14.1** | 6.8** | -9.9** | -2.7** | -15.2** | 120.0** | 33.3** | 46.9** | 15.1** | -3 | -17.1 | -27.8** | -51.7** | -14.7** | -35.5** | -24.5** | -18.2** | 34.0** | -11.0** |  |
| G54 | -3.7** | 0.3 | 0 | -1.4 | 63.1** | -25.0** | 50.9** | -3.6* | 154.5** | 12.0** | 44.8** | -6.7** | -8.2 | 13.1 | 65.4** | -10.8** | 56.6** | 20.2** | 1.1 | 1.6** | 34.3** | 16.6** |  |
| G55 | -4.2** | -0.8 | -19.5** | 9.7** | 11.9** | -25.4** | 2.0* | -11.5** | -28.6** | -3.2** | 23.3** | -15.5** | -13.5** | 7.9 | 82.7** | 6.9** | 96.7** | 9.4** | 24.5** | 0.8** | 35.1** | 2.8 |  |
| G56 | -13.2** | 0.3 | -16.4** | -2.6 | 7.5** | -12.9** | -5.1** | -6.4** | -7.7** | 92.0** | 42.9** | -3.1** | -11.3* | -5.4 | 12.6** | 20.7** | 64.9** | -2.9 | -23.3** | -12.6** | -3.2 | 21.6** |  |
| G57 | 2.6** | 2.6 | 18.4** | 16.7** | 75.6** | 0.8 | 64.9** | -10.2** | -23.1** | 6.7** | 12.1** | 19.4** | 12.8* | 15.6 | 7.9** | 19.3** | 6.3** | 22.4** | -64.4** | 7.7** | -1.4 | -4.5 |  |
| G58 | -9.0** | -10.4 | -2.6 | -4.3 | -9.8** | -20.4** | -13.9** | -7.1** | -60.0** | -31.0** | 20.0** | -17.1** | -7.1 | -9.2 | 483.8** | -49.5** | 460.6** | -46.8** | 26.2** | 25.0** | 40.8** | -15.3** |  |
| G59 | 10.8** | -19.3** | -4 | -0.8 | -1 | -17.7** | -6.7** | -20.8** | 63.6** | 18.5** | 4.3** | -3.3** | -2.2 | -6.2 | 8.6** | -51.3** | 37.6** | -51.6** | -4.1** | 11.2** | 22.1** | -16.3** |  |
| G60 | 14.5** | -7.6 | -5.3 | -9.1** | 24.1** | 18.5** | 12.7** | 26.0** | 250.0** | -3.7** | 1.5 | 0 | -3.2 | -15 | 74.2** | -52.9** | 81.3** | -48.4** | 6.3** | -29.8** | 39.7** | -6.4 |  |
| G61 | -10.1** | -18.6** | 1.1 | 7.5* | -11.0** | 16.7** | -22.5** | 31.5** | 11.1** | 0 | 11.9** | 30.8** | 1.2 | -1.2 | -21.0** | -1.8 | -23.2** | -49.5** | 0.3 | 4.5** | -18.1** | 19.4** |  |
| G62 | -11.2** | -9.9 | -8.2* | -2 | -40.0** | -13.8** | -41.8** | -3.0* | 38.5** | 55.6** | 12.3** | -10.3** | -5.4 | -5.3 | 87.7** | -8.0** | 140.4** | -12.8** | 18.8** | -4.9** | 34.6** | -18.7** |  |
| G63 | -3.2** | 2.1 | 5.8 | 0 | -35.7** | -5.7** | -44.7** | -4.6** | -20.0** | 7.1** | -6.3** | -8.0** | -1.8 | -6.4 | 46.4** | 3.6** | 16.0** | 2.1 | -23.9** | 18.1** | -27.1** | -12.8** |  |
| G64 | 5.0** | 9.6 | -2.6 | -12.1** | 4.4* | -27.1** | -13.6** | -13.2** | 63.6** | 38.5** | 13.8** | -2.7** | 1.6 | -10.2 | 228.0** | 52.2** | 212.9** | 26.6** | 7.5** | 8.5** | -12.6** | 7.7* |  |
| G65 | -0.2 | 4.6 | 7.6* | -2.9 | -10.7** | 15.7** | -20.0** | 24.0** | 0 | 0 | 33.3** | -5.9** | 3 | -8.8 | 172.5** | -46.2** | 223.8** | -49.1** | 43.0** | -10.2** | 9.3** | -2.1 |  |
| G66 | 15.8** | -1.1 | -18.0** | 3.3 | 8.2** | 15.5** | -0.7 | 13.5** | -7.7** | -15.4** | 14.3** | 17.9** | -11.8* | -6.5 | 65.3** | 16.5** | 39.0** | 0.1 | 53.2** | -13.5** | 36.6** | -0.7 |  |
| G67 | -6.8** | -1.8 | 15.9** | 9.1** | 20.2** | -4.7** | -1.2 | -2.6 | 115.4** | 80.6** | 69.7** | -8.6** | 0.7 | -3.2 | -28.0** | 6.2** | -38.0** | -0.8 | -13.9** | -0.3 | -2.7 | 0.1 |  |
| G68 | -14.1** | 1 | -22.0** | 4.5 | -8.3** | -7.1** | -7.6** | 2.9 | 80.0** | -37.1** | 21.3** | 14.3** | -14.1** | 8.3 | 45.1** | 58.7** | 41.5** | 45.3** | 66.4** | 7.3** | 21.3** | -22.6** |  |
| G69 | -12.7** | -8.2 | -1.4 | -0.1 | -31.0** | -32.3** | -36.5** | -29.3** | 27.3** | -45.4** | -11.4** | -13.9** | -10.8* | -3 | -8.7** | 26.1** | -22.0** | 22.2** | 15.6** | 9.3** | -3 | -18.28** |  |
| G70 | 4.1** | 3.3 | 1.6 | -8.8* | 21.7** | 13.5** | 16.4** | 22.0** | 75.0** | 3.0** | 14.7** | 5.6** | -3.1 | -6.7 | -35.6** | -24.6** | -33.9** | -11.5** | -3.1** | -21.4** | 7.4** | -11.6** |  |
| G71 | -7.5** | -0.6 | 7.2* | 5.7 | 27.8** | 44.4** | 31.3** | 35.6** | 11.1** | 0 | 26.7** | 12.1** | 3.9 | 4.1 | -24.8** | 16.9** | -3.1* | 15.7** | -47.1** | -14.1** | 4.8 | -16.4** |  |
| G72 | -2.4* | -6.1 | 5.8 | -8.3* | -37.0** | -15.0** | -35.4** | -1.2 | -38.5** | 39.4** | 8.1** | 17.3** | 2.9 | 6.3 | -39.7** | 6.0** | -35.7** | 1.3 | 25.7** | 8.1** | 14.6** | 5.3 |  |
| G73 | 31.5** | -0.1 | 3.4 | 1.4 | -1.4 | 21.9** | -8.7** | 19.4** | -20.0** | -11.7** | -7.7** | 1.3 | 2.3 | -2.9 | -9.2** | -45.2** | -40.2** | -44.2** | 17.5** | -29.0** | 2 | -13.9** |  |
| G74 | 19.1** | 2.5 | 4 | -7.7* | 26.3** | -15.7** | 35.4** | 7.2** | 63.6** | 6.2** | 25.4** | 6.4** | 6.5 | -5.9 | 41.1** | 22.8** | 53.8** | 39.2** | -34.3** | 23.3** | 4 | 28.6** |  |
| G75 | 0.3 | 12.6* | 4 | 3 | 29.7** | -17.9** | 37.3** | -10.8** | 71.4** | -5.3** | 24.6** | -4.1** | -4.3 | 4.9 | 2.4** | -20.5** | 55.1** | -34.1** | -6.6** | 4.0** | 18.6** | 30.9** |  |
| G76 | 3.8** | -9.5 | -23.0** | 4.8 | 20.9** | 6.8** | 18.3** | 13.3** | 130.8** | -31.2** | 31.3** | -9.1** | -16.6** | 10.3 | 15.0** | -9.4** | 46.9** | -27.7** | 74.9** | -11.4** | 44.7** | -1 |  |
| G77 | -9.9** | 1.9 | 11.4** | 19.8** | -33.9** | -6.4** | -33.8** | -9.7** | 84.6** | -29.7** | -7.5** | -7.2** | 7.4 | 14.6 | -29.0** | 69.3** | -16.4** | 31.2** | -37.0** | 28.2** | -4.2 | -23.8** |  |
| G78 | 3.3** | -1 | 0.3 | -2.1 | 15.1** | -31.1** | 9.6** | -27.4** | -75.0** | -6.2** | 50.0** | 10.3** | -2 | 9 | 3.6** | 58.9** | -68.3** | 28.8** | 16.9** | -13.3** | -8.1** | -9.0** |  |
| G79 | -8.1** | -0.4 | -14.2** | -6.5 | 4.1* | 4.3** | 12.5** | 1.2 | 77.8** | 33.3** | 4.6** | 16.4** | -8.8 | -7.7 | -54.8** | 19.4** | -68.2** | 22.2** | -28.1** | 10.5** | 13.4** | 32.5** |  |
| G80 | 13.6** | 29.5** | -1 | -9.8** | 54.9** | -0.6 | 63.3** | 6.4** | 233.3** | 13.3** | 30.2** | 10.0** | 3.7 | 6.9 | -9.9** | 2.2* | -55.1** | -15.2** | -32.0** | -25.5** | -4.7 | -4.4 |  |
| G81 | -9.7** | 11.3 | 6.7 | 17.7** | 13.7** | -20.5** | 4.9** | -19.3** | 42.9** | 40.7** | 45.5** | 11.1** | -4.3 | 8.3 | -51.3** | -3.6** | -78** | -15.0** | -48.9** | -39.3** | -9.2** | 4.1 |  |
| G82 | -0.5 | 5.1 | -4 | 0.7 | 0.5 | -21.0** | 8.8** | -11.4** | 45.5** | 6.7** | 13.0** | 46.0** | -1.3 | -9.7 | 67.8** | -67.0** | -5.2** | -61.3** | -8.4** | -20.8** | 33.8** | 15.5** |  |
| G83 | -0.6 | 5.1 | -8.1* | 3.8 | -30.1** | -9.1** | -32.7** | -20.0** | 375.0** | -22.6** | 60.0** | 4.7** | -4.4 | 5.6 | -0.1 | 23.7** | -31.1** | -4.6* | -13.5** | -5.5** | 54.8** | 10.9** |  |
| G84 | -5.8** | 14.3* | -0.5 | -18.2** | -35.5** | 32.6** | -20.2** | 30.6** | 77.8** | 44.8** | 22.2** | 72.0** | 2.7 | -1.2 | 10.0** | 4.4** | -51.9** | 9.3** | -18.3** | -10.6** | 25.3** | 22.9** |  |
| G85 | 5.3** | 21.8** | 2.6 | 2.7 | 23.1** | -19.0** | 45.1** | -8.8** | 150.0** | -20.0** | 35.7** | -21.3** | 1.8 | 13.3 | -14.8** | 51.4** | -66.4** | 6.9** | -23.8** | -9.9** | 1.5 | 33.1** |  |
| G86 | -5.2** | 5.6 | 4.9 | -13.8** | 4.5* | -39.2** | -2.5* | -11.1** | -27.3** | 51.7** | 18.6** | 33.3** | 1.6 | -17.3 | -45.3** | -54.6** | -73.2** | -46.9** | 23.7** | -23.6** | -5.5* | 7.8* |  |
| G87 | 0.5 | 33.0** | 16.1** | 7.6* | -16.2** | -11.1** | -20.2** | -7.7** | 27.3** | 11.7** | -6.5** | -12.3** | 12.8* | 13.2 | -41.3** | 9.9** | -72.4** | -3.2 | -9.0** | -0.1 | 1.8 | 6.3 |  |
| MSE | 109.7 | 68.1 | 24.2 | 26.1 | 8.7 | 5.4 | 6.9 | 4.2 | 0.7 | 2 | 2.6 | 3.8 | 60.2 | 279.2 | 0.2 | 2.16 | 4.7 | 10.2 | 0 | 0.1 | 15.6** | 22 |  |

Note: Growing conditions, ST: stress, and NST: non stress growing conditions, * and ** denotes significant at 5 and 1% level of significance, respectively, plant height (cm, PTH), days to flowering (DTF), ear length, (cm, (EL), length of the primary finger, (cm, FL), number of productive tillers per plant (NT), the number of fingers on the primary ear (NF), days to maturity (DTM), grain yield (t/ha, GY), harvest index (%, HI), thousand seed weight (g, TSW), and SPADM, chlorophyll content at maturity.

Supplementary Table S7. Better-parent heterosis (BPH, %) for agronomic and physiological traits of 70 finger millet crosses evaluated under stressed and non-stressed growing conditions in greenhouse and field environments.

| Codes of crosses | Traits | | | | | | | | | | | | | | | | | | | | | | |
| --- | --- | --- | --- | --- | --- | --- | --- | --- | --- | --- | --- | --- | --- | --- | --- | --- | --- | --- | --- | --- | --- | --- | --- |
|  | **PTH** | | **DTF** | | **EL** | | **FL** | | **NT** | | **NF** | | **DTM** | | **GY** | | **HI** | | **TSW** | | **SPADM** | |  |
|  | **Growing conditions** | | | | | | | | | | | | | | | | | | | | | | |
|  | **ST** | **NST** | **ST** | **NST** | **ST** | **NST** | **ST** | **NST** | **ST** | **NST** | **ST** | **NST** | **ST** | **NST** | **ST** | **NST** | **ST** | **NST** | **ST** | **NST** | **ST** | **NST** |  |
| G18 | -12.6 | -12.5* | -15.4** | -1.6 | -11.8** | 16.8** | -9.3** | 31.0** | -16.7** | 6.2** | -8.1** | 4.5** | -8.1 | -0.7 | 20.3** | 17.4** | 50.5** | 3.5 | 54.1** | 14.4** | 15.2** | 11.7** |  |
| G19 | 4.1 | -6.9 | -38.6** | -16.3** | 2.0* | -0.9 | 14.0** | -4.3** | 116.7** | -6.2** | 7.9** | -9.1** | -28.6** | -9.2 | 55.7** | -38.9** | 85.0** | -3.0 | -3.3** | -6.2** | -17.1** | 8.1* |  |
| G20 | -28.3** | -9.2 | 4.5 | -3.9 | -48.0** | 11.5** | -46.5** | 31.0** | 166.7** | -31.2** | -21.6** | -6.8** | -3.3 | -0.2 | -56.4** | -45.5** | -43.4** | -39.0** | 1.8 | -28.5** | 22.7** | 1.1 |  |
| G21 | 0.8 | -0.4 | 3.7 | -0.3 | 7.8** | 6.2** | 2.3* | 26.4** | 33.3** | 37.5** | 10.8** | -18.2** | 3.2 | 4.7 | -33.3** | -4.3** | -42.0** | -7.0** | -5.0** | -21.7** | -33.9** | -15.2** |  |
| G22 | -18.5** | -7.8 | 3.1 | -3.2 | -37.9** | -30.6** | -26.5** | -18.0** | -28.6** | 6.2** | -28.6** | 2.3* | 0.6 | -9.5 | -74.0** | -49.5** | -81.0** | -34.5** | -27.1** | -22.9** | -10.6** | -25.7** |  |
| G23 | -8.5 | -24.5** | 7.9* | -21.3** | -39.2** | -54.0** | -34.9** | -51.7** | 16.7** | 31.2** | -10.8** | -4.5** | -0.2 | -4.7 | -47.4** | -1.8 | -55.7** | -4.3 | -5.6** | -14.4** | -27.9** | 15.9** |  |
| G24 | 0.2 | 0.9 | 1.0 | -8.1* | -13.7** | 19.3** | -16.3** | 33.3** | 83.3** | 81.2** | 8.1** | -4.5** | 4.0 | 0.2 | -9.5** | -36.2** | 29.0** | -4.5 | 26.5** | 1.5** | 21.4** | 19.4** |  |
| G25 | -3.1 | -5.4 | 3.4 | 2.9 | 11.8** | -4.4** | 18.6** | 28.7** | 0.0 | -5.3** | 32.4** | 4.5** | 2.6 | 9.6 | -4.7** | -11.9** | 7.3** | -3.7 | 38.5** | -28.3** | 11.6** | 6.0 |  |
| G26 | -15.0* | 3.1 | 0.8 | 8.5* | 3.9** | 13.3** | 2.3* | 21.8** | 0.0 | 6.2** | 18.9** | 0.0 | -6.1 | -1.7 | -21.7** | 26.9** | 19.1** | 26.8** | 71.2** | -19.7** | 15.8** | -0.6 |  |
| G27 | -16.3* | 2.9 | -21.4** | 2.4 | -32.4** | 0.9 | -34.9** | 12.6** | 42.9** | -11.1** | 13.5** | -6.8** | -14.9** | 4.7 | 54.8** | -0.5 | 77.9** | -0.7 | 19.3** | 2.7** | 3.3 | -17.3** |  |
| G28 | -17.5* | -6.1 | -4.0 | -2.1 | -22.1** | -10.7** | -10.0** | 6.7** | -58.3** | -33.3** | 42.9** | 10.0** | -2.0 | -6.4 | -84.8** | -5.2** | -80.9** | -9.7** | 10.7** | -2.4** | -36.8** | -12.5** |  |
| G29 | -0.4 | -13.0* | -0.8 | -11.4** | -27.4** | -4.4** | -36.6** | -6.4** | -66.7** | -22.2** | -5.3** | -9.5** | 2.0 | 0.7 | -48.4** | 11.2** | -43.9** | 16.8** | -36.8** | 6.0** | -36.4** | 2.5 |  |
| G30 | -10.9 | -1.0 | 8.2* | -6.0 | -35.8** | -17.3** | -27.5** | 8.9** | -50.0** | -3.7** | -19.4** | 2.5* | 3.9 | -4.2 | -71.2** | -12.8** | -66.8** | -12.7** | -2.8** | -8.2** | -12.2** | -21.0** |  |
| G31 | -12.9 | -7.9 | 10.7** | -9.2** | -3.2 | -3.6* | 2.5* | -2.2* | -58.3** | -48.1** | 11.4** | -17.5** | 8.1 | 6.8 | -14.0** | 28.6** | 21.6** | 39.3** | -7.0** | -14.3** | -23.4** | 4.2 |  |
| G32 | 9.8 | -3.0 | -3.7 | -2.6 | -6.9** | -6.9** | -10.2** | 14.0** | -50.0** | -3.7** | -7.1** | -10.0** | -2.1 | -8.5 | -53.7** | -5.1** | -53.6** | 8.6** | -40.0** | -21.9** | -36.2** | -18.6** |  |
| G33 | -1.5 | -8.6 | 3.8 | 5.5 | -11.6** | -17.9** | -10** | -12.2** | -16.7** | 25.9** | -5.7** | 0.0 | 3.1 | 3.5 | -22.8** | -26.2** | -32.8** | -30.1** | 2.3* | -34.3** | -8.2** | -10.4** |  |
| G34 | -16.2* | 4.0 | -25.4** | -9.9** | -43.2** | -19.6** | -42.5** | -10.0** | -66.7** | -7.4** | 17.1** | -25.0** | -13.9** | -11.4 | 32.9** | -10.5** | 69.7** | -10.4** | -8.6** | 9.5** | 15.9** | -1.0 |  |
| G35 | -3.1 | 2.1 | 9.7** | -1.3 | -45.3** | -14.3** | -45** | -6.7** | -33.3** | -55.6** | -17.1** | 2.5* | 1.7 | -0.7 | -8** | 14.3** | -8.4** | -16.2** | -2.5* | -19.8** | -38.3** | -20.5** |  |
| G36 | -8.7 | -4.6 | 7.9* | -3.4 | -20.0** | -8.9** | -20.0** | -6.7** | -41.7** | -3.7** | 0.0 | -2.5 | 4.3 | -15.3 | -81.6** | 23.5** | -85.8** | 21.2** | -1.9 | -17.9** | -32.6** | -23.5** |  |
| G37 | 1.3 | -6.7 | 11.0** | -5.8 | -20.0** | -12.5** | -22.5** | -6.7** | -66.7** | -18.5** | 5.7** | -30.0** | 8.5 | -5.1 | 2.3* | -9.1** | -3.3* | -2.7 | 37.5** | -18.7** | -45.1** | -20.6** |  |
| G38 | 0.2 | -21.3** | -19.2** | -21.5** | 52.9** | -48.8** | 55.9** | -41.4** | -30.0** | -14.8** | -14.6** | 38.9** | -10.5 | -13.0 | -64.2** | 2.9** | -65.1** | 26.6** | -51.7** | -2.9** | -6.2* | 3.5 |  |
| G39 | -6.6 | -37.4** | -10.3** | -25.5** | 6.7** | -50.4** | -8.5** | -44.7** | 0.0 | -63.0** | -12.2** | -21.4** | -8.0 | -24.0* | -78.4** | -23.0** | -81.5** | -13.7** | -29.1** | -16.9** | -14.4** | -3.8 |  |
| G40 | 4.6 | -7.7 | -18.5** | -22.8** | -11.8** | -5.1** | -20.3** | -8.3** | -20.0** | -3.7** | -14.6** | 5.6** | -13.0* | -11.6 | 24.7** | -16.0** | -11.8** | -17.4** | -13.7** | -16.6** | -6.7* | -4.8 |  |
| G41 | -5.3 | 9.7 | 17.0** | -19.4** | 38.2** | 100.0** | 35.5** | 135.4** | -10.0** | -11.1** | -4.9** | 8.3** | 10.4 | -19.8 | -27.6** | 6.0** | -48.5** | -25.4** | -30.6** | -27.8** | 11.6** | -32.7** |  |
| G42 | -5.2 | -3.9 | 1.4 | -0.9 | -22.4** | -31.9** | -30.6** | -22.0** | -40.0** | -18.5** | -4.8** | 21.0** | 1.1 | -3.2 | -58.8** | -52.3** | -58.5** | -52.4** | -37.8** | -7.3** | -31.0** | -25.3** |  |
| G43 | 24.7** | 2.0 | 7.6* | -19.0** | 63.2** | -29.3** | 55.7** | -35.7** | -20.0** | -29.6** | -12.2** | 10.5** | 3.5 | -10.3 | -80.1** | -4.8** | -82.5** | -3.3 | -62.2** | -24.4** | -28.0** | -4.8 |  |
| G44 | 11.0 | -10.2 | -3.9 | -16.8** | 7.9** | -30.9** | 4.7** | -26.5** | -70.0** | -29.6** | -22.0** | 47.2** | 2.3 | -16.0 | -25.5** | -0.7 | -29.8** | 4.8 | -31.9** | 22.1** | -28.6** | -1.5 |  |
| G45 | 16.5* | -2.1 | -3.7 | -12.2** | 14.7** | 11.6** | 10.2** | -1.7 | -30.0** | -18.5** | -12.2** | 13.9** | -1.5 | -14.5 | -30.1** | -27.5** | -41.3** | -27.1** | -26.4** | 3.3** | -25.8** | -13.7** |  |
| G46 | 16.0* | 1.1 | -0.5 | -18.8** | 61.8** | 0.0 | 67.8** | 12.0** | -40** | 18.5** | 9.8** | 0.0 | -1.4 | -26.4* | -60.3** | -6.2** | -62.8** | -0.5 | -56.4** | 7.1** | -26.3** | -19.1** |  |
| G47 | 2.9 | -7.2 | 17.5** | 21.6** | 53.6** | -32.2** | 31.3** | -29.6** | 0.0 | 59.3** | -19.5** | 44.4** | 11.1* | 2.6 | -63.4** | -9.6** | -56.5** | -21.6** | -21.5** | -18.9** | -17.3** | -11.3** |  |
| G48 | -7.8 | -14.1* | -5.0 | 5.6 | 3.3** | -27.9** | -1.9 | -22.9** | -16.7** | -12.5** | 6.5** | -2.9* | -4.0 | 0.0 | 27.5** | -9.1** | 16.2** | 14.2** | -5.3** | 1.8** | -17.9** | -11.4** |  |
| G49 | -1.0 | -20.1** | -8.7** | -6.7 | 13.3** | -50.4** | 9.8** | -51.1** | -16.7** | 0.0 | -2.6** | -19.0** | 2.4 | -2.5 | -91.3** | -22.6** | -94.2** | 5.9** | -29.6** | -19.7** | 6.1* | -28.3** |  |
| G50 | 15.2* | -1.9 | -14.8** | -3.1 | 25.9** | 3.3* | 16.0** | -4.2** | 33.3** | 7.1** | 0.0 | -8.6** | -10.2 | 3.5 | 67.0** | -9.0** | 46.3** | -2.1 | 42.4** | -29.0** | 8.3** | -1.9 |  |
| G51 | -5.5 | -10.7 | -11.8** | 13.0** | 30.0** | -3.2 | 4.8** | 0.0 | 133.3** | 133.3** | 35.5** | 2.9** | -7.7 | 8.1 | -70.7** | -16.1** | -69.0** | 10.1** | 13.8** | -41.8** | -22.3** | -5.8 |  |
| G52 | 4.6 | -15.1** | 12.1** | 6.3 | -6.9** | -45.8** | -4.1** | -30.0** | 14.3** | 50.0** | 9.5** | -15.8** | 8.4 | 2.3 | -55.5** | -30.7** | -50.3** | -13.8** | 0.6 | 0.0 | 17.5** | -22.6** |  |
| G53 | 0.0 | -9.5 | -8.2* | -15.2** | -1.6 | -21.9** | -11.5** | -32.4** | 83.3** | 20.0** | 42.4** | 10.5** | -5.0 | -17.6 | -37.4** | -56.7** | -29.9** | -39.5** | -24.9** | -24.9** | 8.6** | -28.9** |  |
| G54 | -7.3 | -5.8 | -2.8 | -6.7 | 39.5** | -35.7** | 34.4** | -20.6** | 133.3** | 7.7** | 35.5** | -20.0** | -8.6 | 12.4 | 19.0** | -11.4** | 2.5 | 15.2** | -13.9** | -11.1** | 22.8** | 7.0* |  |
| G55 | -5.7 | -2.5 | -21.2** | 8.0* | 3.1** | -32.4** | 0.0 | -23.3** | -37.5** | -21.0** | 19.4** | -16.7** | -14.9** | 7.9 | 34.1** | -11.9** | 34.5** | 0.7 | 7.1** | -11.0** | 24** | -7.0* |  |
| G56 | -16.2* | -4.5 | -18.0** | -4.0 | 6.5** | -15.6** | -6.0** | -12.0** | -14.3** | 84.6** | 40.6** | -11.4** | -12.6* | -10.1 | 12.4** | -2.2* | 47.6** | -9.8** | -38.2** | -19.2** | -11.4** | 6.2 |  |
| G57 | -0.9 | 1.1 | 3.3 | 6.3 | 56.5** | 0.0 | 46.9** | -18.5** | -28.6** | -11.1** | 5.7** | 14.3** | 2.5 | 4.4 | -2.6** | -9.4** | 0.1 | 7.6** | -65.8** | -0.6** | -22.2** | -20.9** |  |
| G58 | -13.6 | -13.6* | -5.5 | -9.1** | -28.8** | -33.8** | -33.7** | -20.4** | -66.7** | -37.5** | 16.1** | -30.6** | -8.1 | -19.3 | 350.2** | -61.2** | 328.7** | -55.6** | -0.7 | 12.6** | 33.2** | -19.1** |  |
| G59 | 9.5 | -20.3** | -5.4 | -2.6 | -7.7** | -23.1** | -14.3** | -22.4** | 50.0** | 14.3** | -5.3** | -10.2** | -4.2 | -12.4 | -26.9** | -59.9** | -4.4** | -56.1** | -9.1** | 4.2** | 16.9** | -20.3** |  |
| G60 | 6.0 | -14.9** | -5.3 | -12.6** | -5.8** | -13.8** | -18.4** | -6.1** | 133.3** | -7.1** | -5.6** | -14.3** | -4.1 | -23.8 | 8.8** | -65.0** | 12.3** | -59.9** | -5.6** | -37.0** | 36.1** | -13.9** |  |
| G61 | -11.1 | -22.0** | -6.1 | -4.5 | -29.8** | -13.8** | -36.7** | -2.0* | -16.7** | -7.7** | 6.5** | 4.1** | -2.9 | -12.1 | -54.9** | -12.8** | -56.7** | -56.8** | -5.6** | -7.9** | -30.2** | 6.9* |  |
| G62 | -13.6 | -11.1 | -10.8** | -11.5** | -43.1** | -18.1** | -41.8** | -4.0** | 28.6** | 50.0** | -2.4* | -20.4** | -6.6 | -10.7 | 12.5** | -28.3** | 41.2** | -29.3** | 11.5** | -11.7** | 24.1** | -28.2** |  |
| G63 | -18.1* | -12.3* | 1.1 | -7.9* | -48.1** | -23.1** | -55.1** | -16.3** | -33.3** | 0.0 | -9.1** | -18.4** | -4.3 | -15.7 | -15.2** | -12.2** | -35.1** | -19.3** | -24.1** | 7.5** | -38.4** | -24.9** |  |
| G64 | 1.6 | 2.5 | -3.6 | -13.6** | -9.6** | -40.0** | -28.6** | -26.5** | 50.0** | 38.5** | 6.5** | -26.5** | 0.6 | -18.2 | 144.7** | 18.6** | 149.6** | 9.2** | -8.6** | -4.3** | -16.1** | 7.0* |  |
| G65 | -8.2 | -6.4 | 3.4 | -10.7** | -27.9** | -9.2** | -38.8** | 0.0 | -12.5** | -15.8** | 29.0** | -18.4** | 0.8 | -17.4 | 99.2** | -50.2** | 143.1** | -54.4** | 22.7** | -21.3** | 5.4 | -3.1 |  |
| G66 | 4.5 | -8.7 | -18.0** | -2.4 | -17.3** | -13.8** | -25.5** | -14.3** | -14.3** | -15.4** | 12.5** | -6.1** | -12.6* | -11.1 | 0.5 | 10.0** | -14.3** | -11.1** | 23.2** | -20.7** | 31.4** | -5.4 |  |
| G67 | -10.1 | -12.3* | -0.5 | -6.7 | 0.0 | -30.8** | -18.4** | -24.5** | 100.0** | 55.6** | 60.0** | -24.5** | -8.9 | -19.9 | -57.7** | 3.9** | -63.9** | -6.9** | -17.2** | -7.2** | -20.1** | -10.2** |  |
| G68 | -18.6** | -3.7 | -25.1** | 3.7 | -21.4** | -9.3** | -16.7** | 2.9* | 50.0** | -42.1** | 15.6** | 8.1** | -14.8** | 7.6 | -6.0** | 13.2** | -7.9** | 14.7** | 33.9** | -16.7** | 13.0** | -27.3** |  |
| G69 | -13.5 | -10.5 | -3.9 | -4.2 | -33.3** | -41.6** | -42.7** | -38.3** | 16.7** | -52.6** | -18.4** | -19.0** | -12.3* | -8.5 | -33.3** | 14.6** | -45.0** | 18.6** | 12.6** | -12.4** | -5.7* | -23.5** |  |
| G70 | -3.9 | -5.8 | 0.5 | -10.6** | 0.0 | -2.4 | -3.0** | 2.9* | 16.7** | -10.5** | 8.3** | 2.7** | -3.7 | -8.1 | -47.1** | -39.3** | -45** | -27.4** | -11.8** | -26.6** | 3.0 | -20.1** |  |
| G71 | -8.8 | -5.7 | 0.5 | -0.8 | 9.5** | 26.8** | 27.3** | 14.3** | -16.7** | -21.0** | 18.8** | 0.0 | -0.6 | 3.4 | -28.9** | 15.6** | -12.9** | 5.6* | -51.5** | -18.3** | -11.8** | -26.4** |  |
| G72 | -5.3 | -8.3 | 3.8 | -12.4** | -45.7** | -33.3** | -45.9** | -16.0** | -42.9** | 21.0** | -4.8** | 15.8** | 1.2 | -0.7 | -45.4** | -9.7** | -38.5** | -12.8** | 14.9** | -2.5** | 4.1 | -8.5** |  |
| G73 | 10.9 | -15.1** | -0.3 | -1.1 | -13.1** | 21.9** | -12.1** | 16.2** | -33.3** | -21.0** | -9.1** | 0.0 | -0.6 | -4.9 | -9.6** | -48.6** | -47.8** | -53.3** | 14.1** | -34.6** | -15.0** | -27.0** |  |
| G74 | 15.0* | -5.1 | 1.8 | -11.6** | 20.2** | -16.7** | 33.3** | 5.7** | 50.0** | -10.5** | 15.6** | -10.8** | 5.1 | -9.0 | -6.5** | 4.6** | -2.5 | 28.1** | -42.8** | -5.9** | -1.6 | 27.0** |  |
| G75 | -8.0 | -0.2 | 1.1 | 0.3 | 14.3** | -21.9** | 22.7** | -17.1** | 50.0** | -5.3** | 18.8** | -5.4** | -6.6 | 2.1 | -31.1** | -23.0** | 2.2 | -36.9** | -17.9** | -0.3 | 12.6** | 27.2** |  |
| G76 | -6.6 | -17.4** | -23.8** | 4.6 | 0.0 | -4.9** | 3.0** | -2.9** | 114.3** | -42.1** | 31.3** | -18.9** | -17.1** | 2.3 | 0.2 | -14.2** | 23.7** | -31.4** | 43.7** | -19.0** | 37.0** | -7.3* |  |
| G77 | -13.3 | -9.9 | -3.5 | 8.1* | -39.8** | -19.5** | -34.8** | -20.0** | 71.4** | -31.6** | -11.4** | -13.5** | -3.1 | 6.1 | -31.8** | 48.2** | -17.3** | 30.2** | -41.0** | 2.2** | -22.3** | -32.7** |  |
| G78 | -0.8 | -4.5 | -0.7 | -2.1 | 1.3 | -34.0** | 0.3 | -30.3** | -75.0** | -6.2** | 44.8** | -3.0* | -2.4 | 9.0 | -29.6** | 6.1** | -82.1** | -8.8** | -11.0** | -34.3** | -10.7** | -11.0** |  |
| G79 | -10.3 | -6.1 | -14.7** | -9.6** | -2.2 | -4.4** | 0.0 | -8.5** | 60.0** | 25.0** | -10.5** | -7.1** | -9.9 | -12.4 | -64.7** | 17.1** | -81.3** | 7.7** | -34.7** | -13.7** | 5.7* | 29.1** |  |
| G80 | 6.3 | 28.0** | -3.0 | -10.9** | 30.4** | -19.1** | 37.9** | -13.2** | 150.0** | 6.2** | 13.9** | -5.7** | 3.5 | 5.9 | -19.7** | -9.2** | -71.0** | -19.7** | -42.0** | -32.6** | -4.9 | -10.1** |  |
| G81 | -9.7 | 7.9 | -2.8 | 9.5** | 0.0 | -34.0** | 3.4** | -34.2** | 25.0** | 18.7** | 42.9** | 3.4** | -8.8 | 8.2 | -57.8** | -13.0** | -83.0** | -20.5** | -49.7** | -44.1** | -20.8** | -4.9 |  |
| G82 | -2.1 | -0.9 | -8.6** | -4.5 | -15.5** | -34.7** | -10.2** | -22.0** | 14.3** | 0.0 | -7.1** | 21.0** | -3.3 | -15.0 | 66.6** | -68.7** | -33.6** | -61.4** | -10.0** | -30.7** | 26.7** | 4.2 |  |
| G83 | -15.2* | -3.8 | -14** | 0.5 | -36.7** | -14.9** | -34.2** | -21.0** | 375.0** | -25** | 45.5** | -13.2** | -7.6 | 4.1 | -9.5** | 17.4** | -45.2** | -7.67** | -17.2** | -15.6** | 33.9** | -2.5 |  |
| G84 | -7.8 | 13.7* | -1.5 | -21.0** | -36.7** | 25.5** | -20.3** | 23.7** | 60** | 31.3** | 22.2** | 72.0** | 1.0 | -3.8 | -23.2** | -1.2 | -73.1** | 1.3 | -33.1** | -33.3** | 23.8** | 19.3** |  |
| G85 | -2.1 | 16.8** | -3.3 | -0.8 | 11.4** | -27.7** | 31.7** | -18.4** | 87.5** | -26.3** | 31** | -33.3** | -1.0 | 11.0 | -39.4** | 31.4** | -80.9** | -4.6* | -37.1** | -16.4** | 0.7 | 31.2** |  |
| G86 | -13.5 | 4.6 | 2.8 | -14.3** | -11.4** | -48.9** | -13.8** | -26.3** | -42.9** | 37.5** | 9.4** | 24.1** | 1.4 | -23.0 | -47.9** | -61.3** | -82.6** | -52.3** | -3.9** | -32.3** | -6.5* | 5.2 |  |
| G87 | -1.9 | 27.2** | -2.0 | -3.7 | -21.5** | -27.7** | -20.3** | -21.1** | 0.0 | 5.6** | -17.1** | -21.9** | 1.4 | 4.2 | -44.5** | -12.6** | -80.3** | -17.3** | -9.7** | -22.3** | -14.5** | -2.5 |  |
| MSE | 109.7 | 68.1 | 24.2 | 26.1 | 8.7 | 5.4 | 6.9 | 4.2 | 0.7 | 2.0 | 2.6 | 3.8 | 60.2 | 279.2 | 0.2 | 2.2 | 4.7 | 10.2 | 0.0 | 0.1 | 15.6** | 22.0 |  |

Note: Growing conditions, ST: stress, and NST: non stress growing conditions, * and ** denotes significant at 5 and 1% level of significance, respectively, plant height (cm, PTH), days to flowering (DTF), ear length, (cm, (EL), length of the primary finger, (cm, FL), number of productive tillers per plant (NT), the number of fingers on the primary ear (NF), days to maturity (DTM), grain yield (t/ha, GY), harvest index (%, HI), thousand seed weight (g, TSW), and SPADM, chlorophyll content at maturity.
